# The deubiquitinase YOD1 suppresses tumor progression by stabilizing ZNF24 in clear cell renal carcinoma

**DOI:** 10.1038/s41419-025-07673-2

**Published:** 2025-04-24

**Authors:** Ji Liu, Ying Lu, Runye Zhu, Ping Xi, Zhihao Yang, Zhipeng Zhang, Yunbing Xiong, Yifu Liu, Qiqi Zhu, Ting Sun, Wenjie Xie, Binbin Gong

**Affiliations:** 1 Department of Urology, The First Affiliated Hospital, Jiangxi Medical College, Nanchang University, Nanchang, Jiangxi China; 2 Jiangxi Provincial Key Laboratory of Urinary System Diseases, Nanchang City, Jiangxi Province China; 3 Department of Clinical Laboratory, The Second Affiliated Hospital, Jiangxi Medical College, Nanchang University, Nanchang, Jiangxi China; 4 Department of Thoracic Surgery, XinSteel Center Hospital, Xinyu, China; 5 The Second Affiliated Hospital, Department of Urology, Hengyang Medical School, University of South China, Hengyang, Hunan China

**Keywords:** Renal cell carcinoma, Tumour angiogenesis, Oncogenesis

## Abstract

Metastasis remains a significant challenge in the management of clear cell renal cell carcinoma (ccRCC), and a continued focus on its underlying mechanisms is crucial for improving patient outcomes and optimizing clinical therapies. The ovarian-tumor related protease (OTU) is involved in regulating critical cell signaling pathways, but the functions of most OTUs have yet to be explored. In this study, an unbiased RNAi screening revealed that ovarian tumor domain-containing 2 (YOD1) knockdown significantly promoted cell metastasis. YOD1 downregulation promoted ccRCC growth and metastasis both in vitro and in vivo. Notably, YOD1 knockdown stimulated the growth of organoids derived from ccRCC patients. Further investigation revealed that YOD1 directly interacted with and stabilized Zinc finger protein 24 (ZNF24) expression by deubiquitination in a manner dependent on its catalytic activity. YOD1 inhibition attenuated ZNF24 transcriptional repression of vascular endothelial growth factor A (VEGFA), thereby promoting VEGFA gene expression. Furthermore, ZNF24 was identified as a key mediator of YOD1 function. The expression of YOD1 and ZNF24 was significantly downregulated in tumor tissues, with a strong correlation between them. Importantly, reduced YOD1 and ZNF24 levels were strongly associated with poor clinical outcomes in ccRCC patients. Our results reveal the mechanism by which YOD1 regulates VEGFA transcription and suppresses tumorigenesis by deubiquitinating ZNF24, providing a therapeutic target in ccRCC.

## Introduction

Renal cell carcinoma (RCC) represents a significant health challenge globally, with clear cell renal cell carcinoma (ccRCC) emerging as the predominant subtype, accounting for approximately 75% of all cases [1]. Over the last two decades, the occurrence of RCC has consistently increased worldwide [2]. Despite significant advancements in early detection and surgical interventions, a substantial proportion of RCC patients present have metastatic disease at diagnosis (approximately 30%) or develop metastasis after initial treatment for localized tumors [3]. Metastatic RCC (mRCC) remains particularly aggressive, maintaining a five-year survival rate below 10%, despite advances in targeted therapies and immunotherapies [4, 5]. Although drugs such as sunitinib have shown some benefit in delaying disease progression, their effectiveness is frequently hampered by severe side effects and they only result in modest improvements in patient outcomes [4, 6]. Therefore, a thorough investigation into the molecular mechanisms driving RCC metastasis is essential for identifying new therapeutic targets and increasing long-term survival rates. Investigating these mechanisms is essential for advancing treatment approaches.

The ubiquitin‒proteasome system (UPS) is essential for maintaining protein homeostasis in eukaryotic cells by regulating targeted protein degradation [7, 8]. Dysregulation of this system is closely associated with the pathogenesis of ccRCC. For example, VHL gene mutations in ccRCC prevent timely proteasomal degradation of HIF-α, thereby increasing the growth, metastasis, and angiogenesis of renal cancer [9–11]. Recent research has progressively emphasized the role of deubiquitinating enzymes (DUBs) in cancer invasion and metastasis, highlighting their significant impact on tumor progression [12, 13]. By counteracting the effects of ubiquitination, DUBs can exacerbate cancer development when their regulation is aberrant [14]. These findings underscore the therapeutic potential of targeting the UPS, particularly through DUBs, for innovative treatment strategies for kidney cancer. Therefore, further exploration of these pathways is essential for identifying effective therapeutic modalities.

DUBs consist of six subfamilies, with the OTU subfamily being particularly significant because of its critical roles in various cellular functions [15]. For example, OTUD5 has been demonstrated to engage the mTOR signaling pathway, which promotes cancer development and exhibiting oncogenic potential in bladder cancer [16]. Similarly, OTUD3 is essential for stabilizing the tumor suppressor PTEN, which is crucial for regulating cellular growth and survival in cancer contexts [17]. YOD1, an important member of this subfamily, has emerged as a key player in multiple malignancies, including liver and pancreatic cancers, where it regulates pathways such as the Hippo signaling axis to promote tumor cell proliferation [18, 19]. However, research on YOD1 in renal cancer is scarce, highlighting the need for investigations into its role and potential as a therapeutic target in this type of carcinoma.

In this study, we first conducted an unbiased RNAi screen targeting OTU deubiquitinating enzymes, which revealed that YOD1 deficiency significantly enhances cell migration. Additionally, YOD1 knockdown notably increased the proliferation of patient-derived renal cancer organoids, as well as ccRCC proliferation and metastasis, in vitro and in vivo. Mechanistically, YOD1 binds directly to and stabilizes ZNF24, specifically removing the K27/29/48-linked ubiquitination of ZNF24 at the K308 site. This enhances the capacity of ZNF24 to transcriptionally repress VEGFA, thereby inhibiting ccRCC progression and metastasis. Our results highlight the pivotal role of the YOD1/ZNF24/VEGFA axis in ccRCC metastasis and suggest its therapeutic potential for ccRCC treatment.

## Results

### Downregulated YOD1 expression is correlated with ccRCC progression

**Fig. 1:**
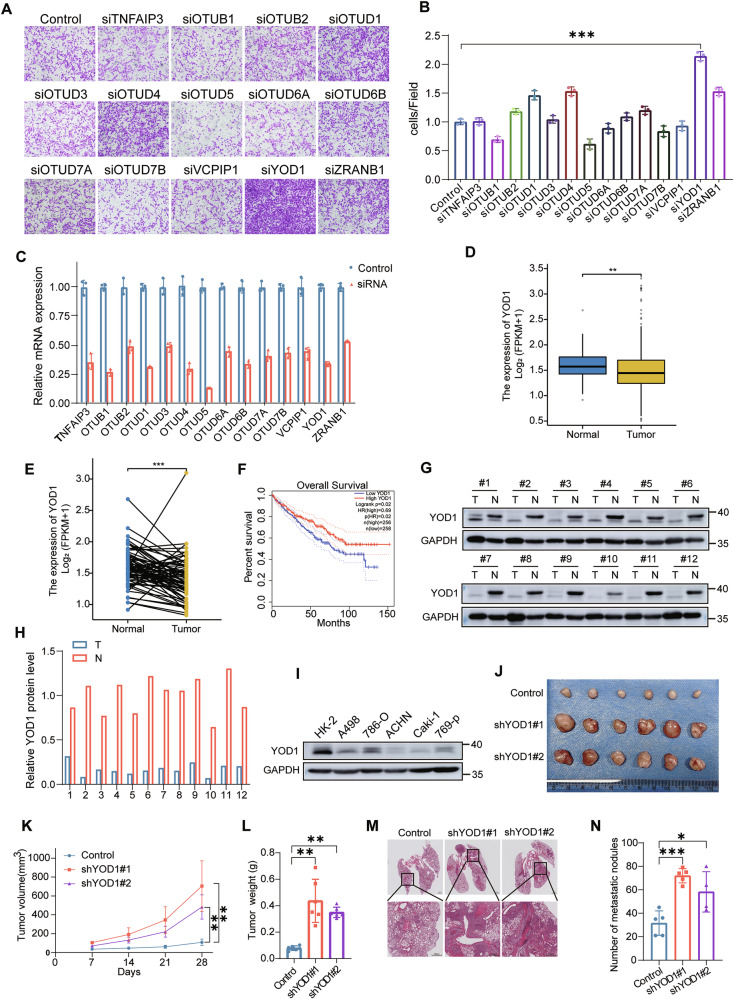
Downregulated YOD1 expression is correlated with ccRCC progression. Fourteen siRNAs were used to deplete the indicated DUBs in 786-O cells. Representative images (**A**) and quantification (**B**) of documented cell migration in a transwell assay. (Student’s *t*-test) **C** The efficiency of siRNAs knockdown was validated through real-time quantitative PCR. Differential expression analysis of YOD1 mRNA was conducted in clear cell renal carcinoma (ccRCC) tissues (*n* = 541) and adjacent normal tissues (*n* = 72) (**D**), as well as in paired ccRCC tissues (*n* = 72) and their corresponding normal tissues (*n* = 72) (**E**), using data from the TCGA database. **F** Patients with ccRCC were stratified on the basis of YOD1 expression and subjected to overall survival analysis. The data for this analysis were sourced from the TCGA database. **G**, **H** Differences in YOD1 protein levels between ccRCC tissues and corresponding normal tissues were analyzed via Western blotting. **I** Basal expression levels of the YOD1 protein were measured across various ccRCC cell lines. **J**–**L** BALB/c nude mice were injected with either control or YOD1-knockdown 786-O cells. Tumor growth was monitored at 7-day intervals, and the results are presented as the mean ± SD (*n* = 6). (ANOVA) **M**, **N** The tail veins of NCG mice were injected with either control or YOD1-knockdown 786-O cells, and the lungs were harvested 30 days postinjection. H&E staining of the dissected lungs is shown in (**M**). Scale bar, 1 mm for the upper panel and 500 μm for the lower panel. The quantification of lung nodules in each group is presented in (**N**) (5 mice per group). (ANOVA)*p < 0.05; **p < 0.01; ***p < 0.001.

To identify the OTUs involved in regulating cell metastasis, we screened 14 human OTU deubiquitinating enzymes by employing small interfering RNA (siRNA) for knockdown. Notably, cells depleted of YOD1 migrated at about twice the rate seen in the control group (Fig. 1A, B). The efficacy of the siRNAs in inducing knockdown was validated through real-time quantitative PCR, as illustrated in Fig. 1C. Furthermore, ccRCC tissue had significantly lower levels of YOD1 than normal renal tissue (TCGA data) (Fig. 1D, E, Supplementary Fig. 1A). The downregulation of YOD1 correlated with advanced pathological T stages and histologic grades, as well as advanced clinical stages (Supplementary Fig. 1B–D). Kaplan–Meier survival analysis revealed that low YOD1 expression was correlated with significantly reduced overall survival (OS) and disease-free survival (DFS) (Fig. 1F, Supplementary Fig. 1E). Western blot analyses revealed that ccRCC tissues had lower YOD1 protein levels than normal renal tissue, and the results were similar at the mRNA level (Fig. 1G, H, Supplementary Fig. 1F). In addition, YOD1 protein levels were significantly lower in ccRCC cells than in normal HK-2 cells (Fig. 1I).

Furthermore, lentivirus-mediated transfection of two YOD1-specific short hairpin RNAs (shYOD1 #1 and shYOD1 #2) was used to inhibit YOD1 expression in 786-O cells. YOD1 knockdown was confirmed by Western blotting (Supplementary Fig. 2A). These cells were then used to establish subcutaneous xenograft tumours by injection into the axilla of mice and lung metastasis models by injection into the tail vein. The results revealed that YOD1 knockdown markedly enhanced the growth of subcutaneous tumors (Fig. 1J–L) and the incidence of lung metastasis (Fig. 1M, N) in nude mice and NCG mice. The above results suggest that reduced YOD1 expression is involved in ccRCC progression and metastasis.

### YOD1 inhibits ccRCC cell proliferation and metastasis

**Fig. 2:**
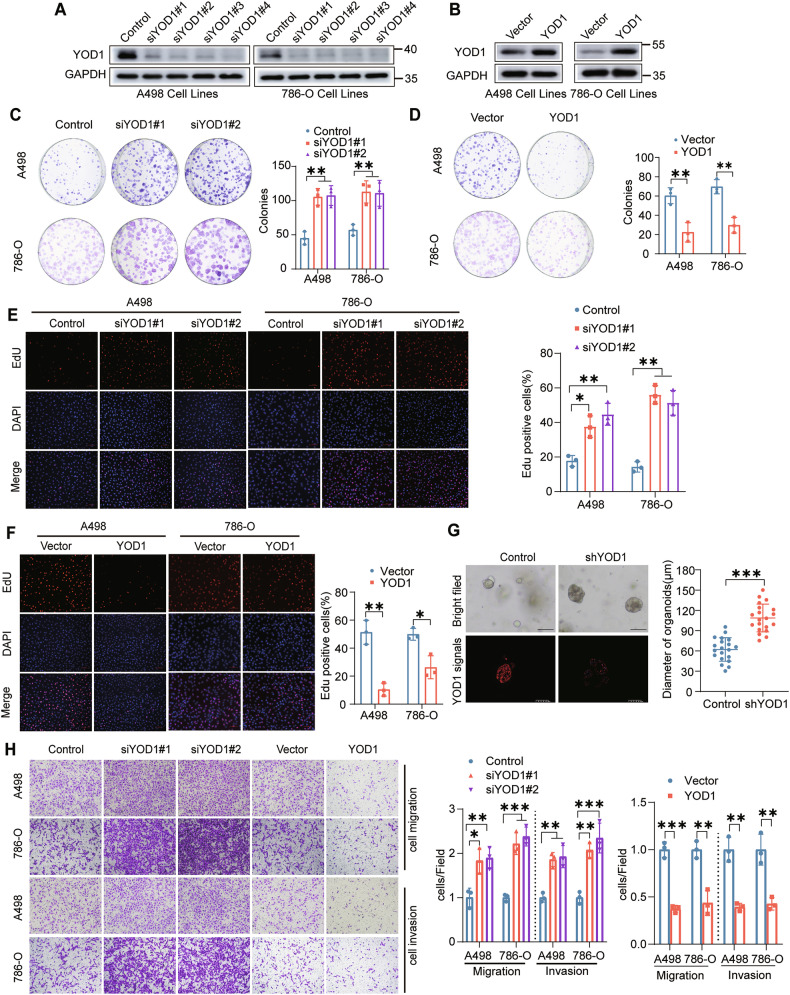
YOD1 inhibits the proliferation and metastasis of ccRCC cells in vitro. Efficacy of YOD1 siRNAs (**A**) and overexpression plasmids (**B**) in A498 and 786-O cells. **C**, **D** Changes in the clonogenic ability of ccRCC cells with either YOD1 knockdown or overexpression. **E**, **F** Representative EdU labeling images and corresponding quantification results for YOD1-knockdown or YOD1-overexpressing cells. Scale bar, 50 μm. **G** Knockdown of YOD1 affected the growth of patient-derived tumor organoids (PDOs). The morphology of these organoids is depicted, and their diameters were calculated. The expression of YOD1 in the two groups was examined via immunofluorescence. Scale bar, 100 μm. **H** The migration and invasion of ccRCC cells with either YOD1 knockdown or overexpression were investigated. Representative images and corresponding quantitative results are presented. The statistical analyses were performed with the ANOVA. *p < 0.05; **p < 0.01; ***p < 0.001.

To further investigate the cellular functions of YOD1 in ccRCC, we first silenced and overexpressed YOD1 via siYOD1 and YOD1 overexpression plasmids, respectively (Fig. 2A, B). The findings demonstrated that knocking down YOD1 significantly increased cell proliferation, whereas YOD1 overexpression resulted in a notable reduction in proliferation, as evaluated through colony formation assays (Fig. 2C, D) and EdU assays (Fig. 2E, F). Notably, YOD1 knockdown significantly promoted the proliferation of tumor organoids derived from ccRCC patients (Fig. 2G). Furthermore, YOD1 knockdown promoted cell migration and invasion, whereas YOD1 overexpression inhibited those processes (Fig. 2H). In conclusion, these data collectively indicate that reduced YOD1 expression is linked to increased aggressiveness of ccRCC, stimulating both cell proliferation and invasive properties in vitro.

### YOD1 interacts with ZNF24 directly

**Fig. 3:**
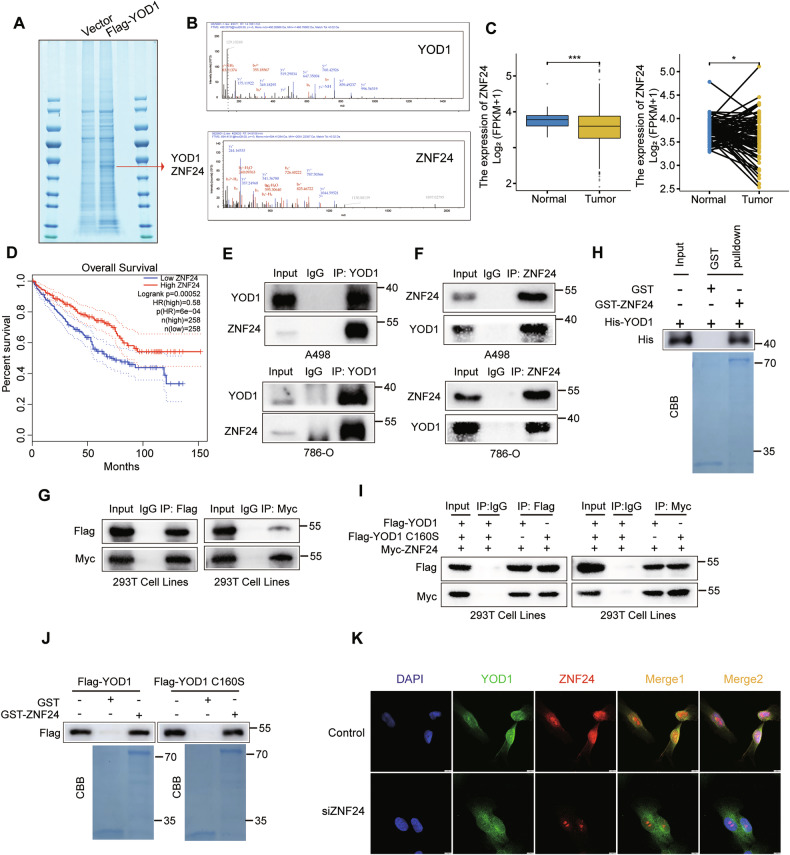
YOD1 directly interacts with ZNF24. **A** Proteins that interact with YOD1 were identified through co-IP and analyzed via mass spectrometry. **B** Unique peptides of YOD1 and ZNF24 were also identified via mass spectrometry assays. **C** ZNF24 mRNA levels were analyzed in ccRCC tissues compared with adjacent normal tissues, as well as in paired primary ccRCC tissues and their matched adjacent normal tissues, utilizing data from the TCGA database. **D** Kaplan–Meier plot illustrating overall survival based on the expression of ZNF24 in ccRCC patients was generated using data from the TCGA database. The interaction between YOD1 and ZNF24 was detected through an immunoprecipitation assay using an anti-YOD1 antibody (**E**), anti-ZNF24 antibody (**F**) or anti-Flag and anti-Myc antibodies (**G**). **H** Mixtures of purified HIS-YOD1 and GST-ZNF24 proteins were incubated with GSH-Sepharose beads. The beads were then stained with Coomassie Brilliant Blue (CBB). **I** Cells were cotransfected with Myc-ZNF24, Flag-YOD1 or Flag-YOD1 C160S plasmids. Lysates from these cells were subsequently immunoprecipitated with anti-Myc or anti-Flag antibodies. **J** Flag-YOD1 or Flag-YOD1 C160S plasmids were transfected into HEK-293T cells. The lysates were subsequently incubated with GST and GST-ZNF24 proteins coupled to GSH-Sepharose beads, followed by CBB staining. **K** Immunostaining for YOD1 (green) and ZNF24 (red) in 786-O cells. Nuclear 4′, 6-diamidino-2-phenylindole (DAPI; blue). Scale bar, 10 μm.

To investigate the mechanism of YOD1 in ccRCC development, we overexpressed YOD1 in A498 and 786-O cells and assessed various YOD1-regulated signaling pathways documented in prior studies [18, 20–25]. The result showed that only a slight elevation in Trim33 protein expression was detected (Supplementary Fig. 2B). Considering that YOD1 is a deubiquitinating enzyme, we transfected a Flag-YOD1 overexpression plasmid into 786-O cells. After performing tandem affinity purification, the YOD1-associated protein complex was isolated and analyzed via mass spectrometry to identify its substrate. ZNF24 and YOD1 were notably identified in the prey list (Fig. 3A, B). In addition, ZNF24 expression was downregulated in ccRCC, and low expression of ZNF24 was associated with higher T stages, metastasis, worse histological grades and clinical stages. (Fig. 3C, Supplementary Fig. 3A–E). Kaplan–Meier survival analysis revealed that low ZNF24 expression was correlated with significantly reduced OS and DFS in ccRCC (Fig. 3D, Supplementary Fig. 3F). Notably, ZNF24 is known to inhibit VEGFA mRNA transcription, which is vital for cell metastasis [26]. Thus, we hypothesized that YOD1 may affect ccRCC progression through the ZNF24/VEGF axis.

We then conducted reciprocal coimmunoprecipitation (co-IP) experiments, which corroborated the mass spectrometry findings, revealing an interaction of endogenous YOD1 and ZNF24 in cells (Fig. 3E, F). Additionally, ectopically derived Myc-tagged ZNF24 was identified in Flag-tagged YOD1 and viceversa (Fig. 3G). In addition, an in vitro Glutathione S-transferase (GST) pull-down assay revealed that the purified protein HIS-YOD1 directly bound to GST-ZNF24 but not to the GST control (Fig. 3H), thereby verifying a direct interaction between YOD1 and ZNF24. Notably, YOD1 enzymatic activity is not a prerequisite for binding to ZNF24 (Fig. 3I, J). Moreover, immunofluorescence staining demonstrated that endogenous YOD1 and ZNF24 were predominantly localized in ccRCC cells, while ZNF24 was knocked down as a negative control (Fig. 3K, Supplementary Fig. 4A, B). In conclusion, the presented data substantiate the hypothesis that ZNF24 is a genuine interacting partner of YOD1.

### Interaction of the SCAN box domain of ZNF24 with the UBX-like domain of YOD1

**Fig. 4:**
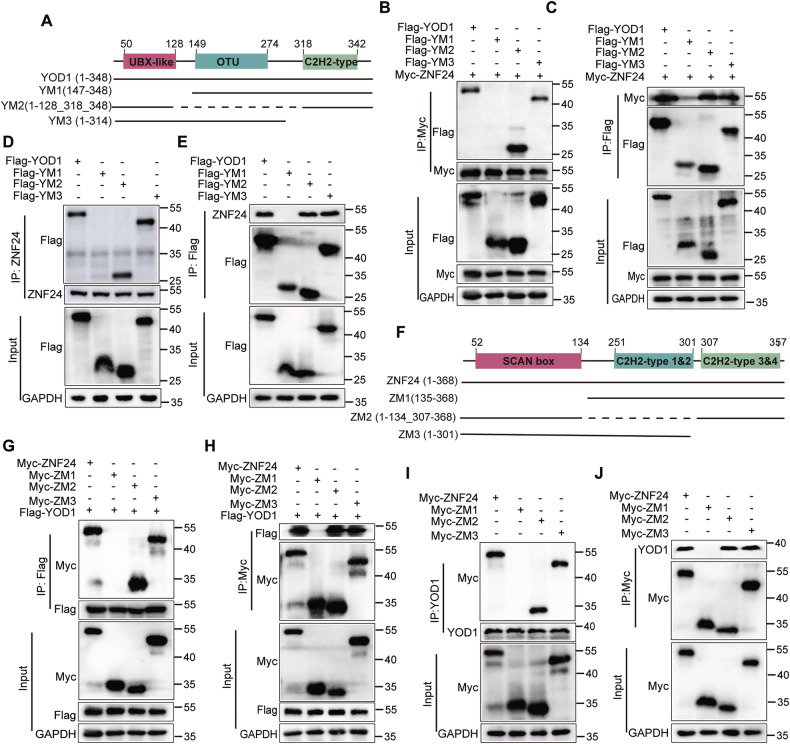
The SCAN box Domain of ZNF24 binds to the UBX-like domain of YOD1. **A** Domain structure of YOD1 and its deletion mutants (tagged with Flag) used in this study. **B**, **C** Myc-ZNF24 was cotransfected with YOD1 or its deletion mutants into HEK-293T cells. Cell lysates were analyzed by immunoprecipitation with antibodies against Myc (**B**) or Flag (**C**). Immunoblotting was performed with antibodies against Myc and Flag. YOD1 or its truncated mutants were overexpressed in HEK-293T cells. The cell lysates were then analyzed via immunoprecipitation with the indicated antibodies against ZNF24 (**D**) or Flag (**E**). **B**–**E** indicated that YOD1 binds to ZNF24 through its UBX-like domain. **F** The domain structure of ZNF24 and its deletion mutants (tagged with Myc) used in this study. **G**, **H** Flag-YOD1 was cotransfected with ZNF24 or its deletion constructs into HEK-293T cells. The cell lysates were then analyzed via immunoprecipitation with the indicated antibodies against Flag (**G**) or Myc (**H**). **I**, **J** ZNF24 or its three truncated constructs were overexpressed in HEK-293T cells and the lysates were then analyzed by immunoprecipitation with the indicated antibodies against YOD1 (**I**) or Myc (**J**). **G**–**J** indicated that ZNF24 binds to YOD1 through its SCAN box domain.

To gain further insight into the regions necessary for the binding of YOD1 to ZNF24, we generated truncations of YOD1 (Fig. 4A) and ZNF24 (Fig. 4F). Subsequently, full-length Flag-YOD1 and its various truncations, alone or combined with full-length Myc-ZNF24, were cotransfected into HEK293 cells. Coimmunoprecipitation experiments confirmed that both endogenous and exogenous ZNF24 bind to the UBX-like domain but not to the central otubain region or the Znf domain of YOD1 (Fig. 4B–E).

Furthermore, we expressed full-length Myc-ZNF24 and its truncations in HEK293 cells, either alone or in combination with full-length Flag-YOD1. Coimmunoprecipitation assays demonstrated that both endogenous and exogenous YOD1 interact with the N-terminal SCAN box region of ZNF24 (Fig. 4G–J). Collectively, these results provide evidence that the N-terminal SCAN-box domain of ZNF24 binds to the N-terminal UBX-like domain of YOD1.

### YOD1 deubiquitinates ZNF24 at the K308 site

**Fig. 5:**
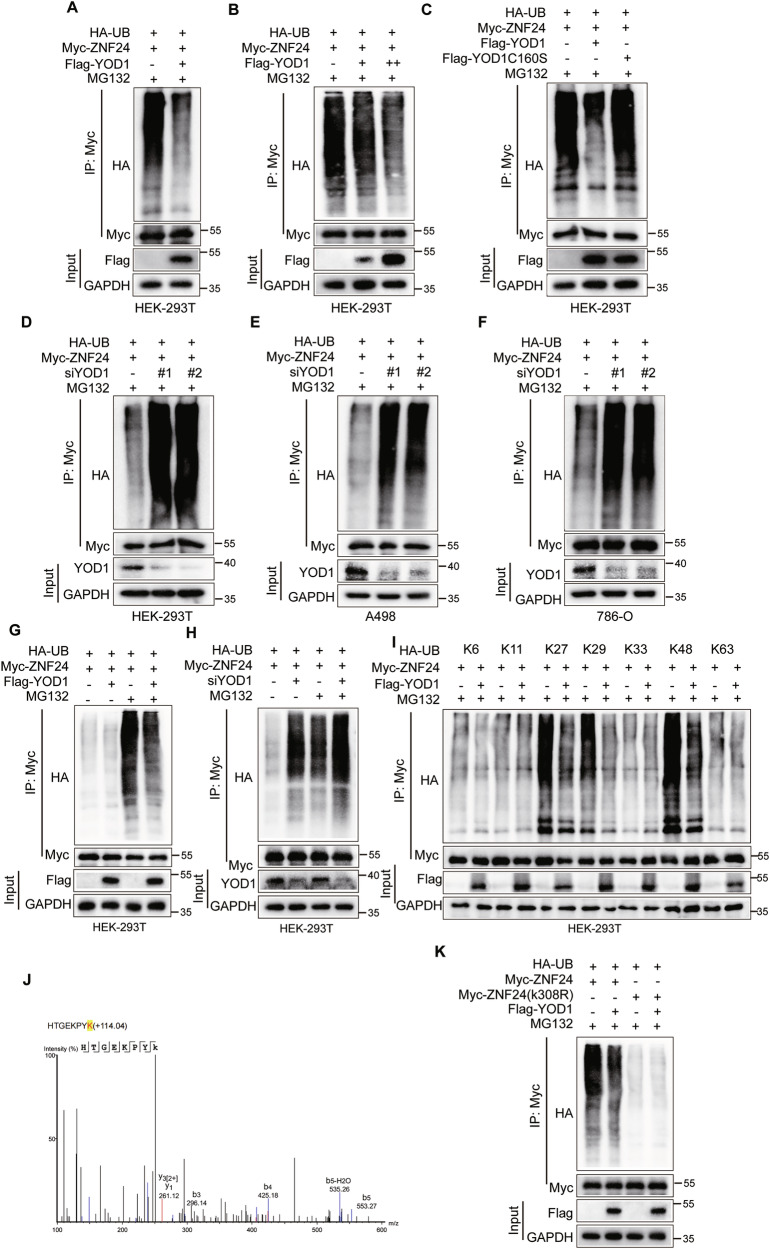
YOD1 deubiquitinates ZNF24 at the K308 site. **A** Myc-ZNF24 was cotransfected with HA-Ub and Flag-YOD1 into HEK-293T cells, and the level of ubiquitinated ZNF24 was detected via immunoprecipitation after treating the cells were treated with 25 μM MG132 for 6 h. **B** Increasing amounts of YOD1 plasmids (2 μg or 4 μg) were coexpressed with Myc-ZNF24 and HA-Ub in HEK-293T cells and the level of ubiquitinated ZNF24 was determined after treatment with MG132. **C** YOD1 or the enzymatically inactive mutant C160S was coexpressed with ubiquitin (UB) and ZNF24 in HEK-293T cells, and the level of ubiquitinated ZNF24 was detected. **D**–**F** Myc-ZNF24, HA-Ub, and YOD1 siRNAs were transfected into the indicated cells, and the level of ubiquitinated ZNF24 was subsequently analyzed. Myc-ZNF24, HA-Ub, and either the YOD1 overexpression plasmid (**G**) or YOD1 siRNA (**H**) were cotransfected into cells, with or without MG132 treatment, to measure the level of ubiquitinated ZNF24. **I** Cells were cotransfected with ZNF24, YOD1, and HA-Ub (K6, K11, K27, K29, K33, K48, and K63) to assess the deubiquitination of ZNF24 by YOD1. **J** Mass spectrometric analysis identified ZNF24 with ubiquitination at lysine residue 308. **K** Cells were cotransfected with Flag-YOD1, HA-Ub, and either ZNF24 or ZNF24-K308R plasmids to assess the deubiquitination of ZNF24 by YOD1.

Given that YOD1 is a component of the OTU family of DUBs, we investigated whether YOD1 deubiquitinates ZNF24. In HEK-293T cells overexpressing YOD1, the level of polyubiquitin chains on ZNF24 was markedly reduced (Fig. 5A). Moreover, ZNF24 polyubiquitination was dose-dependently suppressed by overexpression of wild-type YOD1 (Fig. 5B). Notably, the inhibition of ZNF24 polyubiquitination was observed with wild-type YOD1, whereas no such effect was observed with YOD1-C160S (Fig. 5C). These findings suggest that YOD1 deubiquitinates ZNF24 in a manner dependent on its enzymatic active site. Conversely, YOD1 knockdown significantly increased the level of ZNF24 polyubiquitination in both HEK-293T cells and ccRCC cell lines (A498 and 786-O) (Fig. 5D–F). Moreover, these discoveries were repeatedly verified irrespective of the presence or absence of MG132 (Fig. 5G, H), a highly efficacious inhibitory agent of the 26S proteasome that facilitates protein degradation subsequent to ubiquitination. Notably, MG132 significantly increased the polyubiquitination of ZNF24, indicating that the polyubiquitination of ZNF24 may be associated with protein degradation.

K48- and K63-linked ubiquitination are among the most extensively researched modifications of the ubiquitin protein. To determine the specific type of polyubiquitin chain that YOD1 cleaves from ZNF24, YOD1 was overexpressed, which significantly downregulated the K27-, K29-, and K48-linked ubiquitination of ZNF24, whereas other types of polyubiquitination remained unaffected (Fig. 5I). Mass spectrometry analysis revealed that ZNF24 was ubiquitinated at lysine 308 (Fig. 5J). Coimmunoprecipitation assays revealed a notable reduction in the ubiquitination of ZNF24 in the ZNF24-K308R variant relative to ZNF24-WT. Furthermore, YOD1 did not deubiquitinate the mutated ZNF24-K308R (Fig. 5K). In summary, YOD1 selectively removes K27-, K29-, and K48-linked polyubiquitination chains from the ZNF24 protein at the lysine 308 site.

### YOD1 maintains ZNF24 stability

**Fig. 6:**
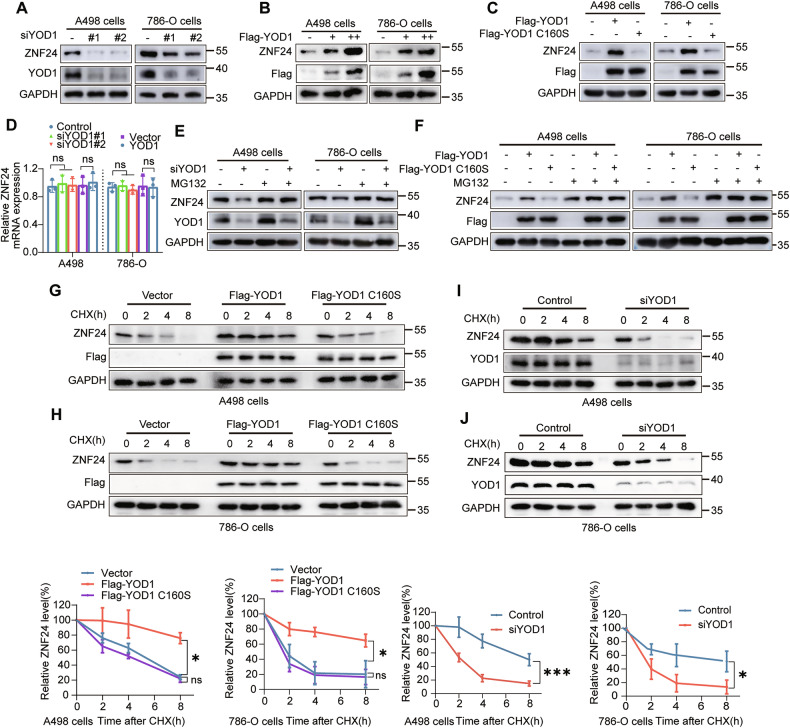
YOD1 maintains ZNF24 stability. **A** Western blotting analysis of YOD1 and ZNF24 was performed in cells transfected with YOD1 siRNAs. **B** Western blotting analyses of Flag-YOD1 and ZNF24 protein levels in cells transfected with increasing amounts of Flag-YOD1 (2 μg and 4 μg). **C** Detection of ZNF24 protein levels in cells transfected with either YOD1 or YOD1 C160S plasmids. **D** Relative ZNF24 mRNA levels in cells transfected with YOD1 siRNAs or Flag-YOD1. (Student’s *t*-test) **E**, **F** Cells were transfected with YOD1 siRNAs (**E**), Flag-YOD1 or Flag-YOD1 C160S (**F**) and treated with MG132 for 6 h. The protein levels of ZNF24 were then detected. Flag-YOD1 or Flag-YOD1 C160S was transfected into A498 (**G**) and 786-O (**H**) cells, followed by treatment with 20 μM CHX. The cells were harvested at designated time points to assess ZNF24 degradation. **I**, **J** Detection of ZNF24 degradation after YOD1 was knocked down. Lower panel: Quantification of changes in ZNF24 protein levels. The statistical analyses were performed with the ANOVA. *p < 0.05; ***p < 0.001.

As YOD1 selectively removes K27-, K29-, and K48-linked polyubiquitination chains from ZNF24, we next investigated its regulatory effects on ZNF24. As shown in Fig. 6A, suppression of YOD1 levels resulted in decreased ZNF24 protein expression. Conversely, YOD1 overexpression significantly upregulated ZNF24 levels in a dose-dependent manner (Fig. 6B). Notably, only the exogenous overexpression of wild type YOD1 (but not the catalytic inactivated YOD1 mutant C160S) increased ZNF24 levels (Fig. 6C). Nevertheless, qRT‒PCR data demonstrated that neither silencing nor overexpression of YOD1 affected ZNF24 mRNA expression (Fig. 6D). These findings suggest that YOD1 regulates ZNF24 expression at the posttranscriptional level. Additionally, the destabilization of the ZNF24 protein induced by YOD1 suppression was shown to be dependent on proteasome and was abolished following treatment with MG132, a proteasome inhibitor (Fig. 6E, F). Notably, in protein half-life experiments, overexpression of wild-type YOD1 significantly slowed the degradation of ZNF24 compared to the control group, whereas ZNF24 degradation in the YOD1-C160S overexpression group was similar to that of the control (Fig. 6G, H). In contrast, ZNF24 degradation was accelerated following YOD1 knockdown (Fig. 6I, J). These findings indicate that YOD1 tightly controls ZNF24 protein stability. In conclusion, YOD1 regulates ZNF24 protein stability in a proteasome regulated mode.

### YOD1 inhibits ccRCC progression through ZNF24/VEGFA axis

**Fig. 7:**
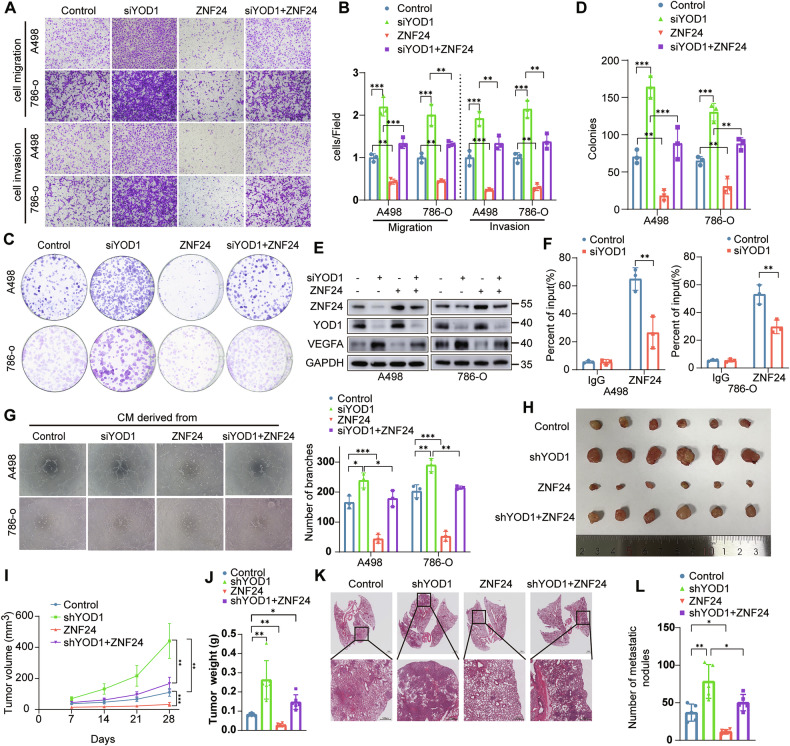
YOD1 inhibits ccRCC progression through the ZNF24/VEGFA axis. **A**–**D** Cells were transfected with YOD1 siRNAs or ZNF24 plasmids (with or without cotransfection of YOD1 siRNAs), followed by Transwell migration and invasion assays (**A**, **B**) and colony formation assays (**C**, **D**). **E** ZNF24, YOD1, and VEGFA protein levels in cells, as described in (**A**), were detected by western blot analysis. **F** Lysates from cells transfected with YOD1 siRNA were subjected to ChIP assays with an anti-ZNF24 antibody. **G** The tube formation ability of HUVECs treated with conditioned medium (CM) from the indicated cells was evaluated. **H** Representative images of xenograft tumors from control, shYOD1, ZNF24 and shYOD1 + ZNF24 nude mice. **I**, **J** Comparison of xenograft tumor volume and weight between groups (*n* = 6). **K**, **L** HE staining and the quantification of lung nodes in NCG mice are shown. Scale bar, 1 mm for the upper panel and 500 μm for the lower panel. The statistical analyses were performed with the ANOVA. *p < 0.05; **p < 0.01; ***p < 0.001.

Research has demonstrated that ZNF24 functions as a transcriptional inhibitor in various cancers by repressing VEGFA transcription. To investigate whether low YOD1 expression promotes ccRCC progression and metastasis via the ZNF24/VEGFA axis, we first cotransfected siYOD1 or Myc-ZNF24 plasmids into A498 and 786-O cells. The overexpression of ZNF24 in YOD1-knockdown cells significantly reversed the positive effects of YOD1 on colony formation, migration, and invasion (Fig. 7A–D). Furthermore, YOD1 knockdown resulted in significant upregulation of VEGFA expression, whereas ZNF24 overexpression reversed this effect (Fig. 7E). A ChIP assay confirmed that ZNF24 binds to the VEGFA promoter and that YOD1 knockdown reduced the interaction between ZNF24 and the VEGFA promoter (Fig. 7F). Additionally, the tube formation ability of HUVECs was enhanced by medium collected from YOD1-knockdown A498 and 786-O cells, but this increase was negated by ZNF24 overexpression (Fig. 7G). Knockdown of YOD1 markedly promoted subcutaneous tumor growth (Fig. 7H–J) and lung metastasis (Fig. 7K, L), and these effects were reversed by ZNF24 overexpression. We cotransfected siYOD1 and siVEGFA into A498 and 786-O cells. Knockdown of VEGFA in YOD1 knockdown cells significantly reversed the promotion of knockdown of YOD1 on colony formation, migration and invasion (Supplementary Fig. 5A–E). Overall, we conclude that YOD1 acts as a tumor suppression factor by attenuating tumor growth and metastasis through the ZNF24/VEGFA pathway in ccRCC.

### YOD1 shows positive correlation with ZNF24 in clinical ccRCC specimen and associated with survival

**Fig. 8:**
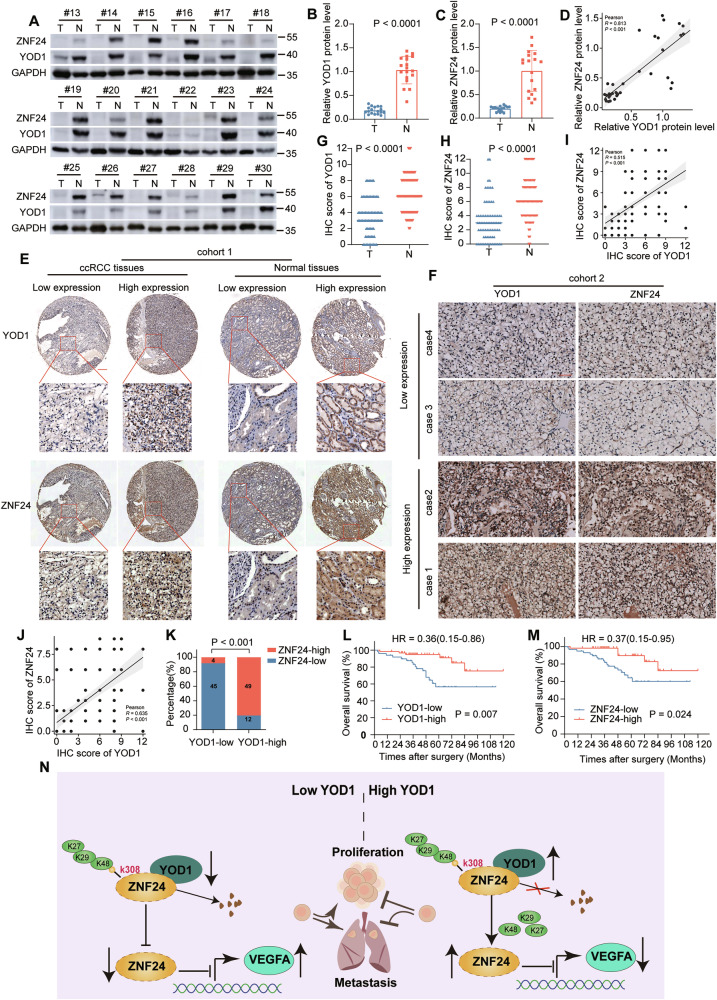
YOD1 is positively correlated with ZNF24 in clinical ccRCC samples. **A**–**D** The correlation between YOD1 and ZNF24 levels in ccRCC tissues was analyzed by immunoblotting. (Student’s t-test) **E**, **F** Representative images of YOD1 and ZNF24 expression in ccRCC tissues from two cohorts (cohort 1: 80 paired tumor and normal tissue samples, cohort 2: 110 surgical tumor samples) were obtained via IHC staining. Scale bar, 200 μm for cohort 1 and 50 μm for cohort 2. **G**, **H** Quantification of YOD1 and ZNF24 levels in cohort 1. (Student’s t-test) **I** Pearson’s correlation between YOD1 and ZNF24 expression in cohort 1. **J**, **K** Correlation between YOD1 and ZNF24 levels in cohort 2. (Chi-square tests) **L**, **M** Prognostic values of YOD1 and ZNF24 in ccRCC cohort 2. **N** Schematic diagram of the YOD1/ZNF24/VEGFA axis in regulating the growth and metastasis of ccRCC. (Log-rank test).

Western blotting was used to determine the expression of YOD1 and ZNF24 in ccRCC tissues. Results revealed that low YOD1 protein levels were associated with decreased ZNF24 expression in nearly all ccRCC samples compared with normal tissues (Pearson *R* = 0.813, *p* < 0.001) (Fig. 8A–D). To further validate these findings, we performed immunohistochemical staining for YOD1 and ZNF24 in a tumor microarray for ccRCC (cohort 1) (Fig. 8E), which consisted of 80 paired tumor and normal tissue samples and an additional 110 surgical tumor samples (cohort 2) (Fig. 8F). Both YOD1 and ZNF24 exhibited low expression in the majority of the tumor specimens (Fig. 8G, H). Lower YOD1 levels were associated with lower ZNF24 levels in both cohort 1 (Pearson *R* = 0.515, *p* < 0.001) (Fig. 8I) and cohort 2 (Pearson *R* = 0.635, *p* < 0.001) (Fig. 8J, K). We also investigated the implications of this relationship for survival outcomes among ccRCC patients in cohort 2. The findings revealed that patients with low YOD1 expression had worse outcomes than those with high YOD1 expression did, a trend also observed for ZNF24 protein expression, with improved prognostic value after combining YOD1 and ZNF24 low and high expression data into one dataset (Fig. 8L, M, Supplementary Fig. 5F). Collectively, these findings indicate that the downregulation of YOD1 is correlated with ZNF24 expression and poor survival outcomes.

## Discussion

Currently, treatment options for metastatic ccRCC are limited, with targeted therapies such as sunitinib being common [4, 27, 28]. However, these approaches often yield only temporary and limited effects, highlighting the urgent research needed to understand the underlying molecular mechanisms of ccRCC metastasis [5]. The proteasome system is essential for regulating multiple facets of tumor biology, including cell proliferation and metastasis [29, 30]. Recent investigations into the OTU family of deubiquitinating enzymes have gained prominence in cancer research, revealing new insights into the mechanisms of tumor metastasis [31–33]. Nevertheless, research in this field pertaining to ccRCC remains scarce. Thus, to identify OTUs involved in regulating ccRCC cell metastasis, we utilized siRNAs to knock down 14 human OTU DUBs and conducted Transwell migration assays. The results demonstrated that YOD1 significantly influenced the migration capacity of ccRCC cells. Moreover, YOD1 knockdown promoted tumor growth and metastasis in vitro and in vivo. Notably, analysis of data from the TCGA database indicated that low YOD1 levels were significantly correlated with reduced survival in ccRCC patients. Thus, we propose that YOD1 is an important tumor suppressor worthy of further investigation in ccRCC.

YOD1, also referred to as OTUD2, exhibits substantial enzymatic catalytic activity that can modulate cellular signaling, consequently impacting various cellular functions [23, 33–35]. Interestingly, YOD1 may regulate distinct target genes in various tumors, thereby playing a role in either promoting or inhibiting tumor development. YOD1 regulates the stability of PML/RARα, CDK1, and ITCH, promoting malignant phenotypes in acute promyelocytic leukemia, triple-negative breast cancer, and hepatocellular carcinoma, respectively [18, 24, 36]. Additionally, YOD1 correlates with poor patient outcomes in gallbladder cancer, osteosarcoma, and pancreatic cancer [19, 37, 38]. Conversely, increased YOD1 expression blocks ERK signaling by stabilizing TRIM33 activation, thereby inhibiting tumor invasion and lymph node metastasis in squamous cell carcinoma of the head and neck [25]. Furthermore, YOD1 reverses the proliferative effects of upregulated miR-373 in cervical cancer cells [39]. These results may stem from tumor heterogeneity.

In this study, for the first time, we identified the tumor-suppressive role of YOD1 in ccRCC. Notably, we constructed patient-derived ccRCC organoids and demonstrated that YOD1 silencing significantly accelerated organoid growth, further underscoring the critical tumor-suppressive function of YOD1 in ccRCC. In the future, we plan to investigate whether YOD1 regulates the growth of organoids derived from metastatic ccRCC patients in an enzyme activity-dependent manner. Additionally, we intend to explore the mechanisms of metastasis and drug resistance in ccRCC at the organoid level.

ZNF24, an important transcriptional repressor of VEGFA, is frequently deleted in various tumors [40, 41]. Additionally, ZNF24 has also been shown to inhibit cell proliferation in cancers [42–44]. Notably, it is recognized as a key regulator of angiogenesis [40]. Nevertheless, the role of ZNF24 in ccRCC has yet to be investigated. Here, our results demonstrated that ZNF24 expression was markedly downregulated in ccRCC tissues and was significantly correlated with YOD1 expression. Furthermore, ZNF24 overexpressing reversed the growth, metastasis and angiogenesis of ccRCC resulted by YOD1 knockdown. Earlier work has indicated that several molecules are responsible for regulating ZNF24 transcription [45, 46]. However, reports on the posttranslational modifications of ZNF24, particularly regarding deubiquitination, are notably lacking. Our study demonstrated for the first time that YOD1 interacted with and stabilized ZNF24, with the UBX-like domain of YOD1 binding to the SCAN box domain of ZNF24. YOD1 specifically removes ubiquitination from ZNF24 at the K308 site, preventing its degradation by the proteasome and thereby increasing its stability. Notably, the deubiquitination of ZNF24 by YOD1 was dependent on its OTU enzyme active site, as the YOD1-C160S mutant did not deubiquitinate ZNF24. Additionally, we identified ZNF24 as a critical mediator of YOD1 function both in vitro and in vivo.

Angiogenesis is a prominent feature of renal cancer, with VEGFA recognized as a pivotal signaling molecule in regulating this process [40, 47]. Aberrant activation of VEGFA signaling, often due to mutations in the VHL gene, is prevalent in RCC [48]. Consequently, most targeted therapies for ccRCC focus on VEGF or its receptor [49]. However, drug resistance remains a significant challenge, highlighting the urgent need to identify new regulatory factors for VEGFA. ZNF24, a crucial transcriptional repressor of VEGFA, binds to an 11 bp fragment in the proximal promoter region on the VEGFA gene, inhibiting its transcription and subsequently impeding tumor growth and invasion [40]. Our findings indicated that YOD1 knockdown in ccRCC cells decreased the protein stability of ZNF24, leading to attenuated transcriptional repression of VEGFA, which resulted in increased VEGFA expression and promoted angiogenesis. In contrast, ZNF24 overexpression partially reversed the YOD1-mediated effects on angiogenesis, suggesting that there may be a possibility that YOD1 may regulate this process by inhibiting VEGFA expression in an alternative ZNF24-independent manner. Further investigations are recommended to explore additional molecular mechanisms that YOD1 influences VEGFA-mediated ccRCC progression.

Nevertheless, this study has several limitations. We established a lung metastasis model by directly injecting YOD1 knockout and control cells into the tail vein of NCG mice, an approach widely reported in the literature [50–52]. This method bypasses primary tumor formation and directly assesses the likelihood of tumor cell metastasis to the lungs. Although effective for evaluating metastasis, it has some limitations. Specifically, it does not account for the complexities associated with primary tumor establishment and its potential impact on metastatic behavior. In contrast, the DOX-induced knockout model, which involves establishing a primary tumor followed by the induction of knockouts, offers a more scientifically rigorous approach to studying tumor metastasis [53, 54]. This model more accurately simulates tumor metastasis in vivo. It allows for the intervention of specific genes after primary tumor formation to explore their effects on tumor metastasis, thereby strengthening the validity of the conclusions. In the future, we plan to refine the construction method of the ccRCC lung metastasis model.

## Conclusion

Our present work presents the first demonstration of posttranslational deubiquitination of ZNF24, demonstrating that YOD1 regulates the ZNF24/VEGFA axis in an enzyme activity-dependent manner. YOD1 exhibits tumor-suppressive effects by inhibiting the growth and metastasis of ccRCC (Fig. 8N). Moreover, YOD1 downregulation is significantly correlated with poor prognosis in ccRCC patients. Collectively, the YOD1/ZNF24/VEGFA axis plays a pivotal role in the progression of ccRCC.

## Materials and methods

### Clinical samples and tissue microarray

Primary clear cell renal carcinoma (ccRCC) tissues and their corresponding adjacent noncancerous tissues (NCTs) were obtained from the First Affiliated Hospital of Nanchang University. The study was conducted in accordance with approved guidelines and received ethical clearance from the Clinical Research Ethics Committee of the First Affiliated Hospital of Nanchang University. Additionally, a human ccRCC tissue microarray, labeled ZL-KIC1601, was acquired from Shanghai Zhuoli Biotechnology Co., Ltd, consisting of 80 pairs of adjacent normal and tumor tissues.

### Cell culture

The ACHN, A498, and HK-2 cell lines were cultured in Minimum Essential Medium (MEM) from Pricella, Wuhan, while the 769-P and 786-O cell lines were maintained in RPMI-1640 medium from Gibco, CA, USA. The HEK-293T cells were grown in Dulbecco’s Modified Eagle Medium (DMEM), and the Caki-1 cells in McCoy’s 5 A medium, also sourced from Pricella, Wuhan. All cultures were supplemented with 10% fetal bovine serum and 1% penicillin/streptomycin, both from Gibco, CA, USA, and maintained at 37 °C in an atmosphere containing 5% CO_2_.

### Antibodies and purified proteins

The antibodies used were as follows: anti-YOD1 (Proteintech, 25370-1-AP), anti-ZNF24 (Proteintech, 11219-1-AP; Santa Cruz, sc-393359; Abcam, ab180121), anti-VEGFA (Proteintech, 26157-1-AP; Proteintech, 19003-1-AP), anti-HA (Cell Signaling Technology, 3724S), anti-Flag (Proteintech, 20543-1-AP; Proteintech, 66008-4-Ig), anti-Myc (Cell Signaling Technology, 2276S; Proteintech, 16286-1-AP), anti-GAPDH (Proteintech, HRP-60004), and anti-His (Proteintech, 66005-1-Ig), anti-CDK1 (Proteintech, 67575-1-Ig), anti-TGF-β3 (Proteintech, 18942-1-AP), anti-Trim33 (Proteintech, 55374-1-AP), anti-YAP (Cell Signaling Technology, 4912S), anti-NF-κB p65 (Cell Signaling Technology, 8242S), anti- Phospho-NF-κB p65 (Cell Signaling Technology, 3033T), anti-β-Catenin (Cell Signaling Technology, 37447T), anti-Phospho-β-Catenin (Cell Signaling Technology, 9561T), anti-P53 (Santa Cruz, sc-393031). The purified proteins were as follows: GST-ZNF24 protein (Proteintech, Ag1724) and His-YOD1 protein (Abcam, ab269141).

### Glutathione S-transferase (GST) pull-down assays

Two micrograms of recombinant GST or GST-ZNF24 protein was combined with 2 μg of recombinant His-YOD1 protein in 1 mL of IP binding buffer and incubated for 4 h. Subsequently, 30 μl of glutathione magnetic beads (Yeasen, Shanghai) were washed three times with IP washing buffer, the beads were added to the protein mixture, and the mixture was incubated for an additional 2 h. After incubation, a magnetic stand was used to facilitate separation of the beads. Following three washes, the supernatant was discarded. The bound proteins were eluted with protein loading buffer at 100 °C, and Western blot analysis was subsequently performed.

### Mass spectrometry analysis

The initial procedures involved lysing the cells using IP lysis buffer from Beyotime, followed by overnight incubation of the total proteins with Flag-labelled magnetic beads at 4 °C. Subsequently, the proteins were washed with PBST and separated by SDS-PAGE, before being stained with Coomassie Brilliant Blue or subjected to Western blotting. The bands of differentially expressed proteins were excised and sent to Weinafi Biotechnology (Shenzhen, China) for liquid chromatography-tandem mass spectrometry (LC-MS/MS) analysis.

### In vivo deubiquitination assay

In this experiment, HEK293T cells were cotransfected with plasmids encoding HA-Ub, Myc-ZNF24, Flag-YOD1, or Flag-YOD1 C160S using PEI 40 K Transfection Reagent (Servicebio, Wuhan). Following a 48-h incubation period, the cells were treated with 25 μM MG132 for a period of six hours. Ubiquitinated ZNF24 was then immunoprecipitated using Myc-tagged magnetic beads and analyzed by Western blotting.

### Co-immunoprecipitation (Co-IP) analysis

Co-IP assays were conducted according to the operating manual. A mixture of 500 µL of cell lysate, 30 µL of Protein A/G magnetic beads (MedChemExpress, New Jersey, USA), and 2 µg of primary antibody was incubated at 4 °C for 2 h. Subsequently, the mixture was placed on a magnetic stand to separate the supernatant, which was then discarded. The precipitated mixture of antibodies and magnetic beads was incubated with the protein solution at 4 °C overnight. Finally, the mixture was placed on a magnetic stand, washed with PBST, boiled, and eluted with 2.5× protein loading buffer for 10 min. The target protein was then detected by Western blotting.

### Chromatin immunoprecipitation (ChIP)

The EZ-ChIPTM chromatin immunoprecipitation kit (Millipore, Massachusetts, USA) was used for the ChIP assay and the process was performed in accordance with the manufacturer’s protocol. The following antibodies were used: mouse anti-ZNF24 (Santa Cruz, sc-393359) and mouse IgG (Proteintech, B900620.) Pooled DNA fragments were amplified by qRT-PCR.

### Subcutaneous xenograft and lung metastasis models

In the mouse xenograft tumour assay in Fig. 1J–L, each group consisted of 6 four-week-old male BALB/c nude mice (GemPharmatech Co., Ltd. Nanjing, China), control and shYOD1#1 or shYOD1#2 786-O cells (6 × 10^6^ each) were mixed with Matrigel (1:1) and then injected subcutaneously into the axillae of the mice. Tumor size was measured weekly with calipers, and tumor volume was calculated using the following formula: V = length × width^2^ × 0.5. Experimentally identical methods in Fig. 7H–J. In the lung metastasis models of Figs. 1M, N and 7K, L, 5 NCG (NOD-Prkdc^scid IL2rg^null) mice from GemPharmatech Ltd. were used in each group. Based on preliminary experimental results, mice were injected with cells via the tail vein, euthanised and tissues harvested after 4 weeks. All animal experiments were approved by the Ethics Committee of Laboratory Animals at the First Affiliated Hospital of Nanchang University, ensuring that the study adhered to ethical guidelines and maintained animal welfare standards.

### Statistical analysis

Statistical analysis was conducted with Prism 8.0 (GraphPad, USA). Statistical analyses were conducted using the Student’s *t*-test, one-way or two-way ANOVA. Bonferroni correction was applied for multiple comparisons. Across all data sets, *p* values below 0.05 were deemed statistically significant.

Additional materials and methods are presented in the Supplementary information.

## Supplementary information

supplementary information

Original Data File

aj-checklist

## Data Availability

All data generated or analyzed during this study are included in this published article and its Supplementary Information files.
