# Supplementary material for: The deubiquitinase YOD1 suppresses tumor progression by stabilizing ZNF24 in clear cell renal carcinoma
Source: Cell Death Dis. 2025 Apr 24;16(1):334. doi: 10.1038/s41419-025-07673-2 (PMC12022293; doi:10.1038/s41419-025-07673-2)
Supplement: Supplementary file 1 — supplementary information [file 41419_2025_7673_MOESM1_ESM.pdf]

Supplementary materials

The deubiquitinase YOD1 suppresses tumor progression by stabilizing ZNF24 in clear cell renal carcinoma

Ji Liu [1,2,6], Ying Lu [3,6], Runye Zhu [1,2], Ping Xi [4], Zhihao Yang [1,2], Zhipeng Zhang [1,2], Yunbing Xiong [1,2], Yifu Liu [5], Qiqi Zhu [1,2], Ting Sun [1,2,#], Wenjie Xie [1,2,#], Binbin Gong [1,2,#]

[1]Department of Urology, The First Affiliated Hospital, Jiangxi Medical College, Nanchang University, Nanchang 330000, Jiangxi, China.
[2]Jiangxi Provincial Key Laboratory of Urinary System Diseases, Nanchang City, Jiangxi Province 330000, China.
[3]Department of Clinical Laboratory, The Second Affiliated Hospital, Jiangxi Medical College, Nanchang University, Nanchang 330000, Jiangxi, China.
[4]Department of Thoracic Surgery, XinSteel Center Hospital, Xinyu, China.
[5]The Second Affiliated Hospital, Department of Urology, Hengyang Medical School, University of South China, Hengyang, Hunan, China.
[6]These authors contributed equally to this study: Ji Liu, Ying Lu.
[#]Corresponding author: Binbin Gong, E-mail: ndyfy05944@ncu.edu.cn, Wenjie Xie, E-mail: ndyfy04140@ncu.edu.cn and Ting Sun, E-mail: 361439919033@email.ncu.edu.cn.

Supplementary Figures, Supplementary Tables, Supplementary Methods

Supplementary Figures

Supplementary Figure 1

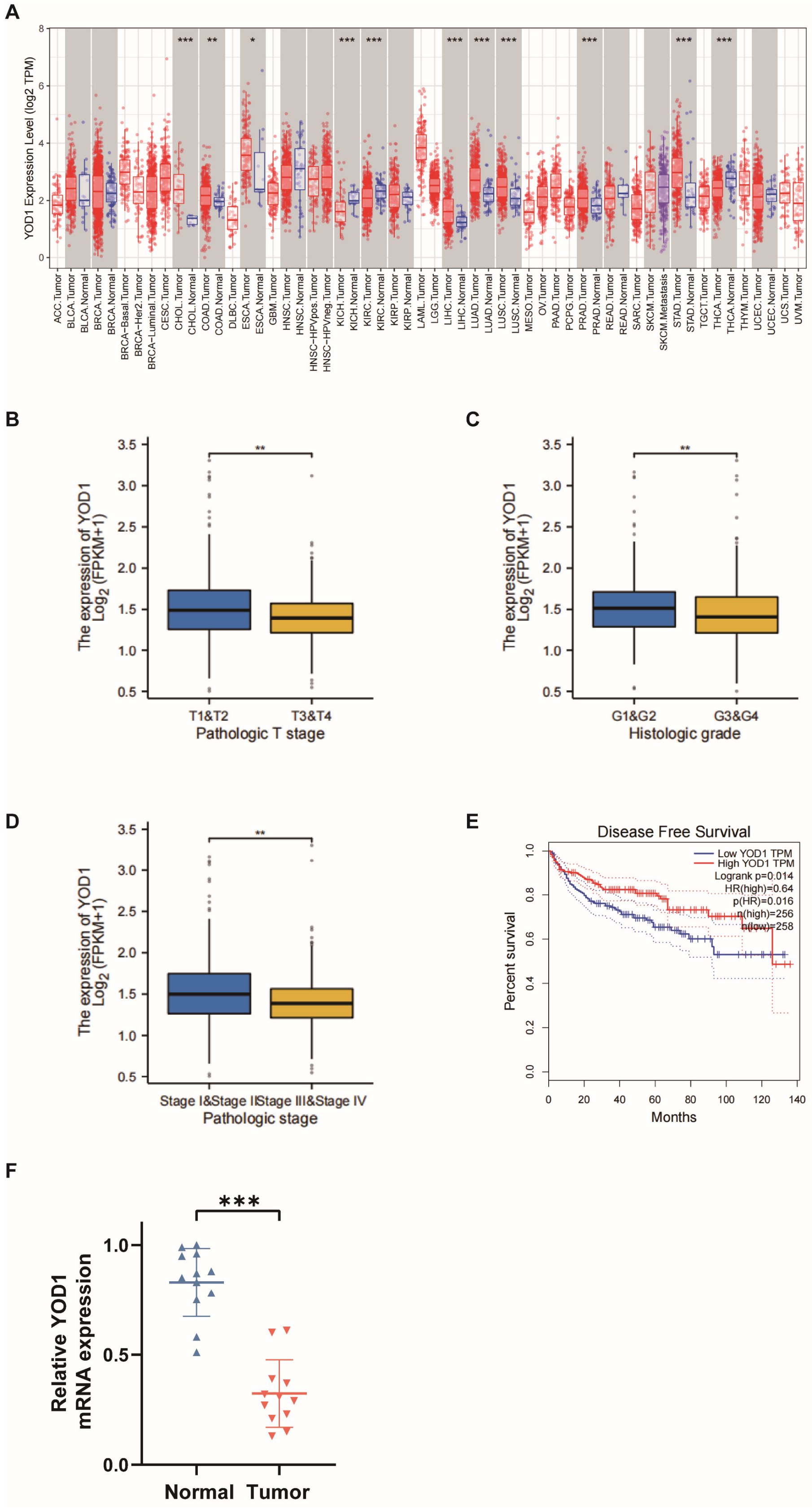

Fig S1. YOD1 expression profile in pan-cancer and its correlation with clinical features and prognosis of ccRCC. **A** YOD1 expression profile in pan-cancer. **B-D** Differential YOD1 expression across pathological T-stages **(B)**, histologic grades **(C)** and pathologic stages **(D)** in ccRCC patients. **E** Kaplan-Meier curve representing the association of YOD1 expression levels with disease-free survival in ccRCC patients (data from TCGA). All these data were obtained from the TCGA database. $*p < 0.05$; $**p < 0.01$. $***p < 0.001$. **F** Differences in YOD1 mRNA levels between ccRCC tissues and corresponding normal tissues were analyzed via real-time quantitative PCR.

Supplementary Figure 2

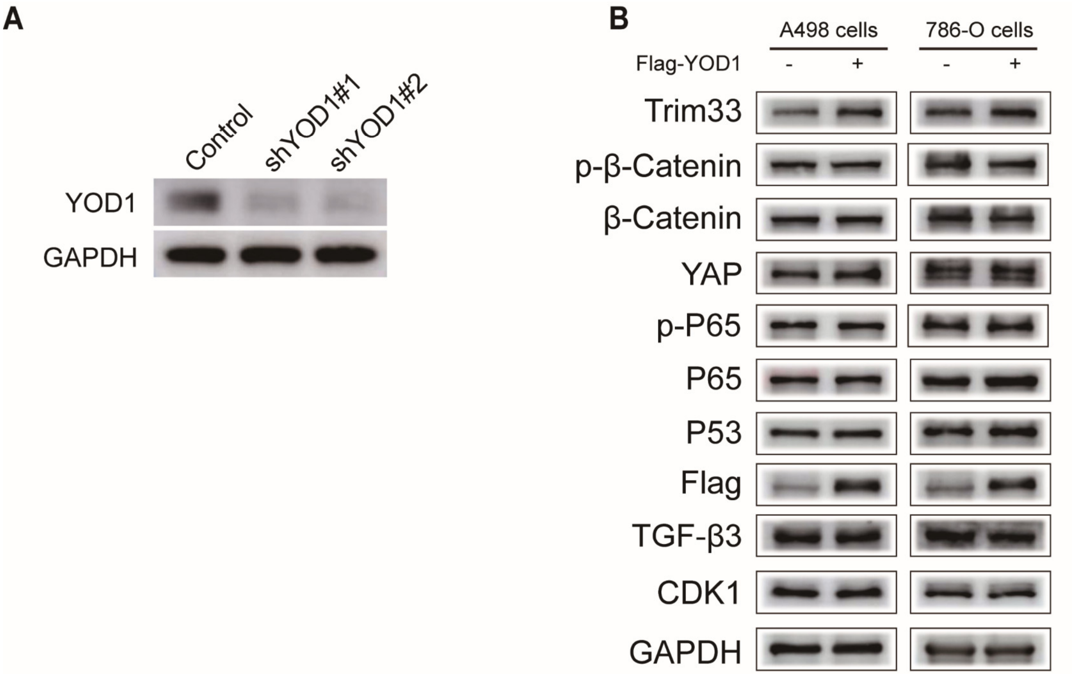

Fig S2. Construction of a cell line with stable YOD1 silencing and detection of previously reported signaling pathways regulated by YOD1. A Western blot showing stable knockdown of YOD1 in 786-O cells. **B** Detection of protein abundance of Trim33, β-catenin, YAP, P65, P53, TGF-β3, CDK1 after overexpression of YOD1 in A498 and 786-O cells.

Supplementary Figure 3

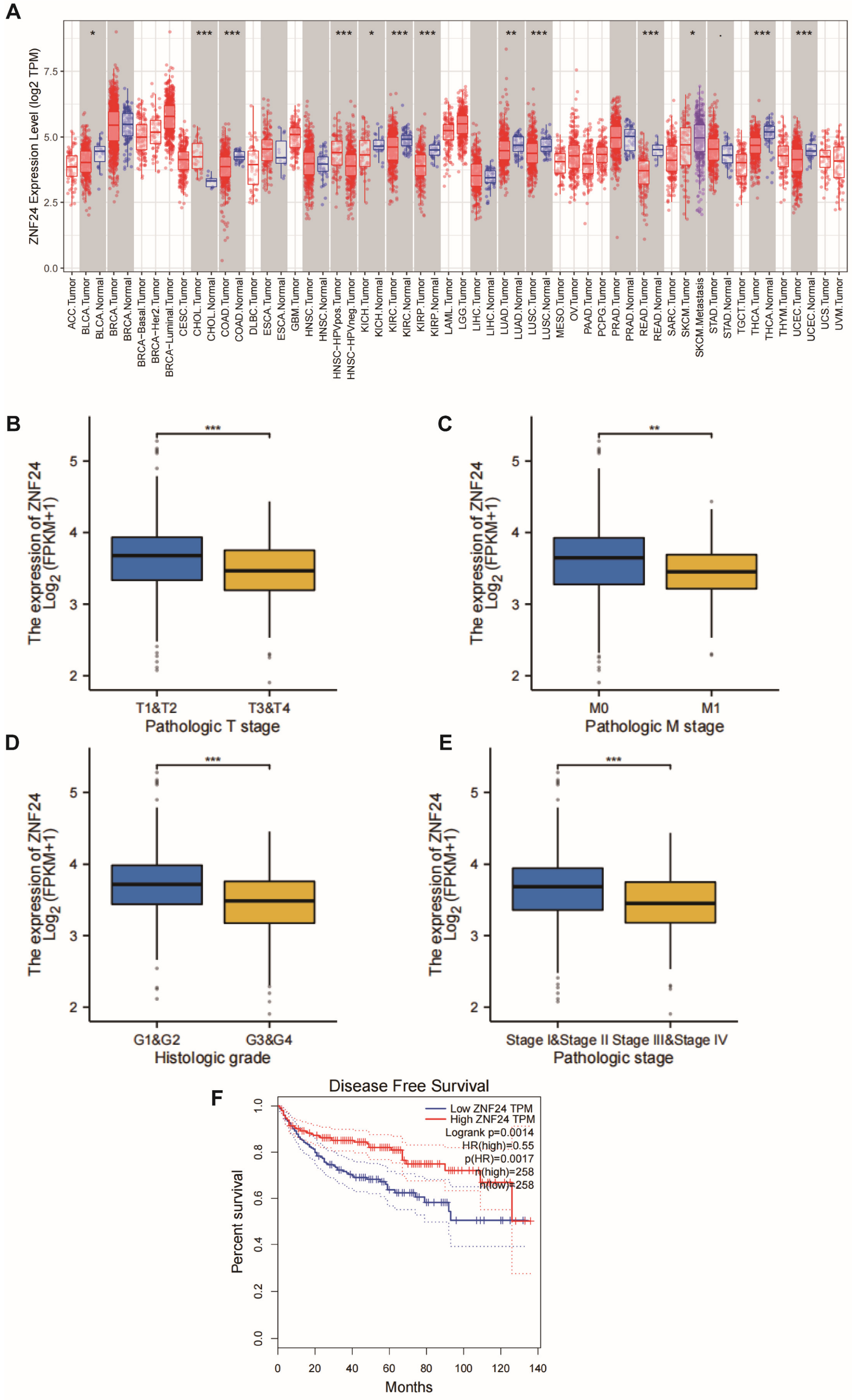

Fig S3. ZNF24 expression profile in pan-cancer and its correlation with clinical features and prognosis of ccRCC. **A** ZNF24 expression profile in pan-cancer. **B-E** Differential ZNF24 expression across pathological T-stages **(B)**, pathologic M stages **(C)**, histologic grades **(D)** and pathologic stages **(E)** in ccRCC patients. **F** Kaplan-Meier curve representing the association of ZNF24 expression levels with disease-free survival in ccRCC patients. All these data were obtained from the TCGA database.

Supplementary Figure 4

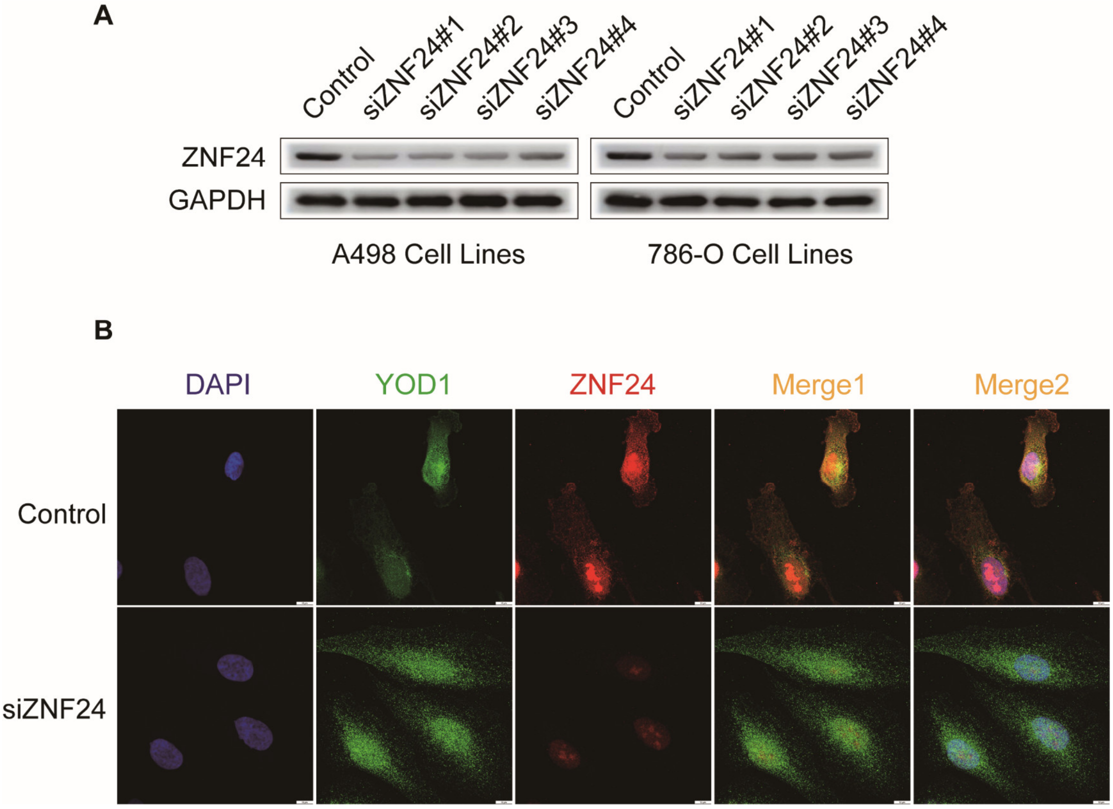

Fig S4. Knockdown of ZNF24 as a negative control in immunofluorescence confocal assays. **A** Efficacy of ZNF24 siRNAs. **B** Immunostaining for YOD1 (green) and ZNF24 (red) in A498 cells. Nuclear 4′, 6-diamidino-2-phenylindole (DAPI; blue). Scale bar, 10 μm.

Supplementary Figure 5

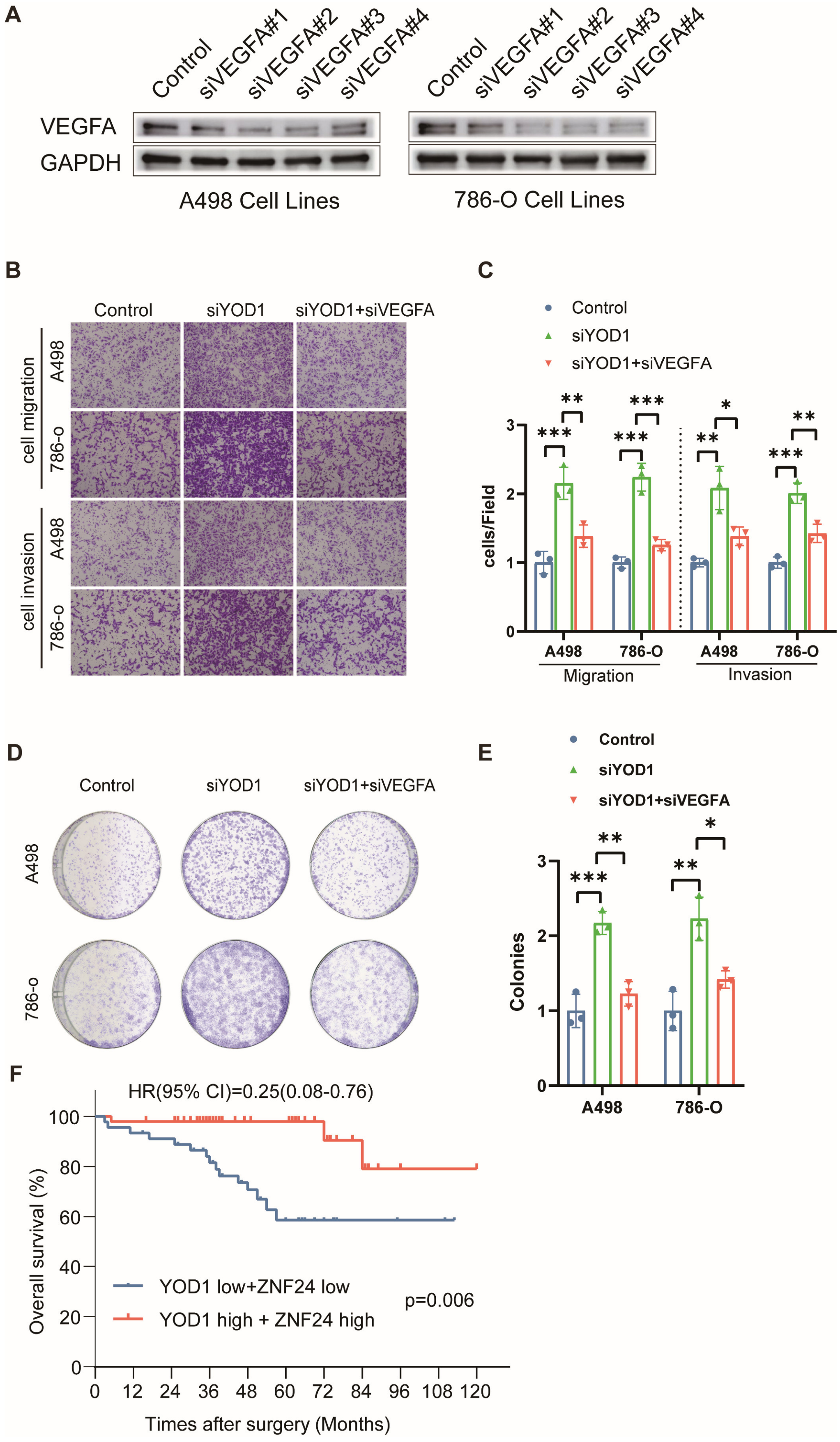

Fig S5. YOD1 inhibits ccRCC progression by inhibiting VEGFA and the clinical value of YOD1 and ZNF24. **A** Efficacy of VEGFA siRNAs. **B-E** Cells were transfected with YOD1 siRNAs or VEGFA siRNAs (with or without co-transfection of VEGFA siRNAs), followed by transwell migration and invasion assays (**B, C**) and colony formation assays (**D, E**). **F** Prognostic value of combining YOD1 and ZNF24 low and high expression data into one dataset in ccRCC cohort 2.

Supplementary Tables

Table S1. Primers used in this study

Gene name	Forward Primer	Reverse Primer
GAPDH	CCCACTCCTCCACCTTTGAC	CCACCACCCTGTTGCTGTAG
TNFAIP3	CTCAACTGGTGTCGAGAAGTCC	TTCCTTGAGCGTGCTGAACAGC
OTUB1	TGGATGACAGCAAGGAGTTG	TCTCCACCTGCTCAATCAGG
OTUB2	TGGCTTTGAGGAGCACAAGT	GGTCGTTGAACACCTTCAGC
OTUD1	CAGTTGGCTCAGTAACGGACAC	GATTTGGCAAGTTCTTCGTCGCG
OTUD3	CAGACGGATTTTCAGATGC	TCAGAGTCCATTCCCTTTG
OTUD4	GGCCTGTATTCACTATCTTCG	TGTCCTACCCATTCCTGTG
OTUD5	CAGGCTACAACAGTGAGGACGA	GAAGCCCTTCTTGTCTCGTAGG
OTUD6A	GTTCAGCGTGTCTGTGGAGATG	GCACGATGTTGTCGCAGTAGATC
OTUD6B	CTGCTGAGAAGGCATCGCAAAG	GCCACATCTTCGGTGAGTTGCT
OTUD7A	CCTGACGGATTCTGAGCACAAG	GTTCAGCTTGGCTTCTAGCGAC
OTUD7B	TCTCAGAGGCTGCTTCCTTTGG	CGCCTTTTCAACGCTTCCTTCTC
VCPIP1	TGGTCTTATGCAAAGGGACTTCC	GGTTTTGCCAGGCAGATGTGGA
YOD1	CCTGTGCTTACCAGAACCGT	AGACTCCTCCTTCGACGACA
ZRANB1	CGCTTGCTGAATCGTCCTTCTG	GTTCAGGACACACCATTGCTGG
ZNF24	GTGACAGTGCTGGAGGATTTGG	GGTTCTCCACAGCATCAAGCTC

Table S2. Sequences of siRNAs used in this study

**siRNA**	sense（5'-3'）	antisense（5'-3'）
siYOD1#1	CCAUCCAAUCUGGUGACAUTT	AUGUCACCAGAUUGGAUGGTT

siYOD1#2	UCUAGUUACGUCAGGGAAATT	UUUCCCUGACGUAACUAGATT
siYOD1#3	GCCUCUUUACUAGUGUGUATT	UACACACUAGUAAAGAGGCTT
siYOD1#4	GGUUCUGCUUAUUUAUGAUTT	AUCAUAAAUAAGCAGAACCTT
siOTUD5	GGACUUUACCACCUACAUUTT	AAUGUAGGUGGUAAAGUCCTT
siOTUD6B	GCUAGACAGUUAGAAAUUATT	UAAUUUCUAACUGUCUAGCTT
siTNFAIP3	GGAUGAAGCUAACUUACCATT	UGGUAAGUUAGCUUCAUCCTT
siOTUD4	GGAACUAGACACGUUGGAATT	UUCCAACGUGUCUAGUUCCTT
siZRANB1	GGAGAAACUUUAGGAUAUATT	UAUAUCCUAAAGUUUCUCCTT
siOTUB1	UCACUGAAUUCACAAUUGATT	UCAAUUGUGAAUUCAGUGATT
siOTUD7B	GCAUCUAGGUACCAAUGGATT	UCCAUUGGUACCUAGAUGCTT
siOTUB2	GGAAGAGCAGGGAGAUCUUTT	AAGAUCUCCCUGCUCUUCCTT
siVCPIP1	GGACGACAGUCCAUGGUUATT	UAACCAUGGACUGUCGUCCTT
siOTUD6A	GCACUACAACUCCGUGACATT	UGUCACGGAGUUGUAGUGCTT
siOTUD7A	GAAGGCCCAUCGUUGUUGUTT	ACAACAACGAUGGGCCUUCTT
siOTUD3	GAGGAUCAAUGACAACUCATT	UGAGUUGUCAUUGAUCCUCTT
siOTUD1	GACAACUGGUGCAAACAAATT	UUUGUUUGCACCAGUUGUCTT
siZNF24#1	GCGAAGAGGGAUCAAGUAUTT	AUACUUGAUCCCUCUUCGCTT
siZNF24#2	GCAGUUUGUUGCCAUCCUATT	UAGGAUGGCAACAAACUGCTT
siZNF24#3	GGAUUUGGAGAGUGAACUUTT	AAGUUCACUCUCCAAAUCCTT
siZNF24#4	GGAGCUUCCUUCAGCAUUATT	UAAUGCUGAAGGAAGCUCCTT
siVEGFA#1	GGCAGAAUCAUCACGAAGUTT	ACUUCGUGAUGAUUCUGCCTT
siVEGFA#2	GCUUCCUACAGCACAACAATT	UUGUUGUGCUGUAGGAAGCTT

siVEGFA#3	CAGCACAACAAAUGUGAAUTT	AUUCACAUUUGUUGUGCUGTT
siVEGFA#4	CCGCAGACGUGUAAAUGUUTT	AACAUUUACACGUCUGCGGTT

Table S3. Sequences of shRNA targeting indicated genes

**Gene**	**Sequence**
shYOD1#1	GTTTACTGATGTCAACCGCTT
shYOD1#2	GCACTGGAATTAGCAGATGAA

Supplementary Methods

Western blotting (WB)

Western blot analysis was conducted to evaluate protein expression in cells. Proteins were extracted using RIPA buffer supplemented with phosphatase and protease inhibitors, and their concentrations were measured using a BCA assay kit (Beyotime, P0012). Proteins were separated by 10% SDS-PAGE and transferred onto a PVDF membrane (Cytiva, catalog number: 10600021). The membrane was blocked with 5% milk at room temperature for 1 hour and then incubated overnight at 4°C with the specified primary antibody. After three washes with Tris-buffered saline containing 0.1% Tween-20 (TBST), the membrane was incubated with an HRP-conjugated secondary antibody (1:5000 dilution) at room temperature for 1 hour. Following three additional TBST washes, protein bands were detected using an enhanced chemiluminescence (ECL) system and visualized with a gel imaging system.

Quantitative real time polymerase chain reaction (qRT-PCR)

Total RNA was extracted using TRIzol reagent (Beijing TransGen Biotech). Chloroform was added to the homogenized lysate to facilitate phase separation. Following centrifugation, the RNA-containing aqueous phase was carefully collected and precipitated with isopropanol. The RNA pellet was washed with 75% ethanol, air-dried, and resuspended in an appropriate volume of RNase-free water. RNA quality and purity were evaluated using a NanoDrop 2000 spectrophotometer.

First-strand complementary DNA (cDNA) was synthesized from 1 μg of total RNA using an mRNA reverse transcription kit (TransGen Biotech), in accordance with the

manufacturer's protocol. Quantitative PCR (qPCR) was subsequently performed using a qPCR kit (Servicebio Biotechnology, Wuhan, China) and gene-specific primers (Sangon Biotech, Shanghai, China). The transcription levels of target genes were normalized to GAPDH expression. The sequences of all primers used in this study are listed in Supplementary Table S1.

Plasmid construction, small interfering RNA, and lentivirus transfection

Plasmids, including the empty vector, YOD1, the enzymatically inactive YOD1 mutant (C160S), YOD1 domain deletion mutants, ZNF24, ZNF24 domain deletion mutants, the K308R mutant of ZNF24, and ubiquitin mutants (Ub-K6, Ub-K11, Ub-K27, Ub-K29, Ub-K33, Ub-K48, and Ub-K63), were purchased from Hanbio Biotechnology (Shanghai, China). All of the plasmids carried tags encoding Flag, Myc, or HA. The siRNA targeting YOD1 was obtained from GenePharma (Jiangsu, China), while the siRNAs targeting 14 OTU deubiquitinating enzymes were sourced from RiboBio (Guangzhou, China). The cells were transfected with the siRNAs and plasmids with Lipofectamine 2000 (Invitrogen, Carlsbad, CA, USA). The transfection efficiency and effects on target protein levels were analysed by Western blotting 48 h posttransfection. For stable overexpression of ZNF24, HEK-293T cells were cotransfected with the packaging plasmids pMD2 and psPAX and the vector plasmid pCDH-ZNF24, and the supernatant containing the lentiviral particles was collected after 48 hours. The cells were then transduced with the supernatant containing polybrene (5 μg/mL), and the transfection efficiency was assessed after 72 hours. shYOD1 lentivirus was obtained from Genechem Technology (Shanghai, China). The relevant siRNA and lentiviral

sequences are provided in the Supplementary Table S2 and Table S3.

Assays for colony formation

For the colony formation assay, cells were seeded in a 6-well plate at a density of 500 cells per well and incubated at 37 °C for 10–14 days, with the culture medium replaced every 3 days. After incubation, the cells were washed 1–2 times with sterile PBS buffer, and 500 μL of 4% paraformaldehyde was added to each well for fixation at room temperature for 20 minutes. The fixative was then discarded, and 1 mL of 1% crystal violet solution was added to each well for staining at room temperature for 20 minutes. The residual dye was washed off with water, and the plates were air-dried before imaging.

Transwell assay

For migration and invasion assays, complete medium supplemented with 20% fetal bovine serum was added to a 24-well plate (600 μL per well), and the transwell chamber was placed into the well. For invasion assays, the chamber was pre-coated with a 1:4 dilution of Matrigel. The cells were digested, resuspended in 200 μL serum-free medium, and seeded into the upper chamber, followed by incubation at 37°C for 24 hours. Afterward, the chambers were washed three times with PBS, fixed with 4% paraformaldehyde and stained with 1% crystal violet solution at room temperature for 20 minutes, respectively. The chambers were then washed by water, and the upper surface of the chamber was gently wiped with a cotton swab to remove non-migrated or non-invaded cells. After air-drying at room temperature, five randomly selected fields of view were imaged under a microscope, and statistical analysis was performed.

Tube formation assays

For tube formation assays, 96-well plates were pre-coated with Matrigel (without growth factors) and incubated at 37°C for 30 minutes until solidified. Human umbilical vein endothelial cells (HUVECs) were then resuspended in serum-free cell culture medium, and 100 μL of the cell suspension (1×10^4 cells per well) was seeded onto the solidified Matrigel. After culturing for 1-4 hours, tube formation was photographed under a microscope and quantitatively analyzed using ImageJ.

Immunofluorescence staining

ccRCC cells were seeded on chamber slides and incubated for 24 hours until they reached approximately 50% confluence. After aspirating the culture medium, cells were fixed with 4% paraformaldehyde at room temperature for 20 minutes, followed by three washes with PBS (5 minutes each). The cells were then permeabilized with 0.5% Triton X-100 at room temperature for 20 minutes and blocked with 3% BSA for 1 hour at room temperature. Subsequently, cells were incubated overnight at 4°C with anti-YOD1 (Proteintech, 25370-1-AP) and anti-ZNF24 (Santa Cruz, sc-393359) primary antibodies. The next day, the slides were washed three times with PBS and incubated for 1 hour at room temperature with fluorescently conjugated secondary antibodies (CoraLite488 goat anti-rabbit IgG, SA00013-2; CoraLite594 goat anti-mouse IgG, SA00013-3; Proteintech). After three additional washes with PBS, the cell nuclei were stained with DAPI. Slides were then washed with PBST solution for 5 minutes, repeating the wash four times to remove excess DAPI staining. Finally, anti-fluorescence quenching mounting solution was applied, the slides were covered with

coverslips, and sealed. Immunofluorescence intensity was examined under a confocal fluorescence microscope at wavelengths of 488 and 594 nm.

Immunohistochemistry (IHC)

Paraffin-embedded tissue sections were subjected to antigen retrieval and peroxidase blocking following baking, dewaxing, and hydration steps. The sections were incubated overnight at 4 °C with the appropriate primary antibody, followed by a 30-minute incubation with the secondary antibody at 37 °C, prior to final development and sealing. Protein expression was quantified as the product of staining intensity (0-3) and staining area (0-4).

Patient-derived organoids (PDOs) culture

To prepare and culture patient-derived organoids from ccRCC, fresh tumor tissues were initially washed with ice-cold PBS containing penicillin-streptomycin and then sectioned into tissue blocks of approximately 1-3 mm^3. These pieces were enzymatically digested at 37 °C for 30 minutes in 10 mL of a specialized tissue digestion solution (bioGenous) within a 15 mL conical tube. Digestion was stopped by adding FBS upon observing cellular clumping under a microscope, and the cells were mixed with Matrigel (Corning) and plated into a 24-well plate. The Matrigel polymerized at 37 °C for 20 minutes before adding 500 μL of complete human kidney cancer organoid medium (bioGenous). The medium was refreshed every 3-5 days.

For genetic interventions, organoids were mechanically disaggregated into clusters using a pipette, centrifuged at 200-300×g for 3-5 minutes, and then resuspended in a complete medium infused with either control or shYOD1 lentiviral particles

(Genechem). This suspension was incubated at room temperature for 30 minutes, followed by a 5-minute cooling period on ice. A same volume of Matrigel was subsequently incorporated into the suspension, which was then aliquoted in 50 μL drops into a 24-well plate and allowed to polymerize. After gel polymerization, the wells were topped with complete media, replaced every three days. Organoid growth was assessed by counting the number of organoids per well on day 14.
