# Supplementary material for: The deubiquitinase YOD1 suppresses tumor progression by stabilizing ZNF24 in clear cell renal carcinoma
Source: Cell Death Dis. 2025 Apr 24;16(1):334. doi: 10.1038/s41419-025-07673-2 (PMC12022293; doi:10.1038/s41419-025-07673-2)

Figure 1

1G YOD1

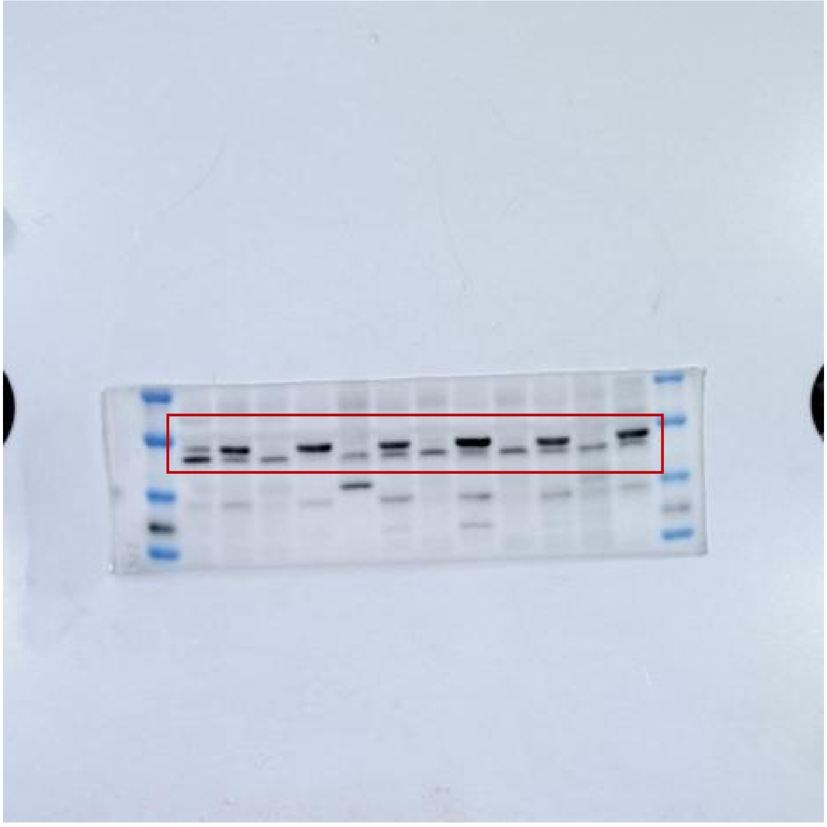

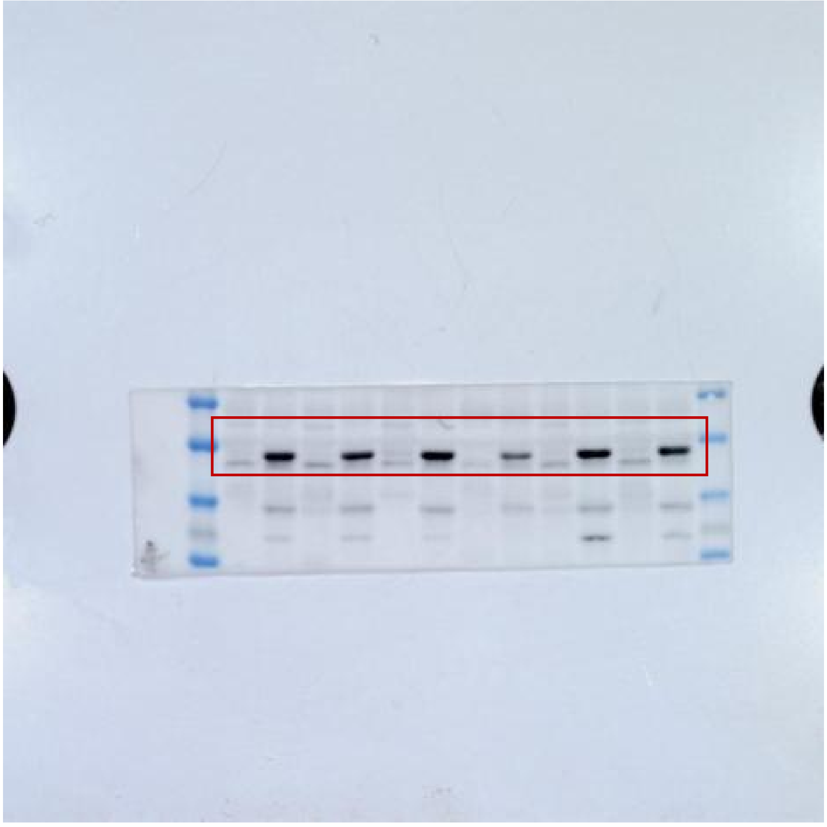

1G GAPDH

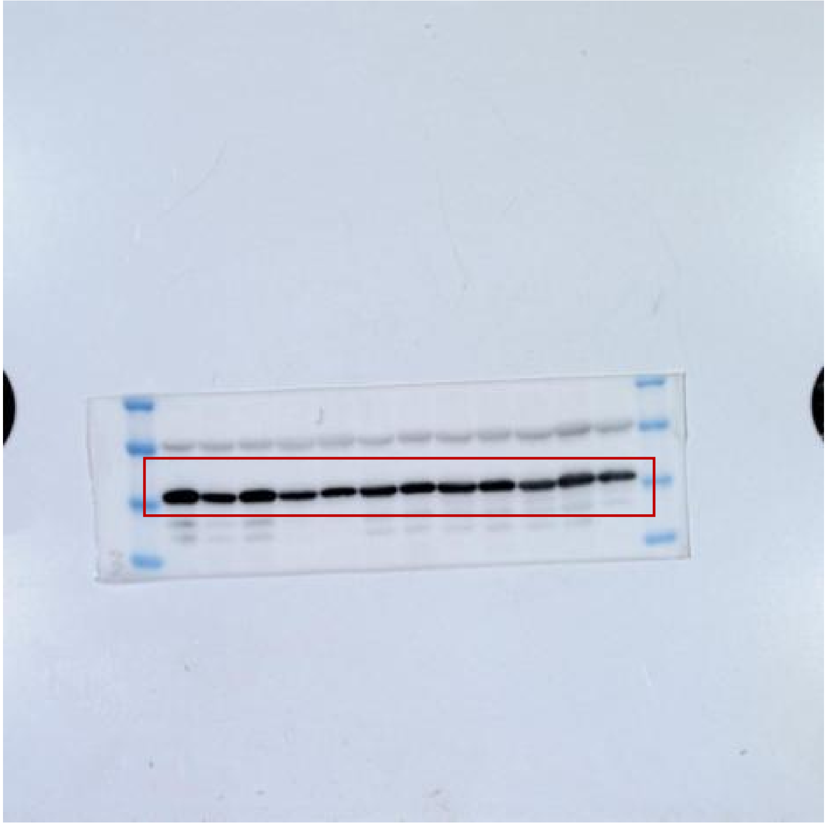

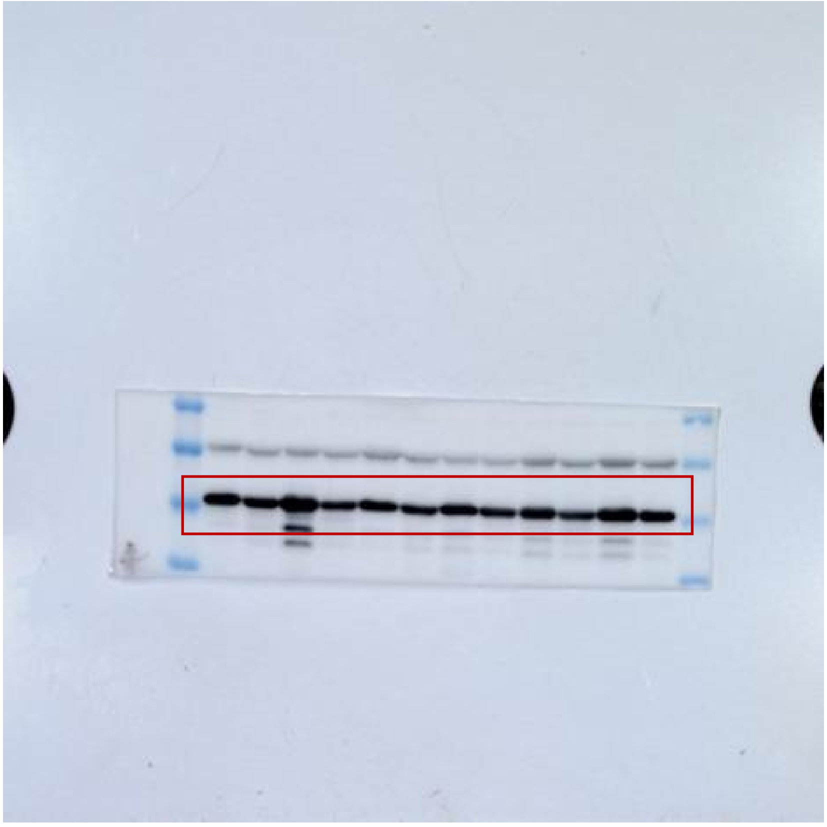

1I YOD1

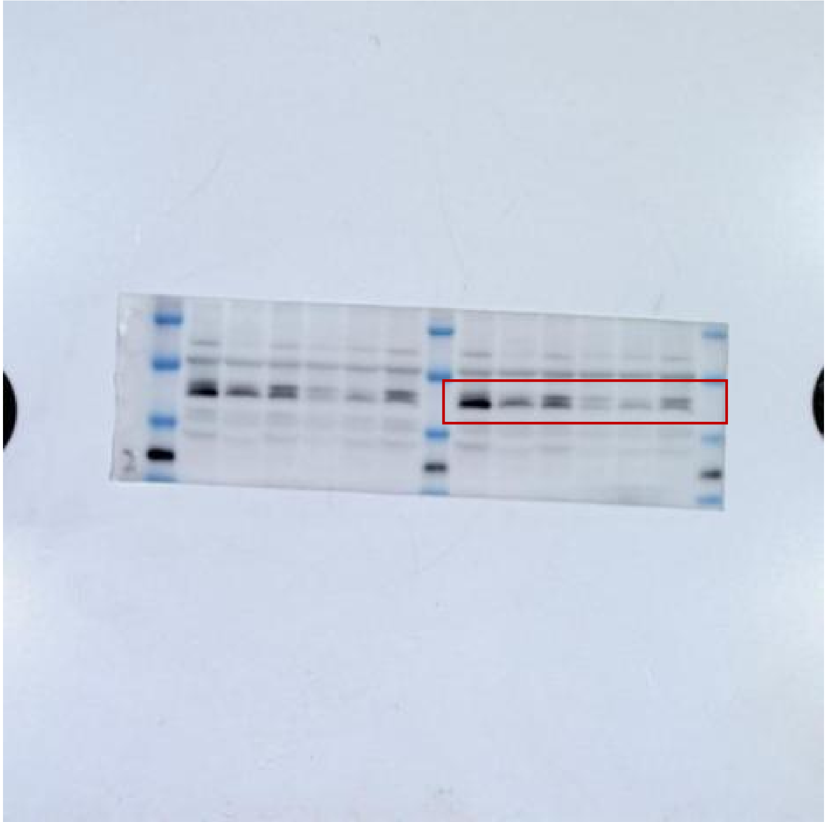

1I GAPDH

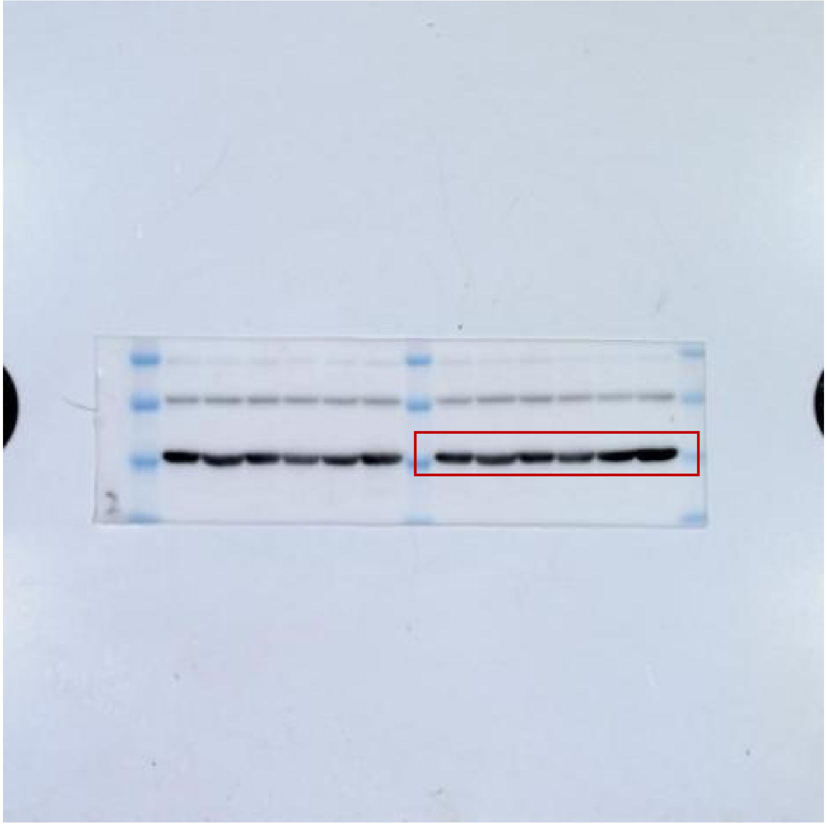

Figure 2

2A left: A498; right: 786-O YOD1

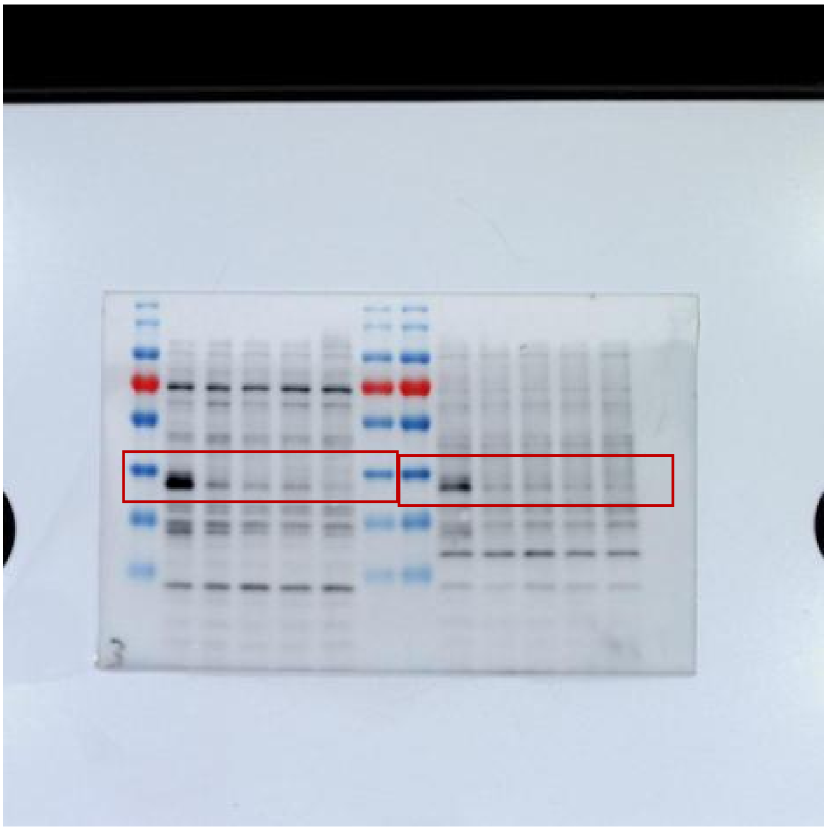

2A left: A498; right: 786-O GAPDH

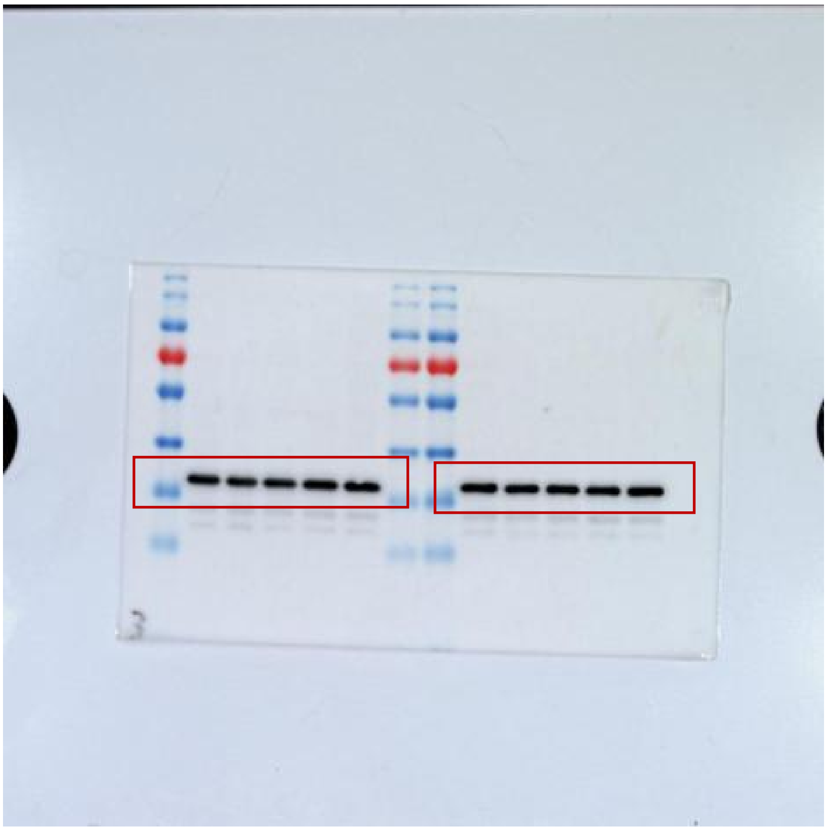

2B left: A498; right: 786-O YOD1

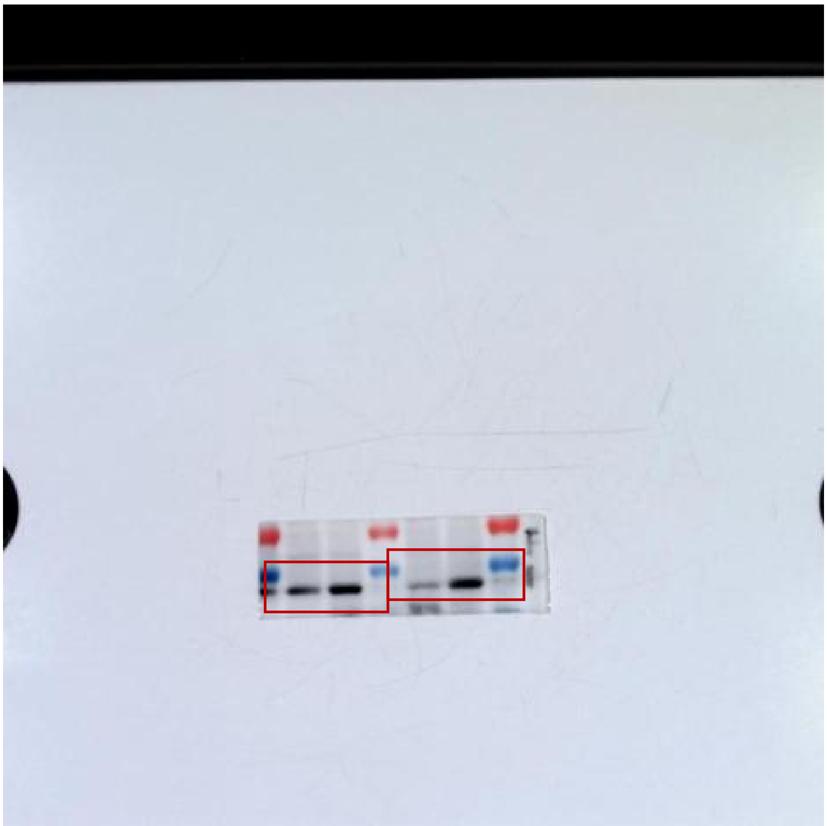

2B left: A498; right: 786-O GAPDH

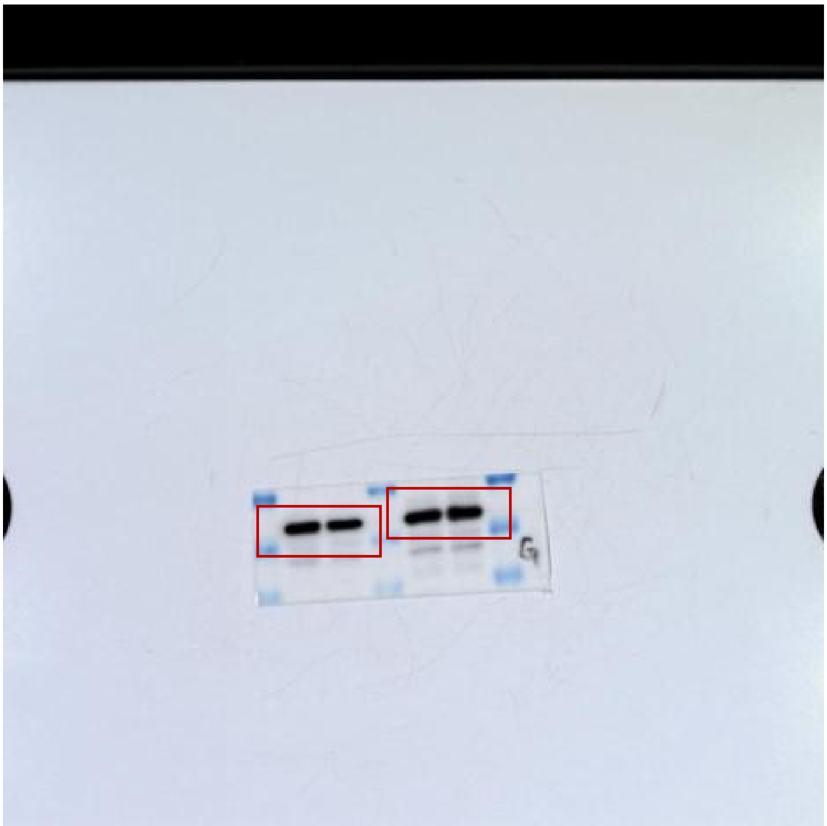

Figure 3

3E A498 ZNF24

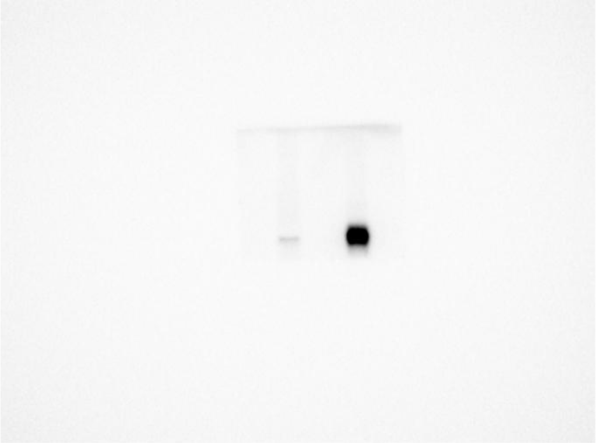

3E A498 YOD1

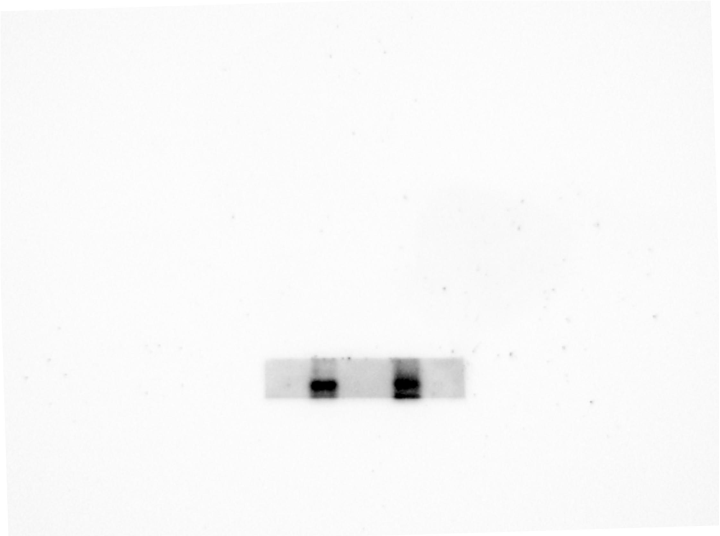

3E 786-O ZNF24

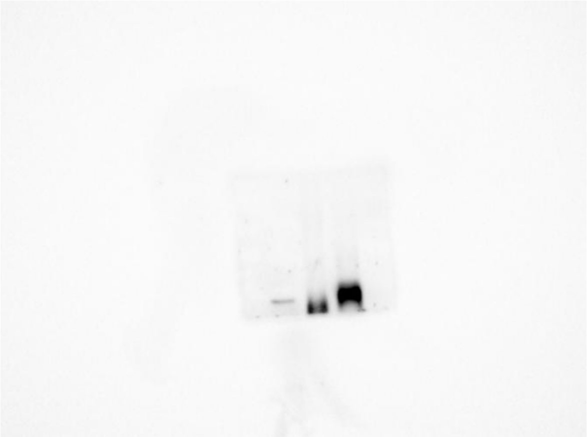

3E 786-O YOD1

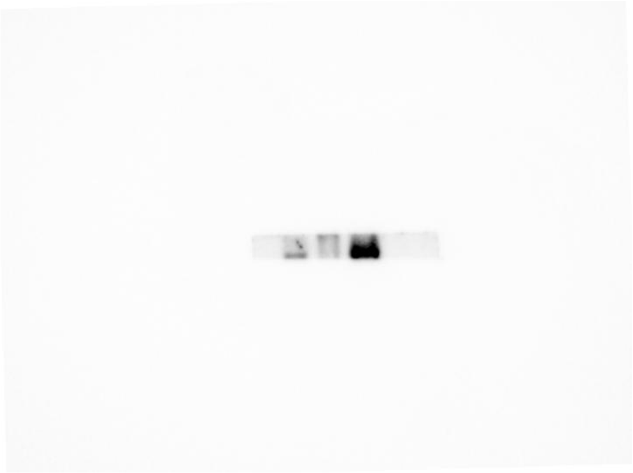

3F 786-O ZNF24

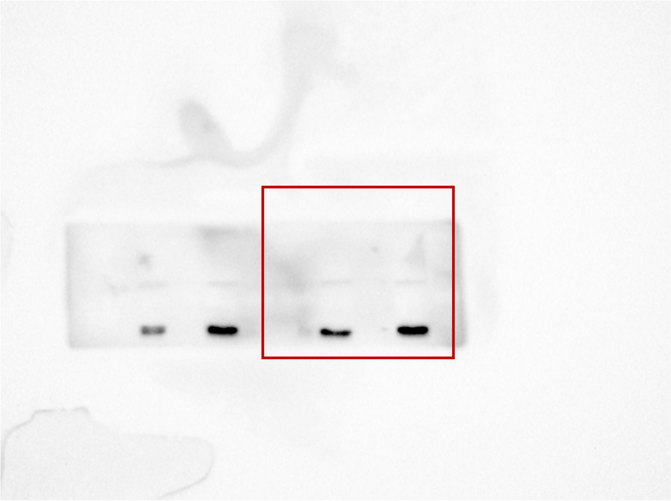

3F 786-O YOD1

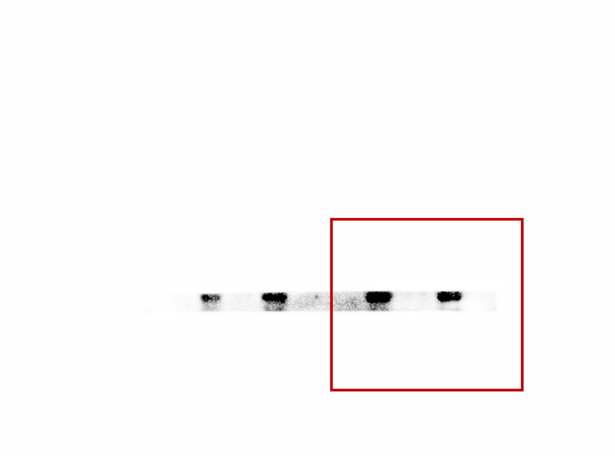

3F A498 ZNF24

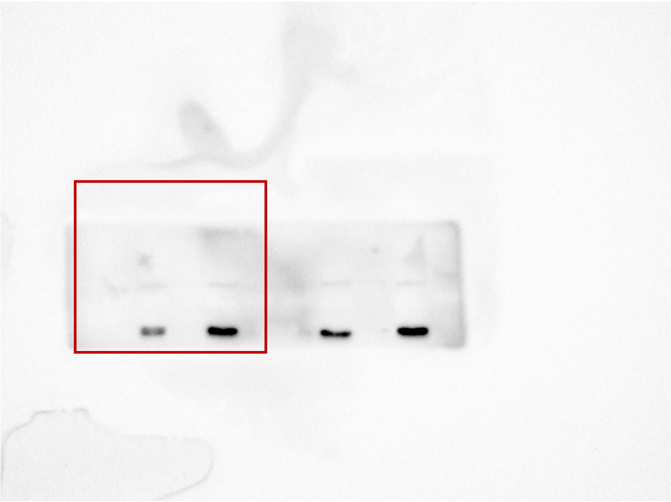

3F A498 YOD1

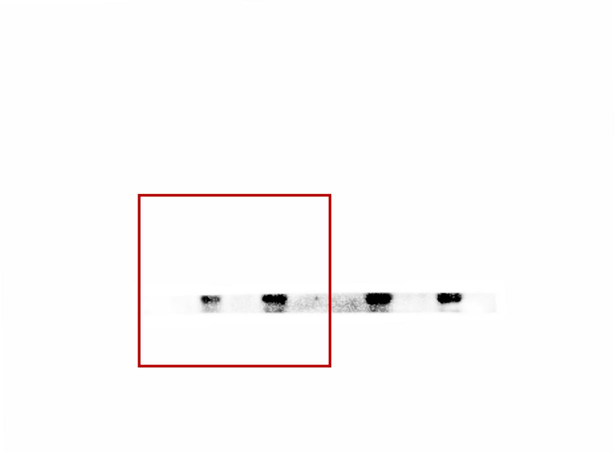

3G left Myc

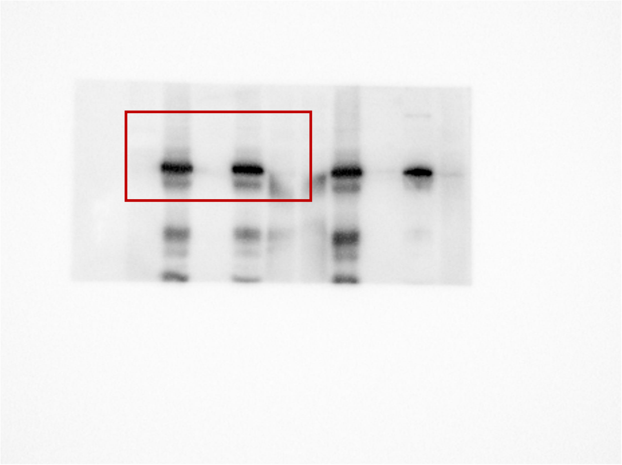

3G left Flag

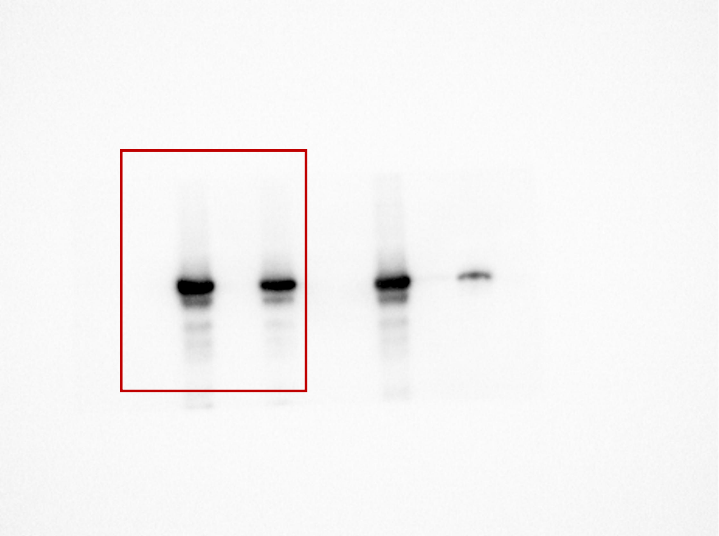

3G right Myc

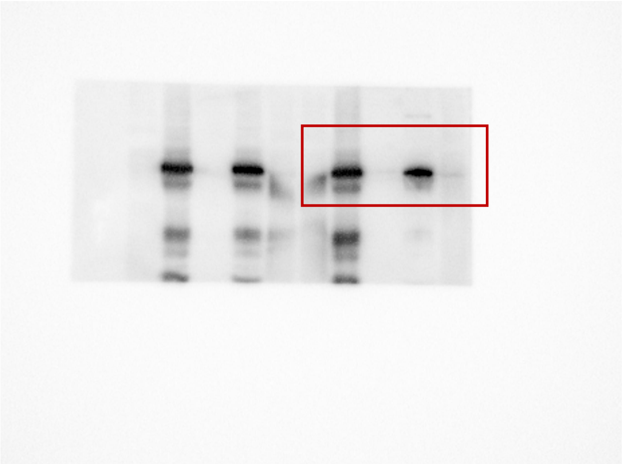

3G right Flag

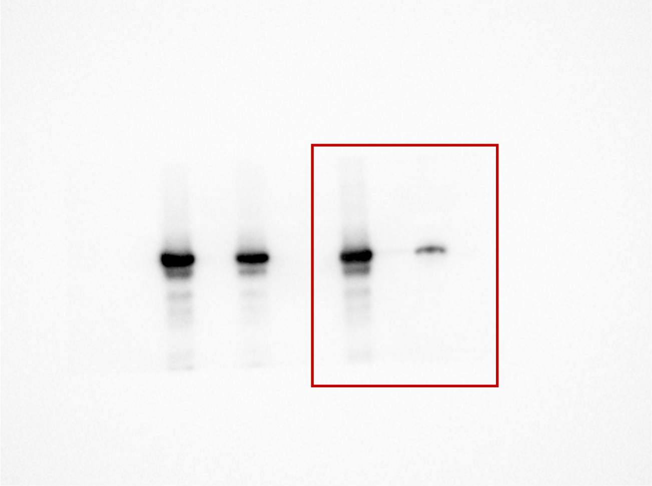

3I left Myc

3I left Flag

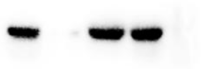

3I right Myc

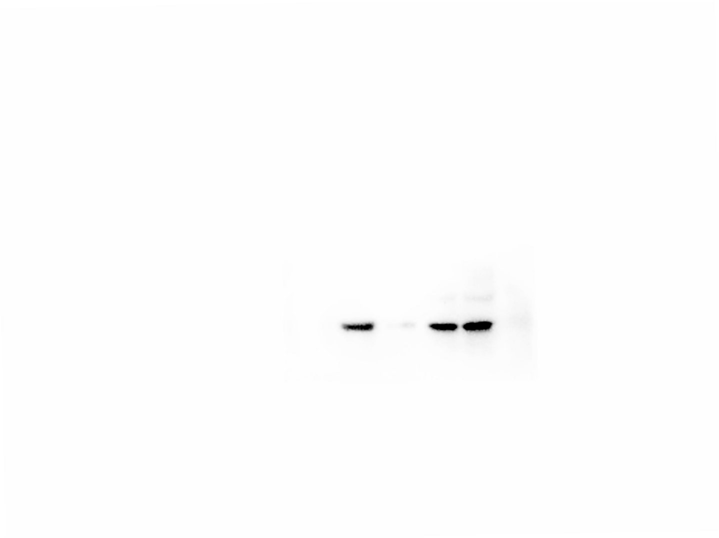

3I right Flag

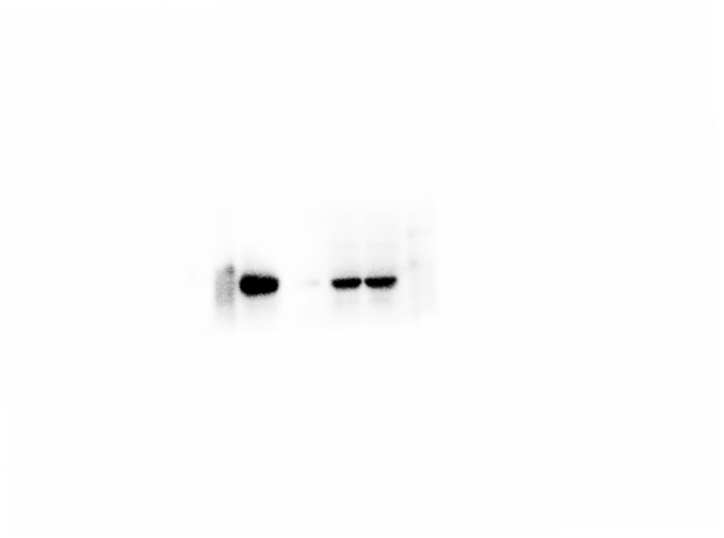

3H His

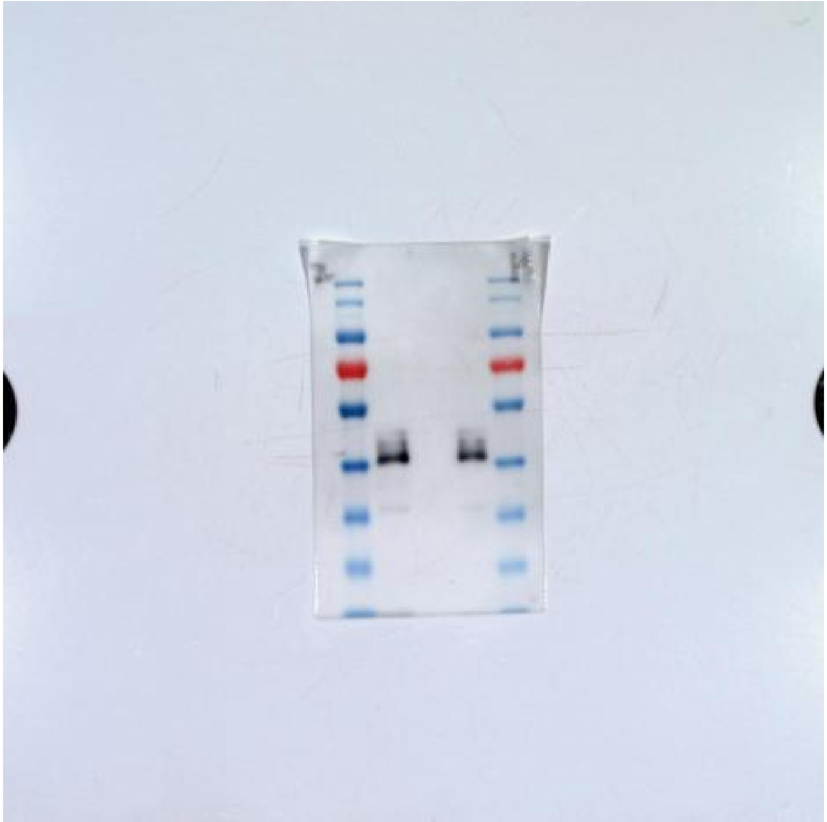

3J left FLAG

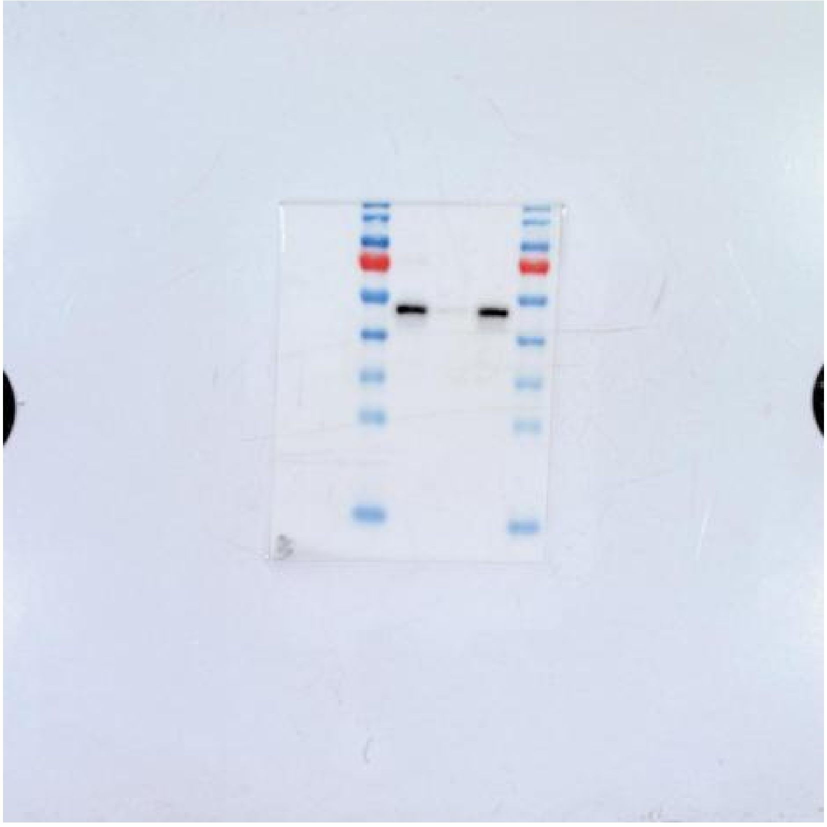

3J right FLAG

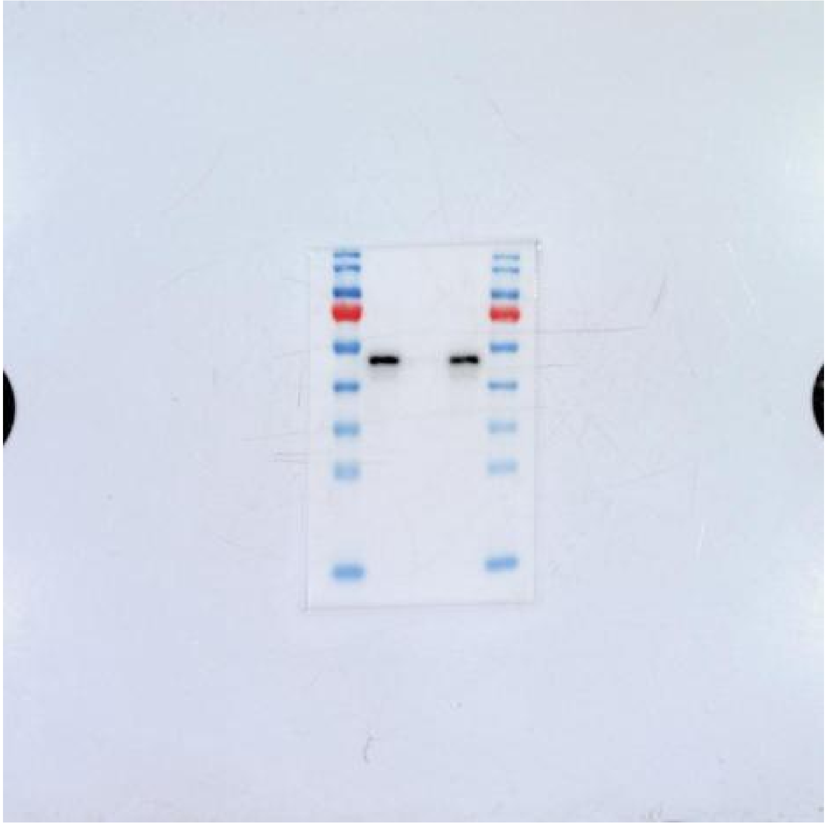

Figure 4

4B IP Flag

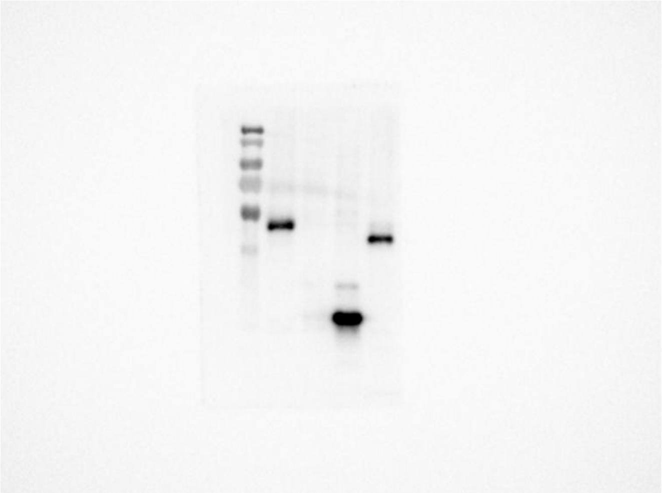

4B IP Myc

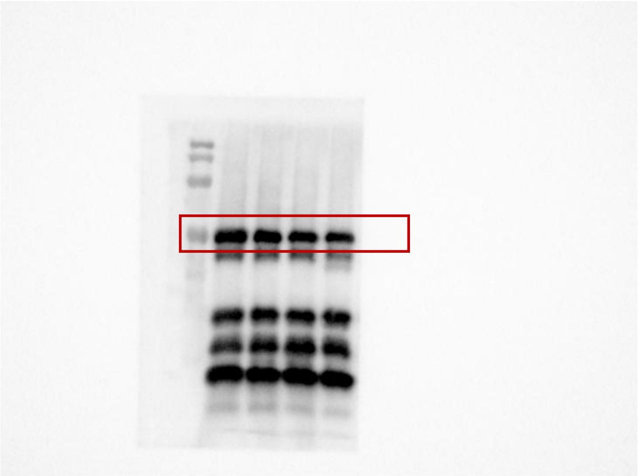

4B input Flag

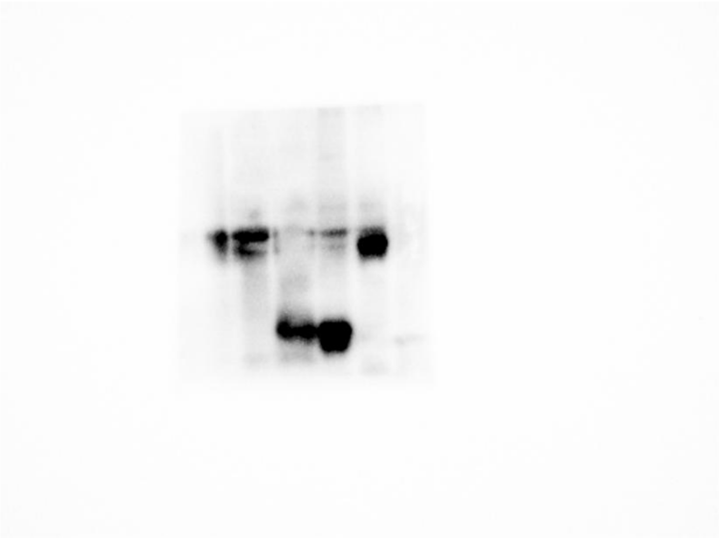

4B input Myc

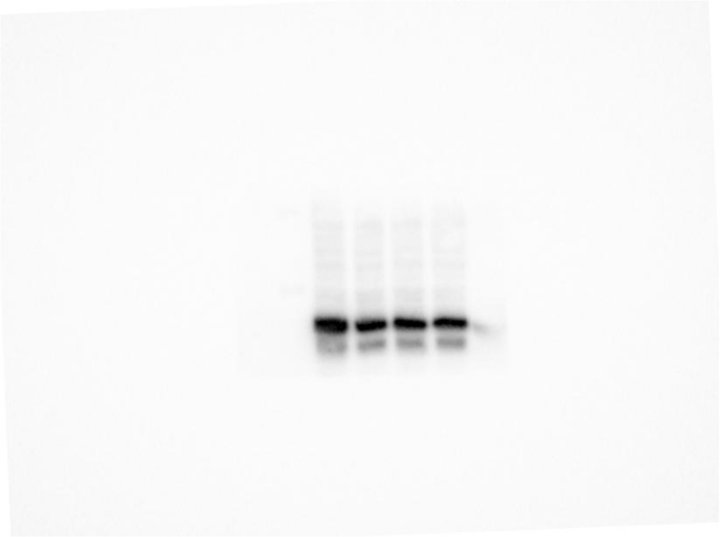

4B input GAPDH

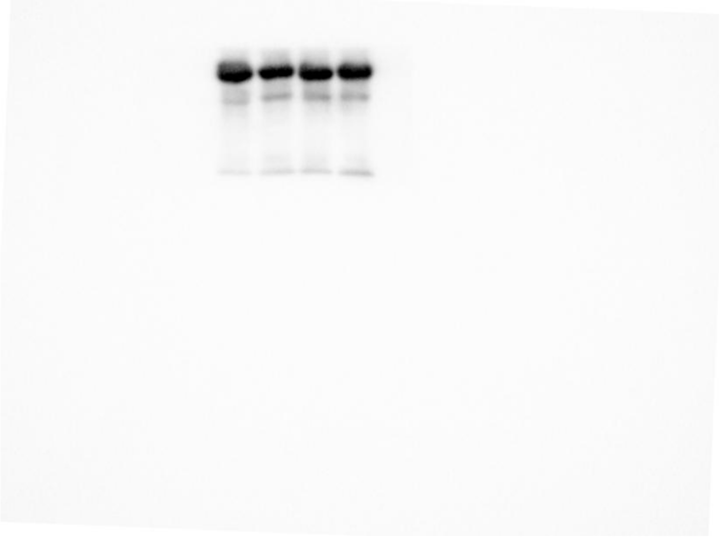

4C IP Flag

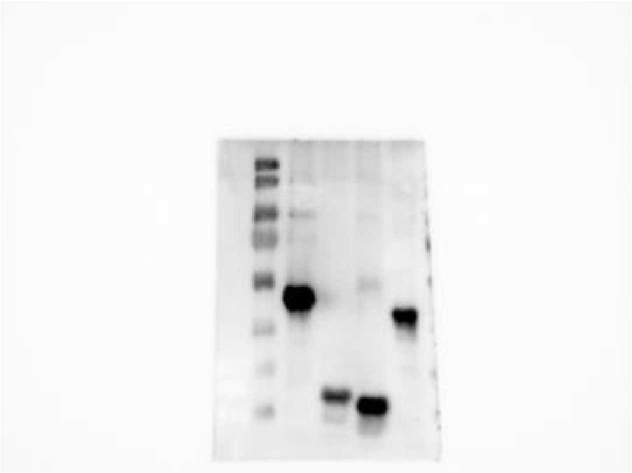

4C IP Myc

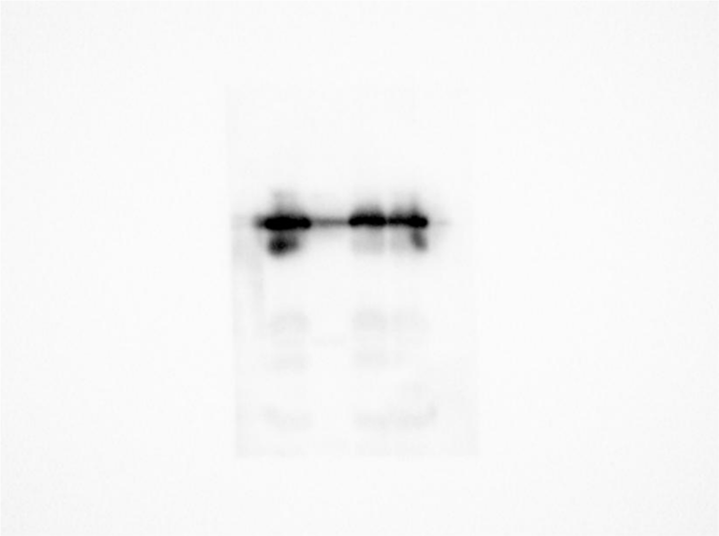

4C input Flag

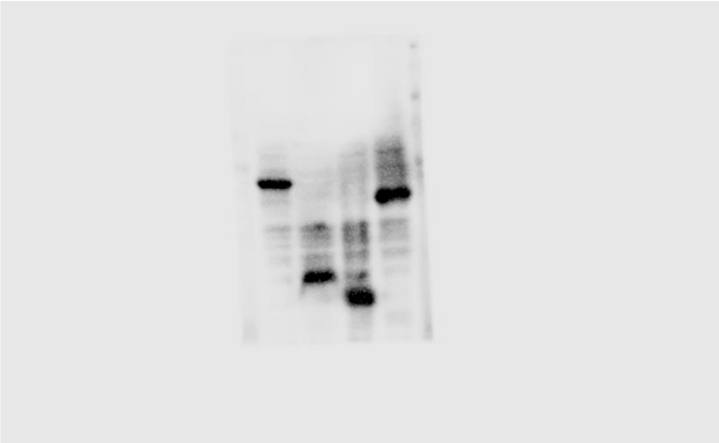

4C input Myc

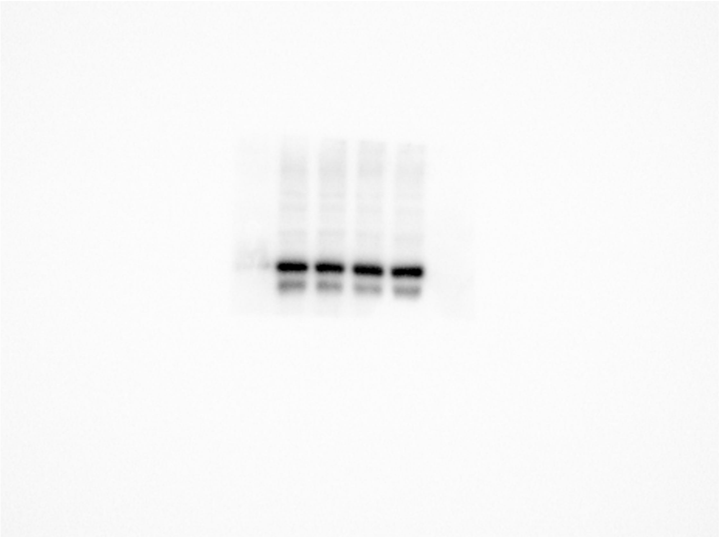

4C input GAPDH

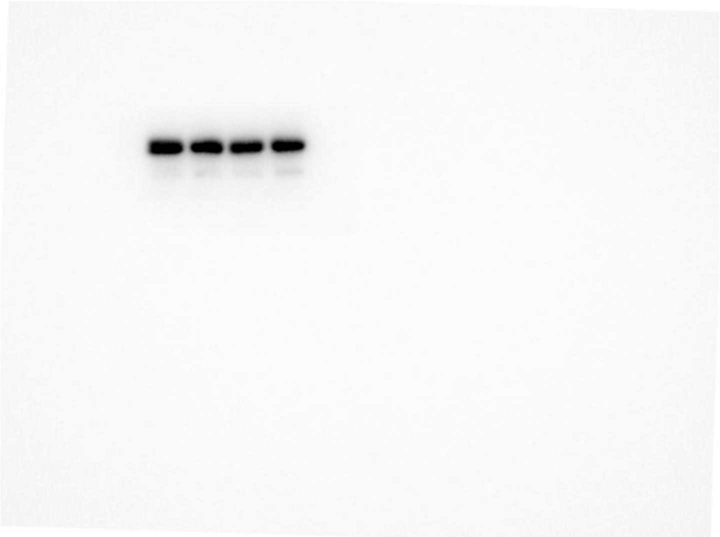

4D IP Flag

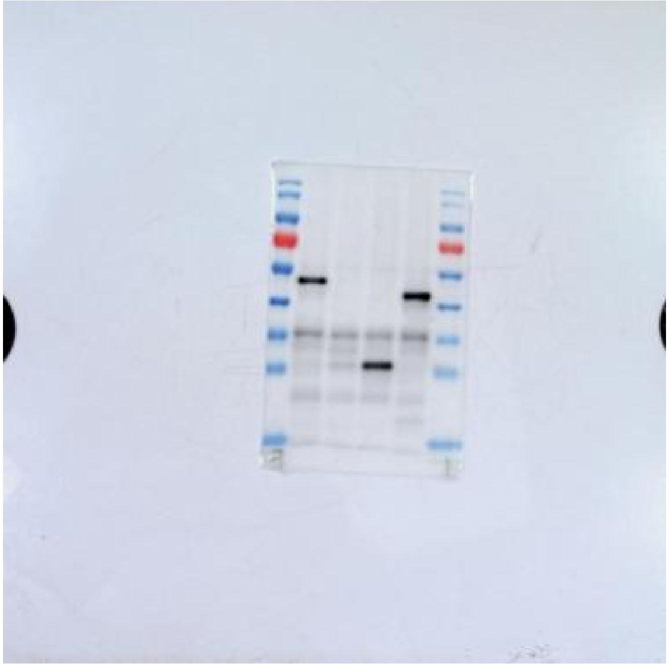

4D IP ZNF24

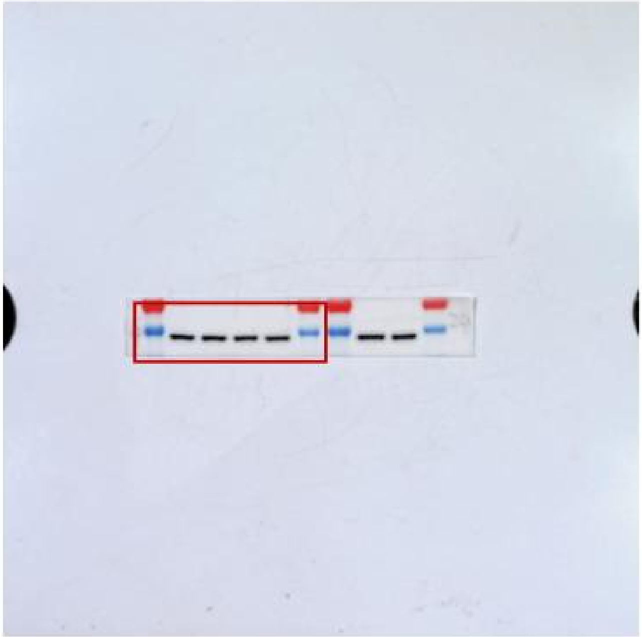

4D input Flag

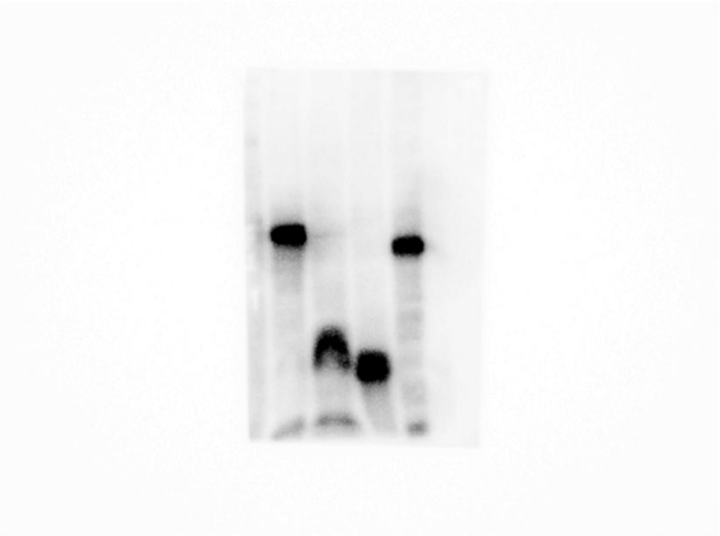

4D input GAPDH

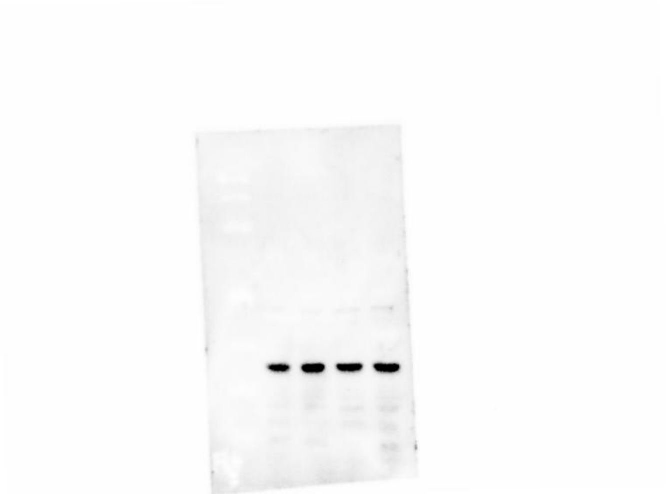

4E ZNF24

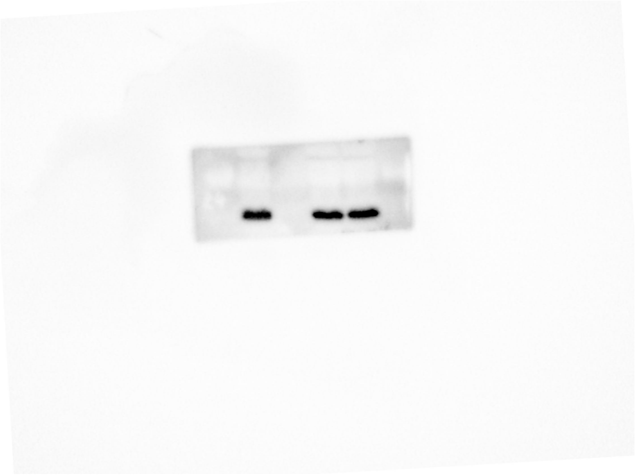

4E IP Flag

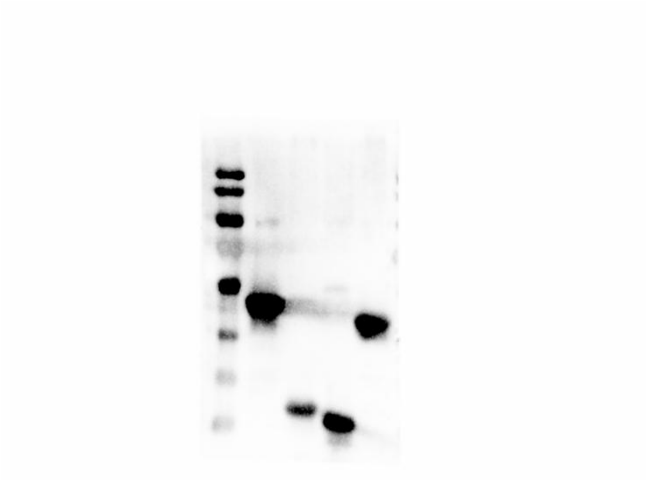

4E input Flag

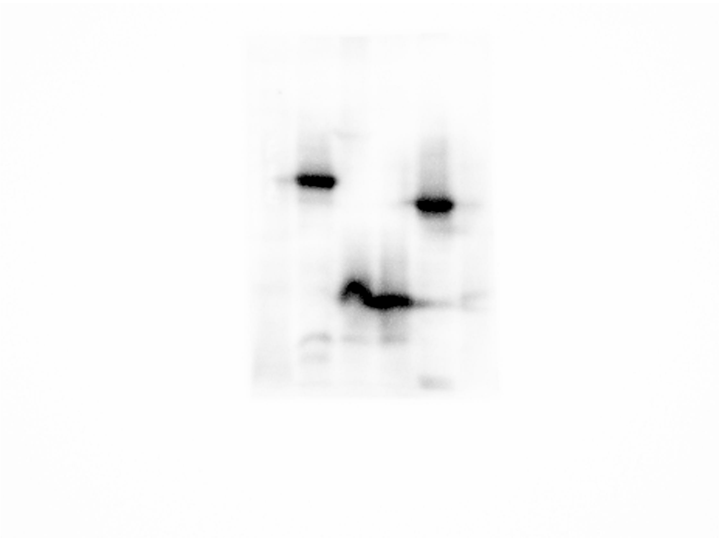

4E input GAPDH

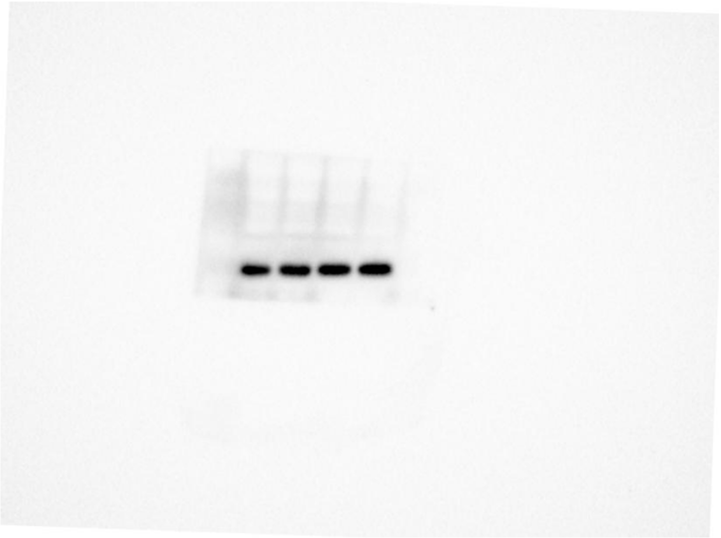

4G IP Myc

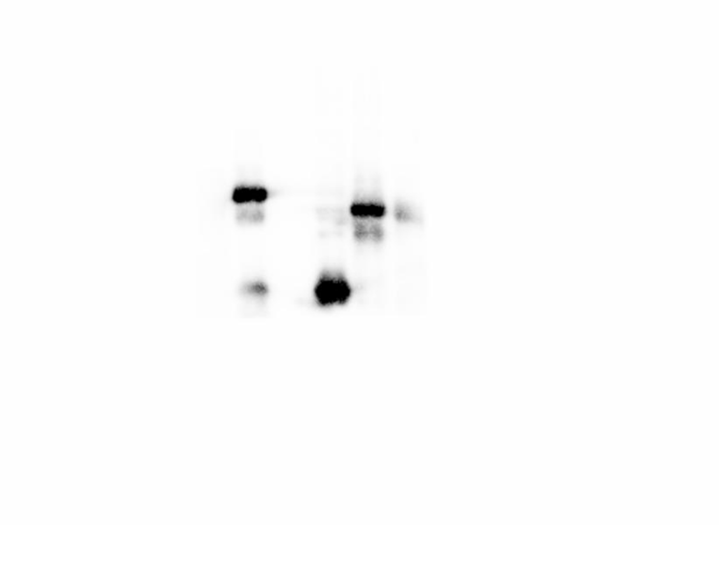

4G IP Flag

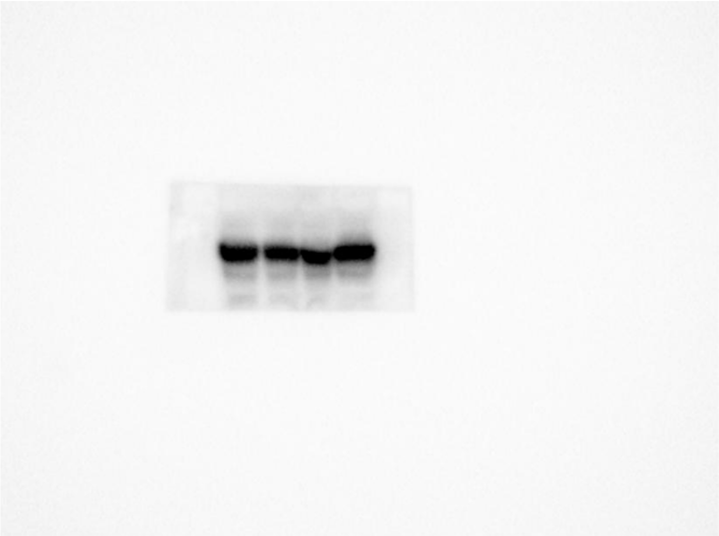

4G input Myc

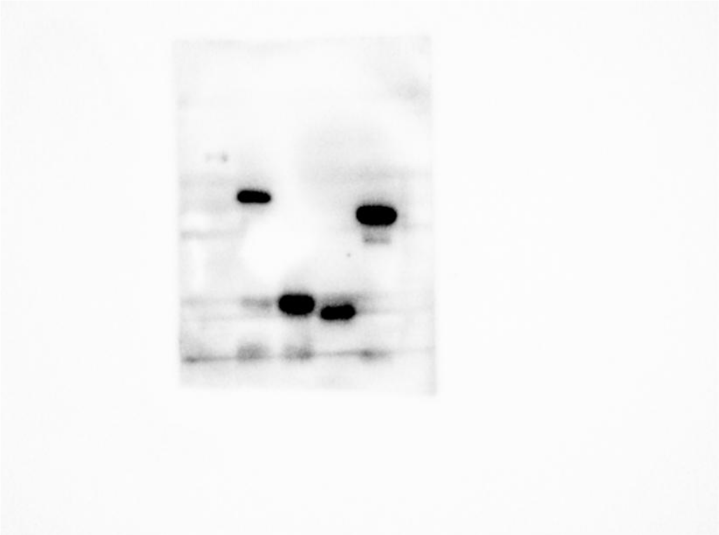

4G input Flag

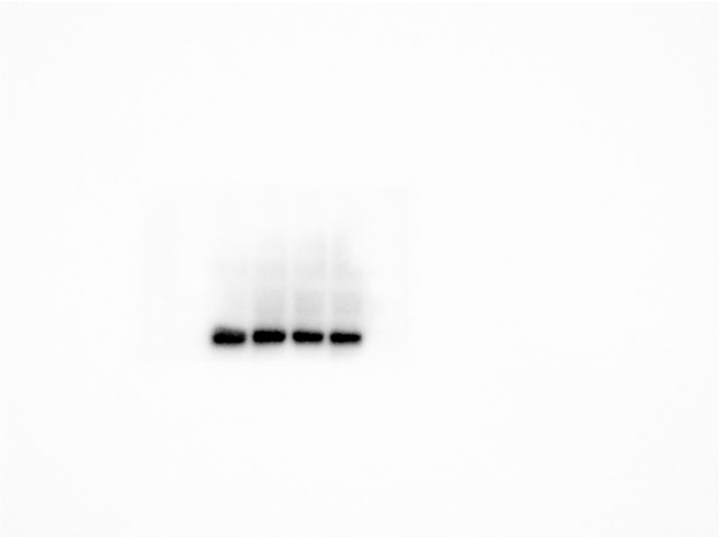

4G input GAPDH

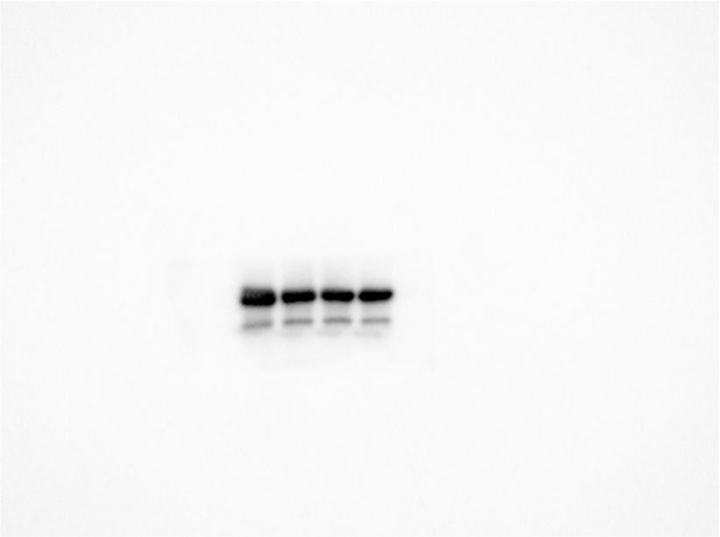

4H IP Flag

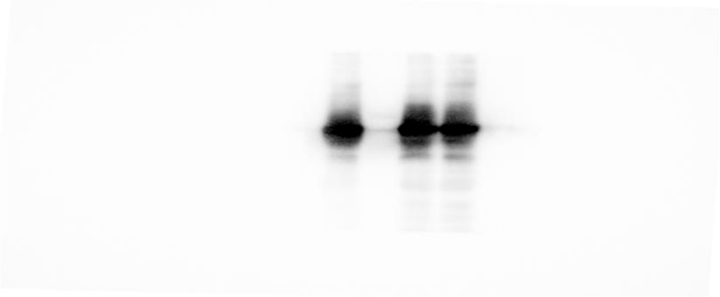

4H IP Myc

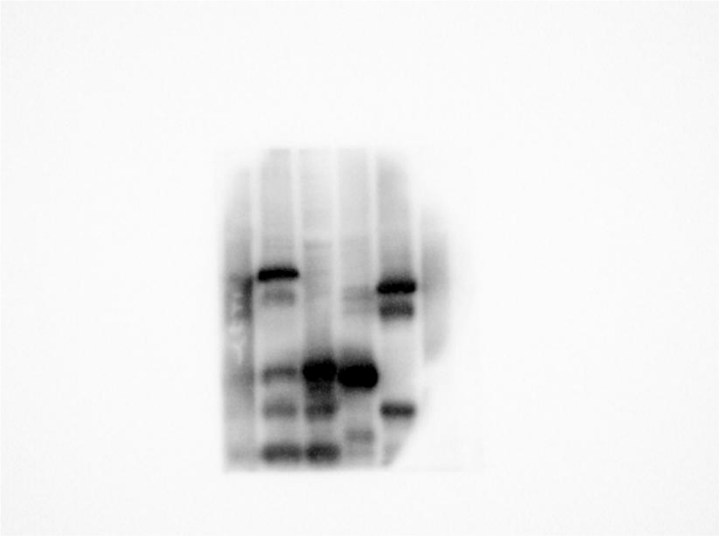

4H input Myc

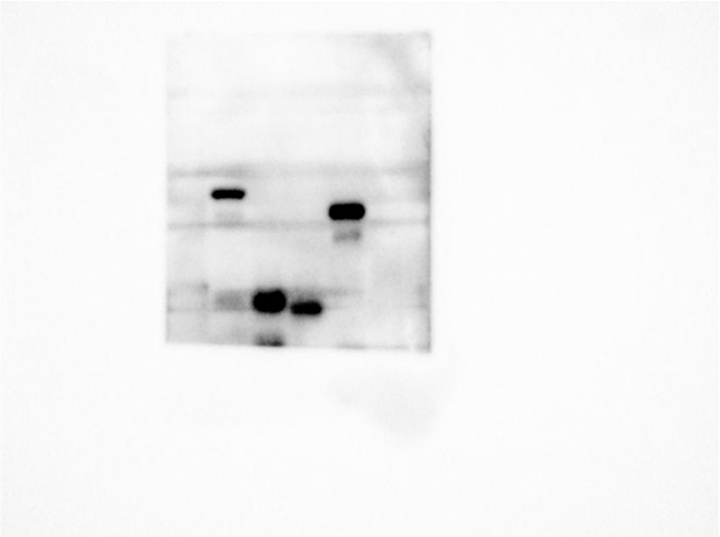

4H input Flag

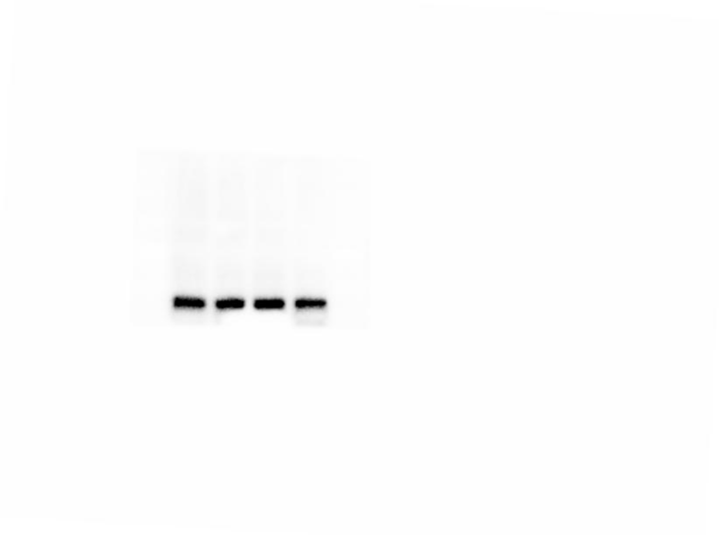

4H input GAPDH

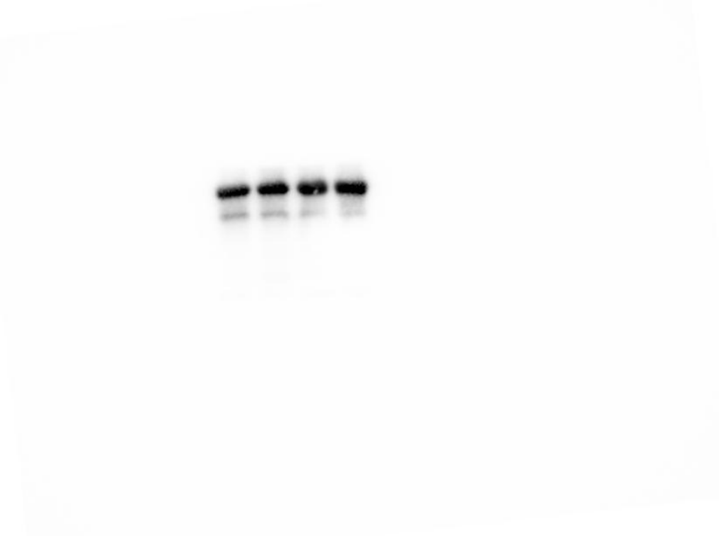

4I IP Myc

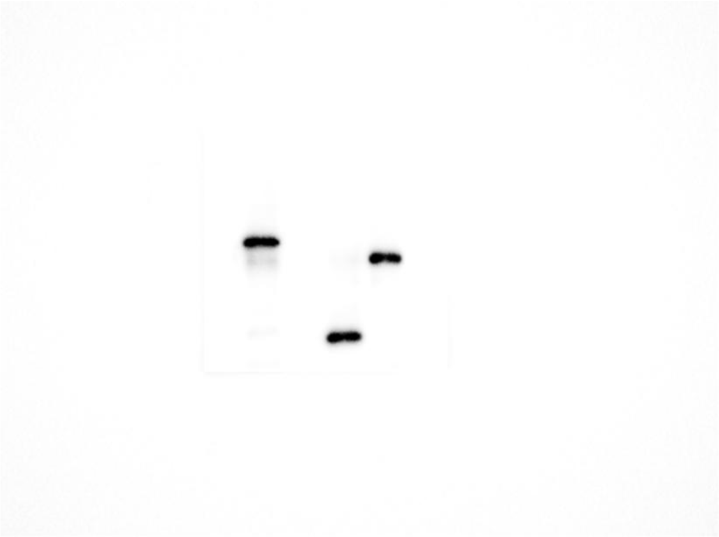

4I IP YOD1

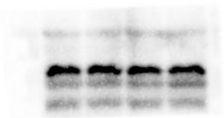

4I input Myc

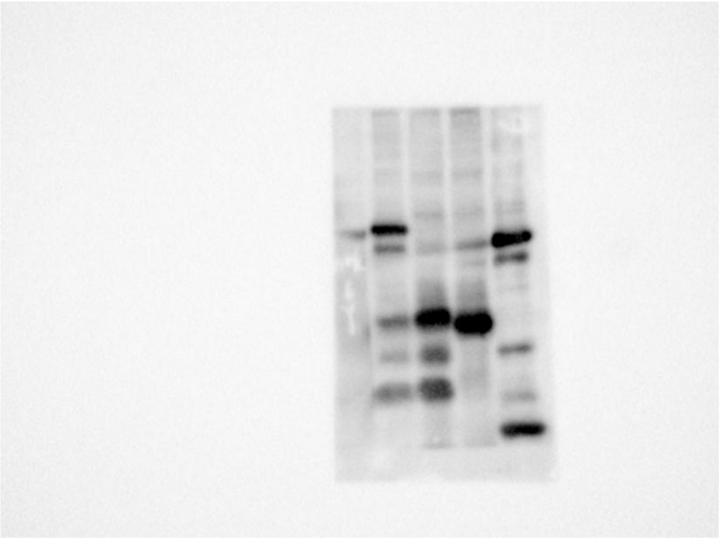

4I input GAPDH

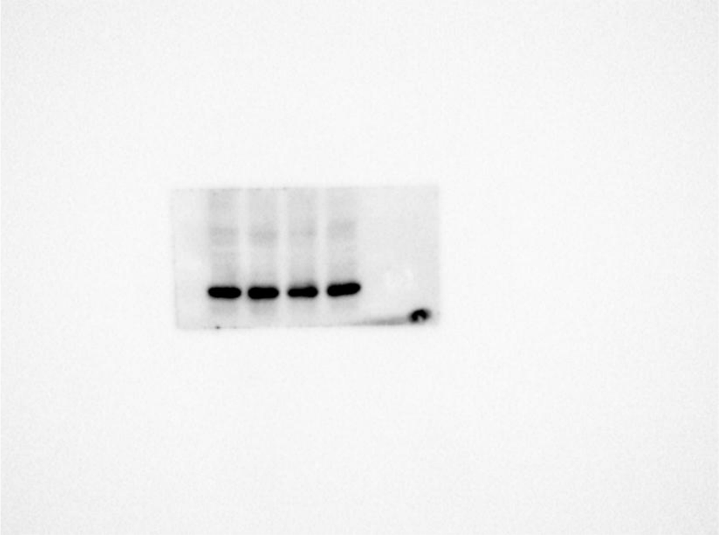

4J IP YOD1

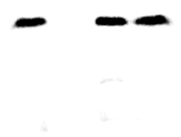

4J IP Myc

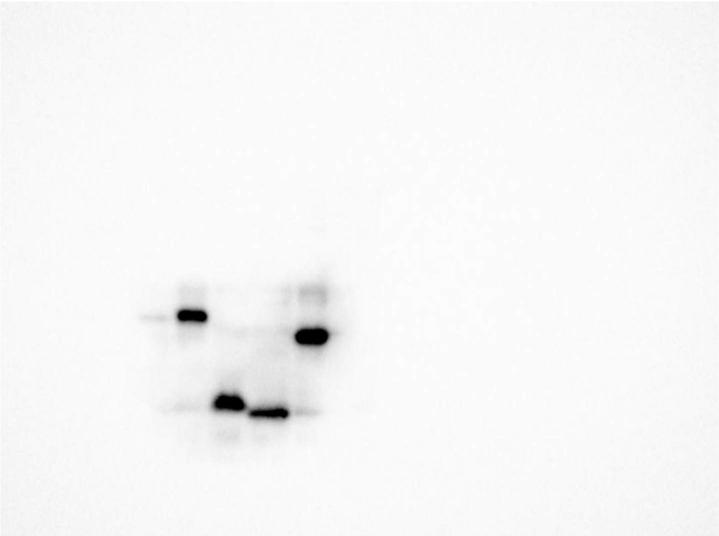

4J input Myc

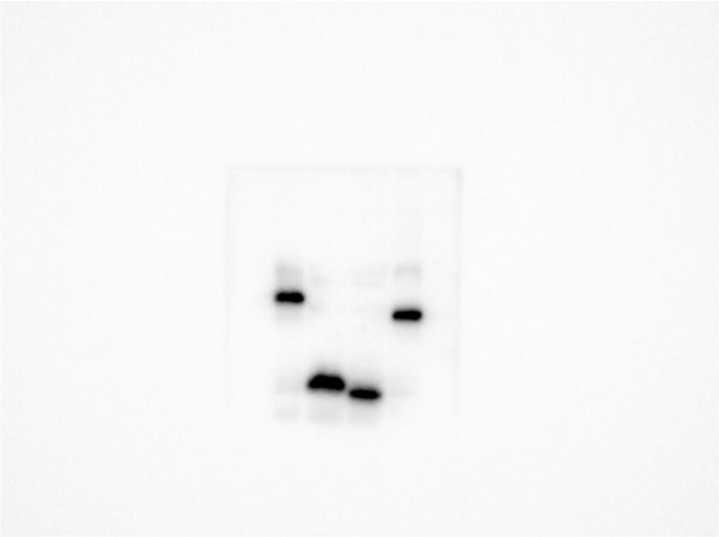

4J input GAPDH

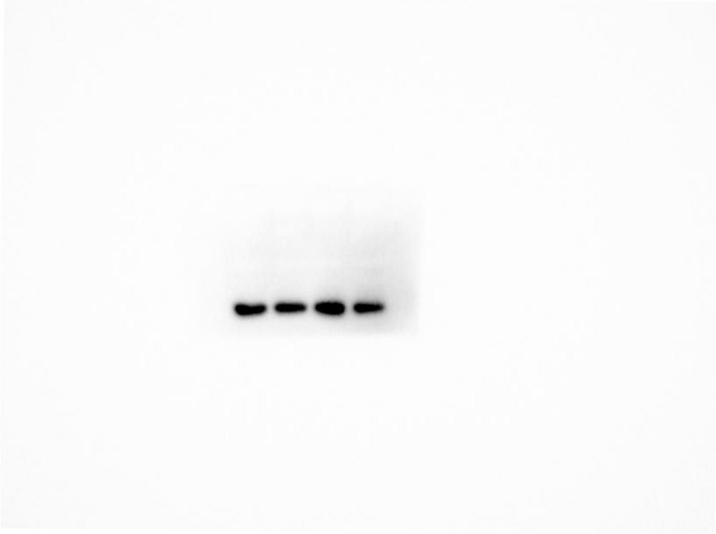

Figure 5

5A HA

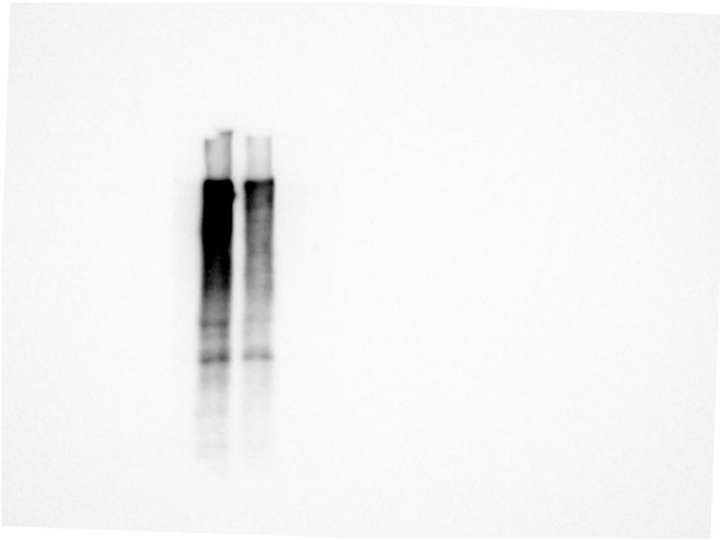

5A Myc

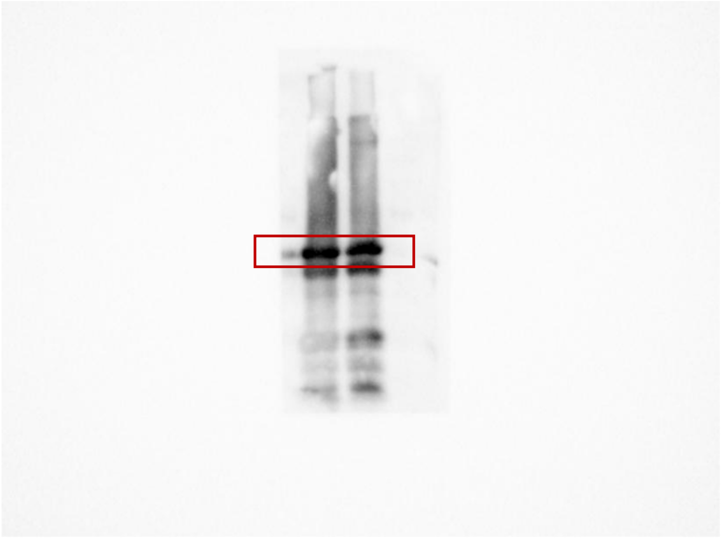

5A Flag

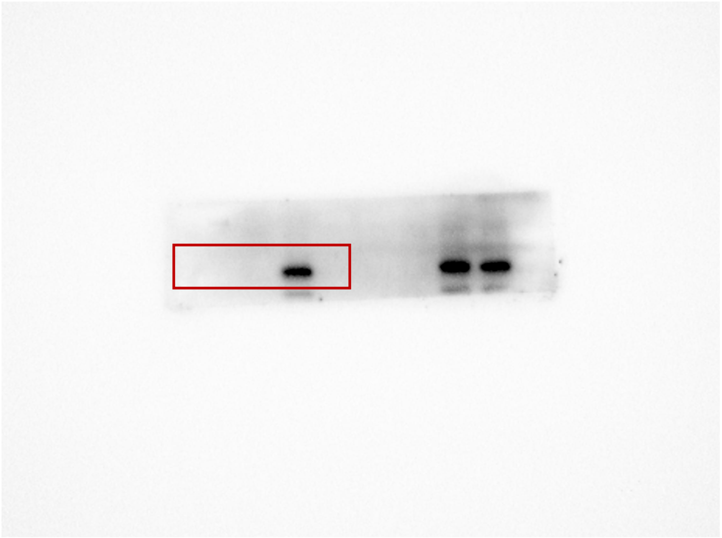

5A GAPDH

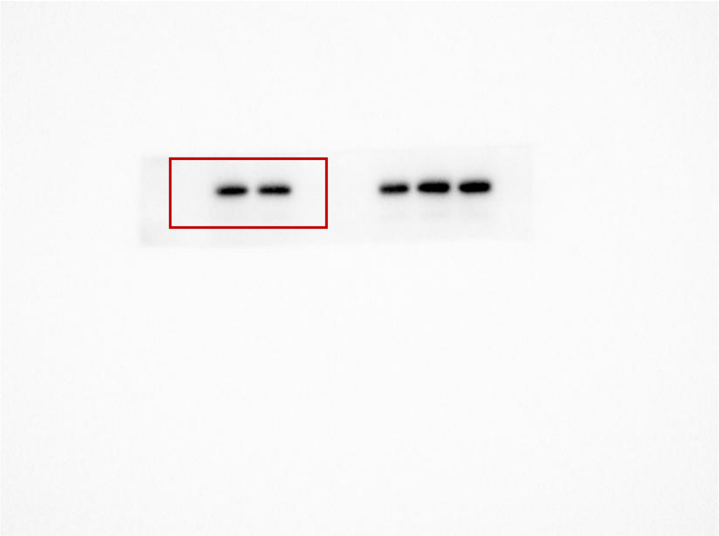

5B HA

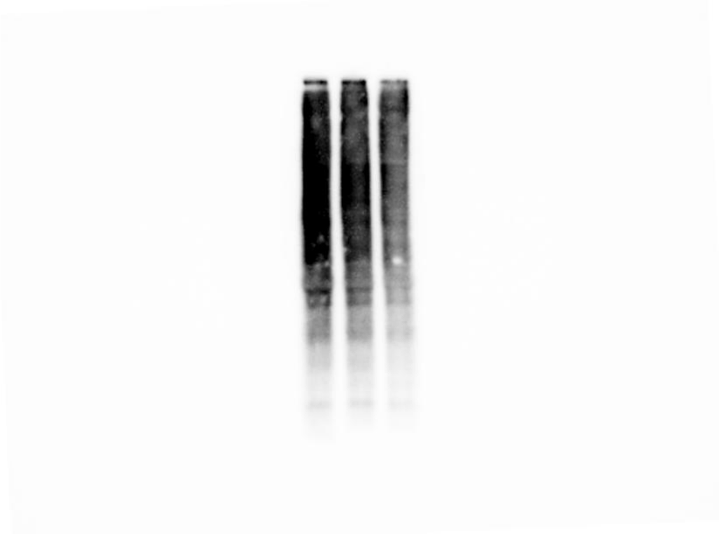

5B Myc

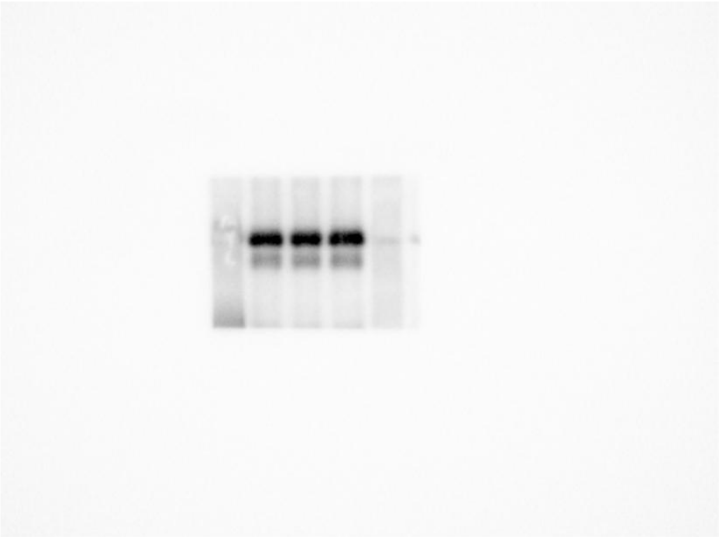

5B Flag

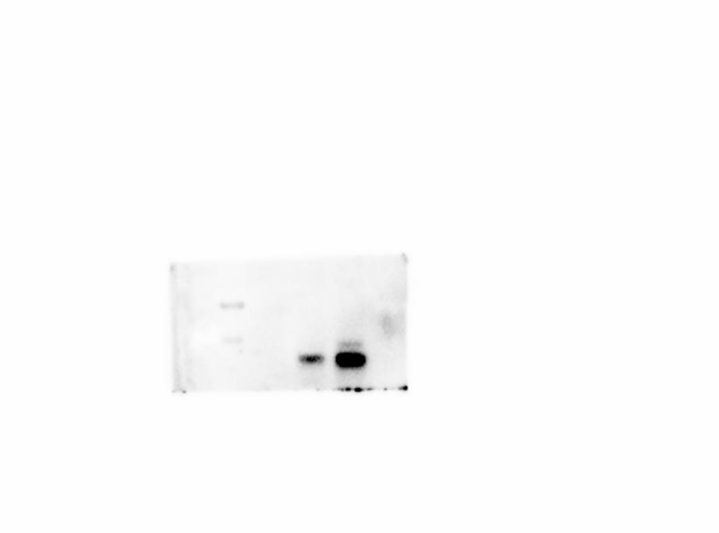

5B GAPDH

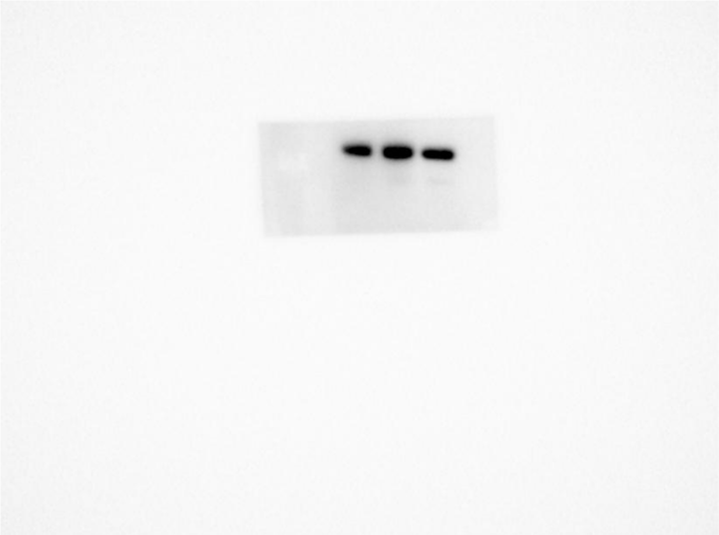

5C HA

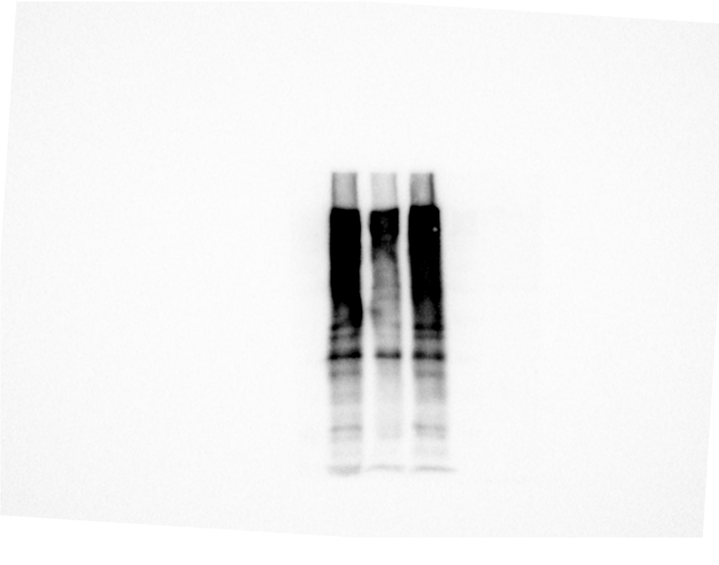

5C Myc

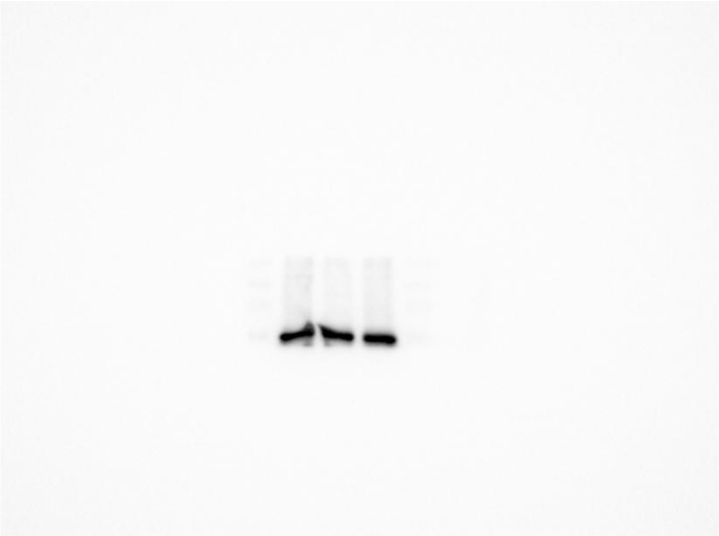

5C Flag

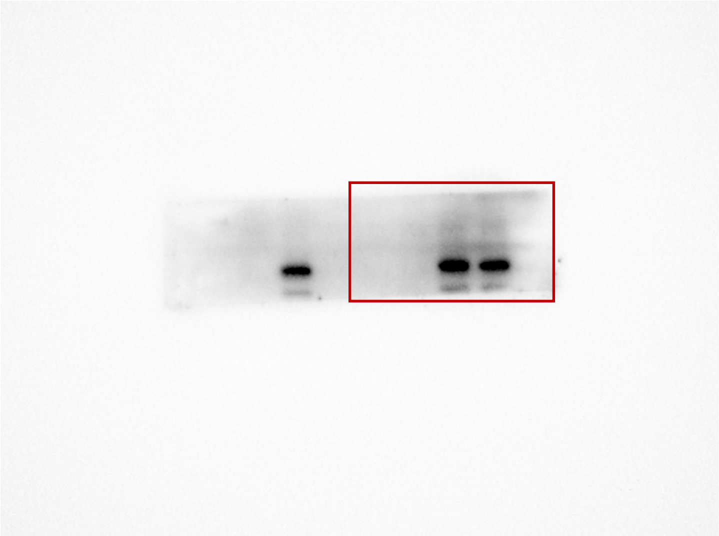

5C GAPDH

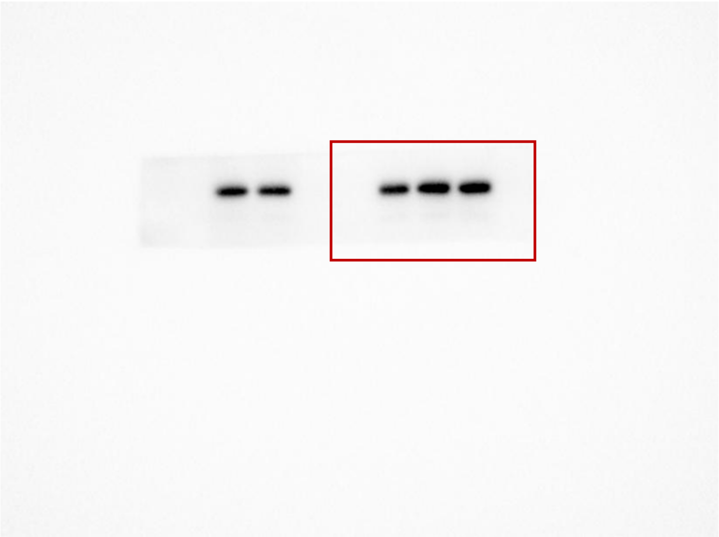

5D HA

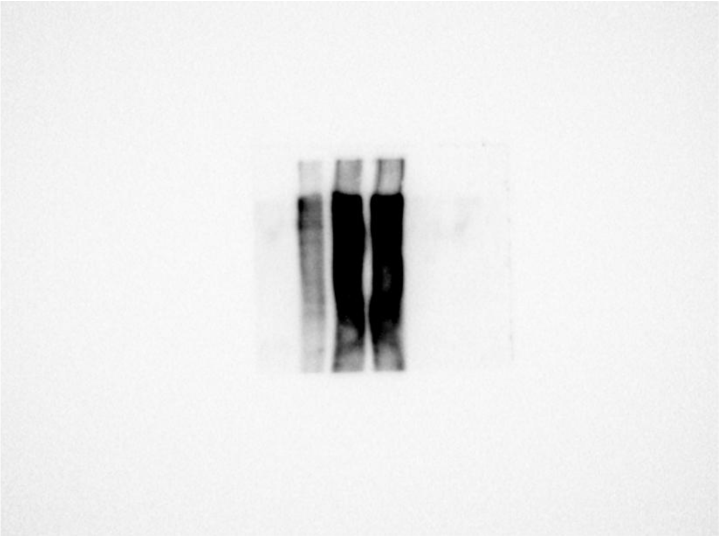

5D Myc

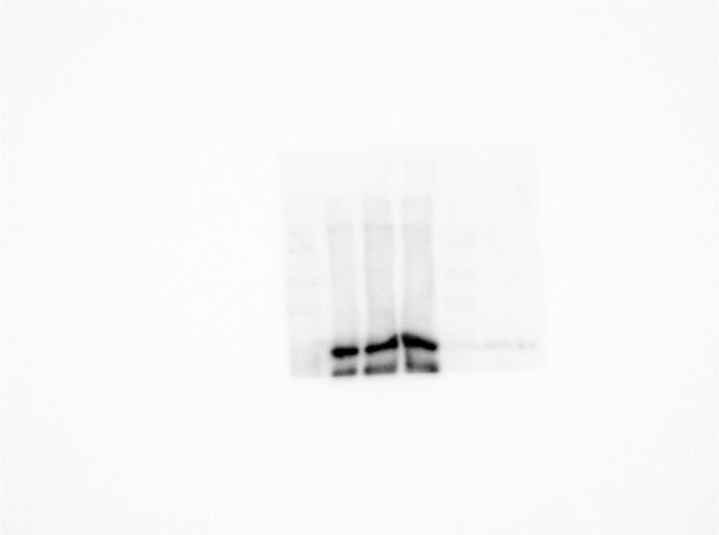

5D YOD1

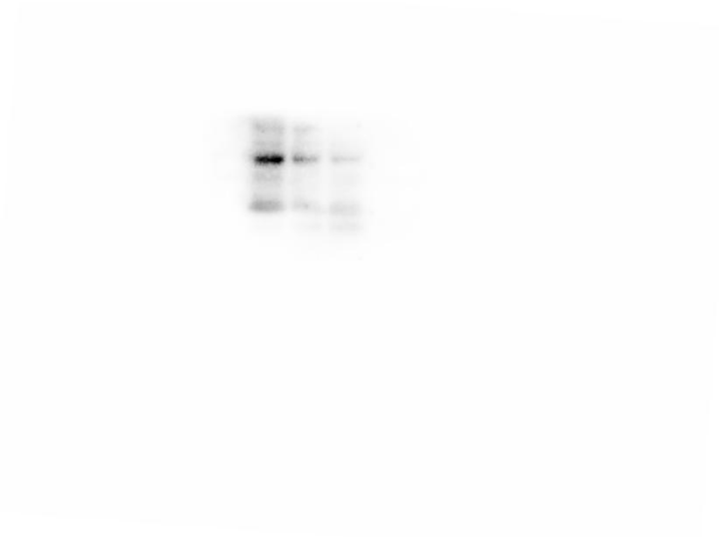

5D GAPDH

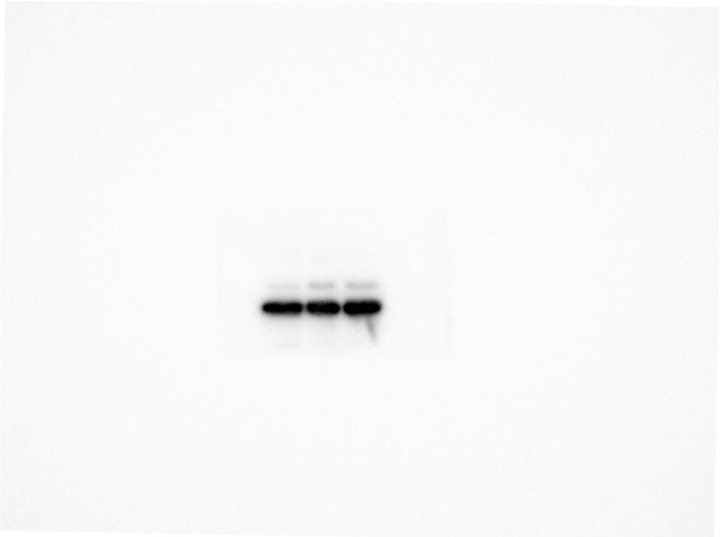

5E HA

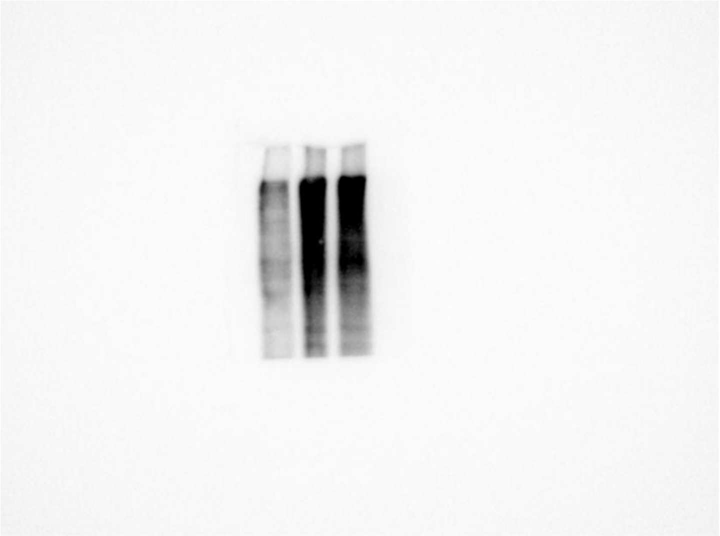

5E Myc

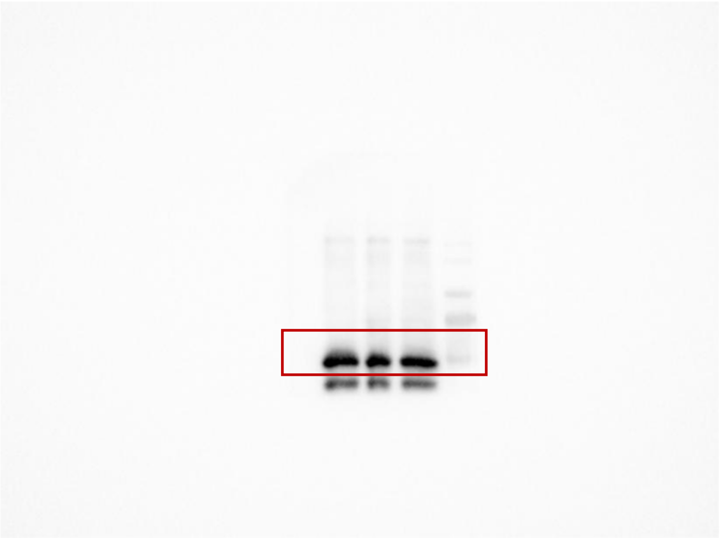

5E YOD1

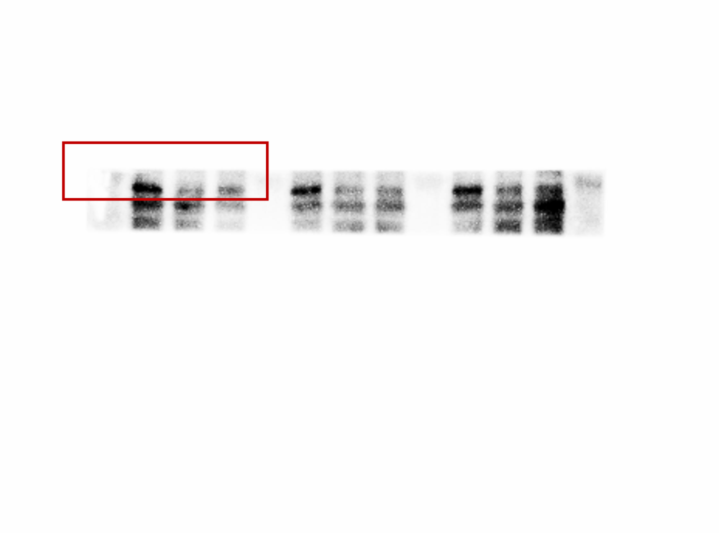

5E GAPDH

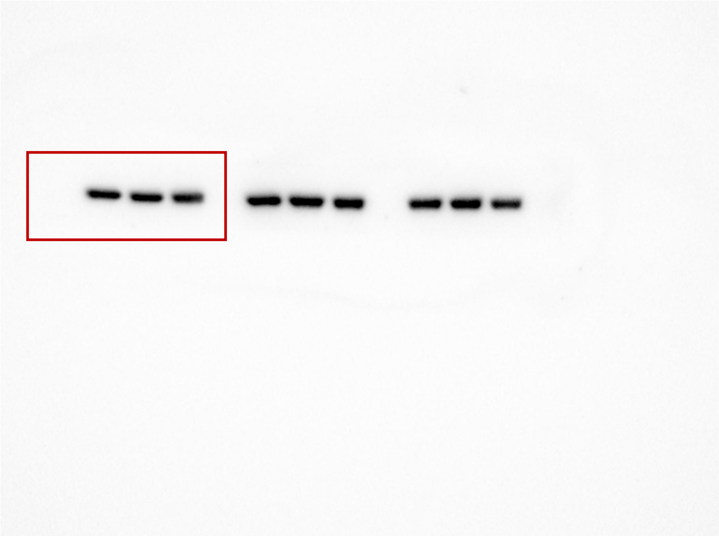

5F HA

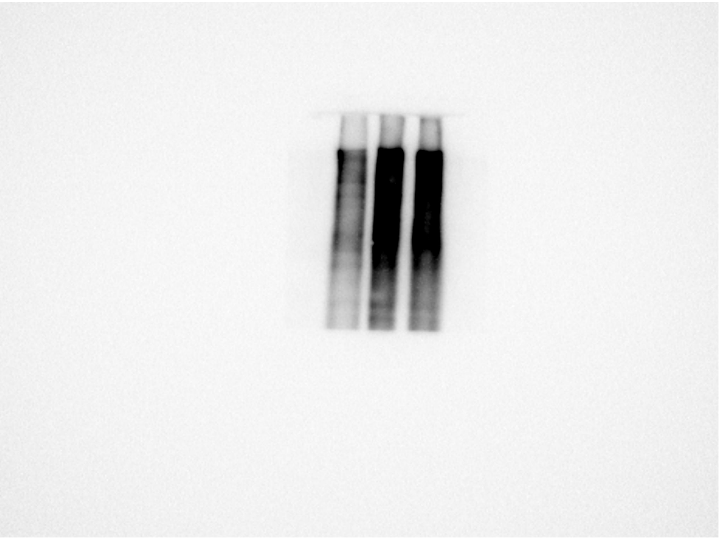

5F Myc

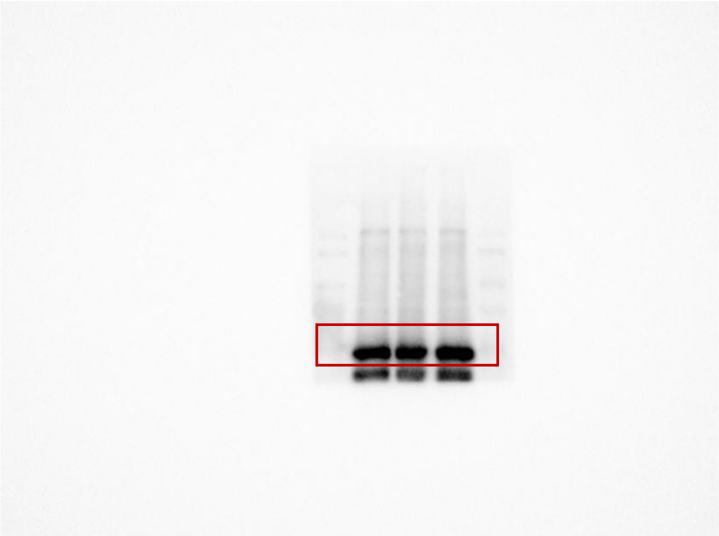

5F YOD1

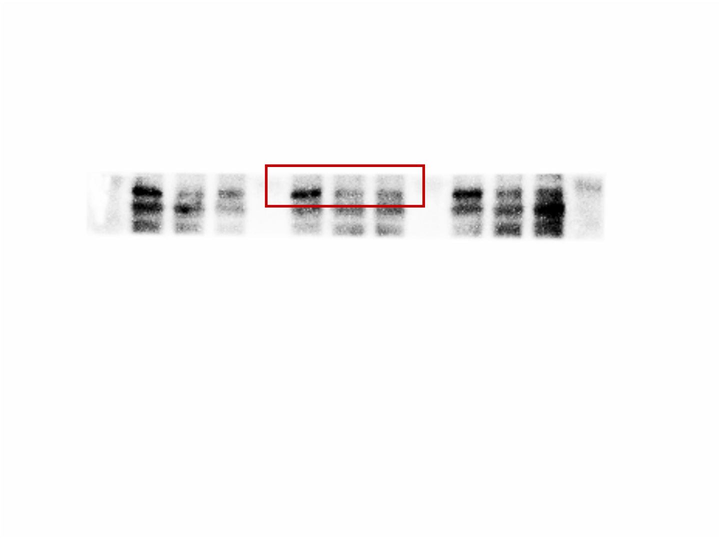

5F GAPDH

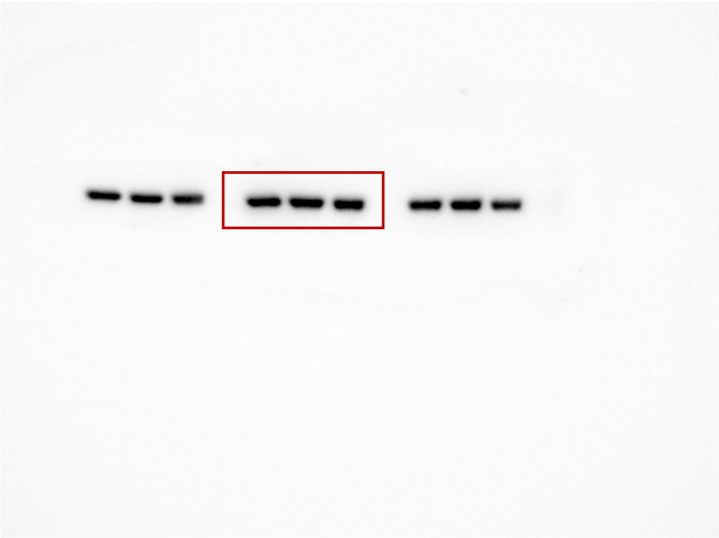

5G HA

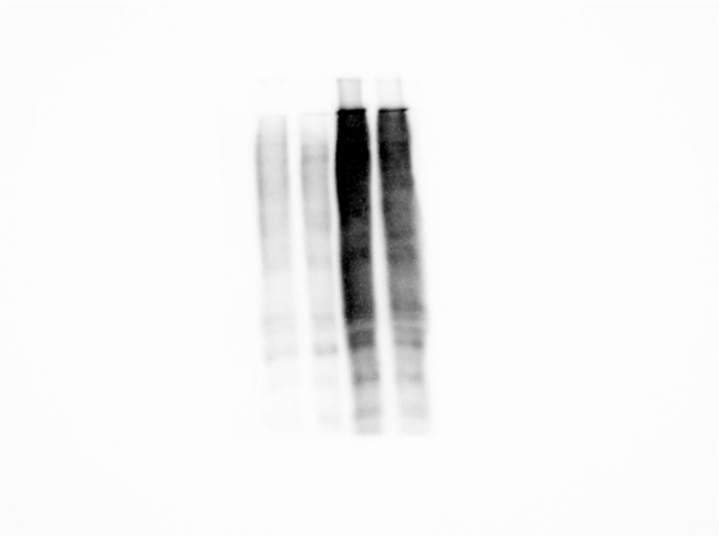

5G Myc

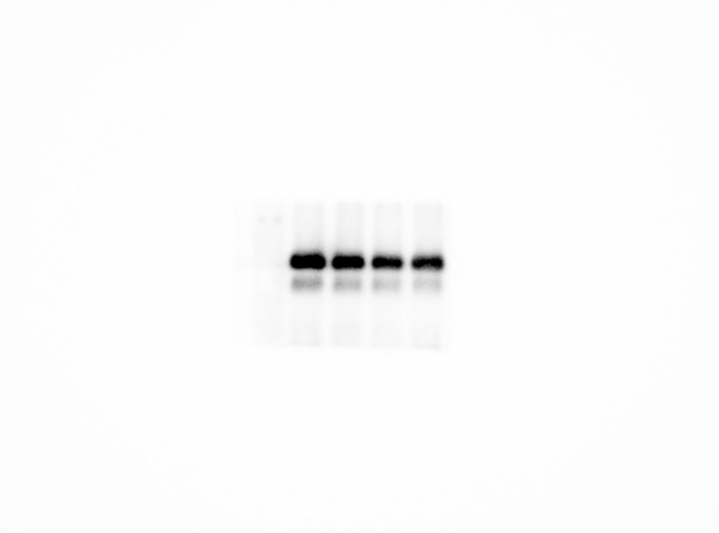

5G Flag

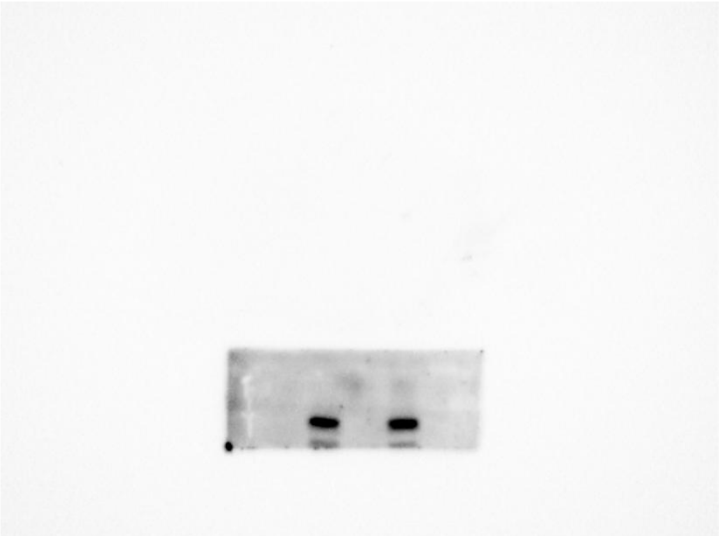

5G GAPDH

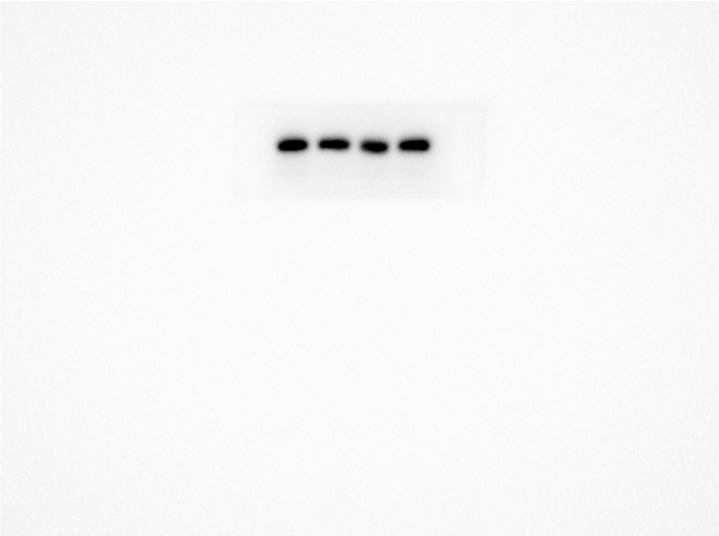

5H HA

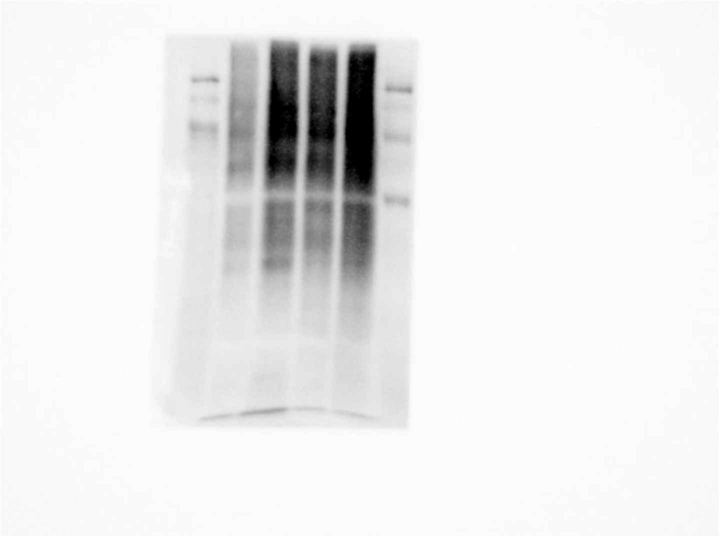

5H Myc

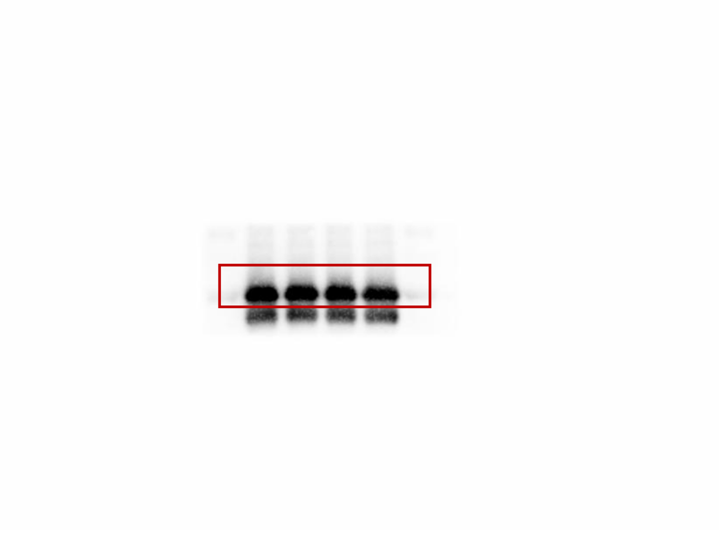

5H YOD1

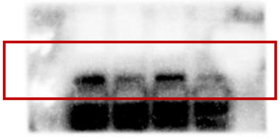

5H GAPDH

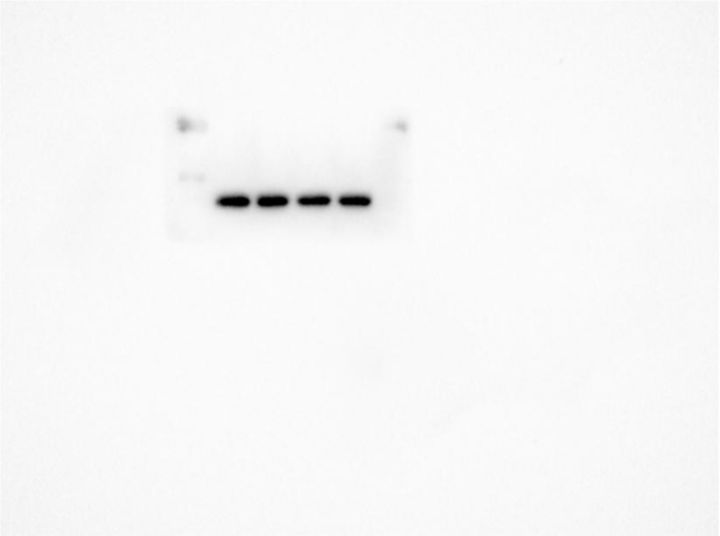

5I HA

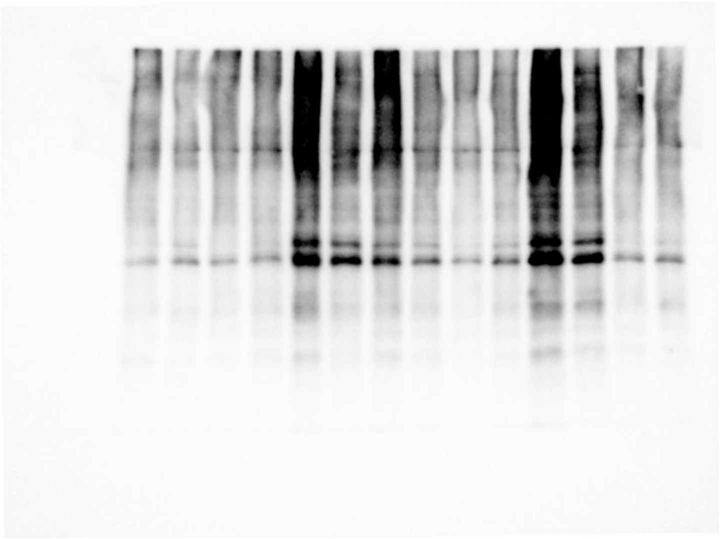

5I Myc

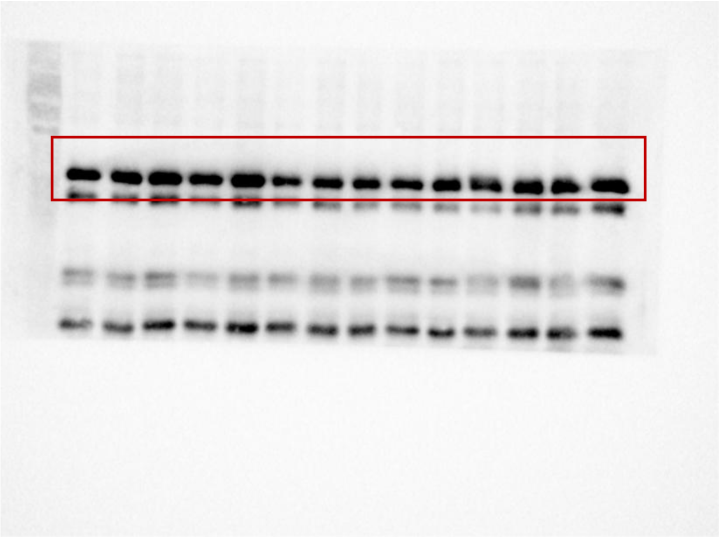

5I Flag

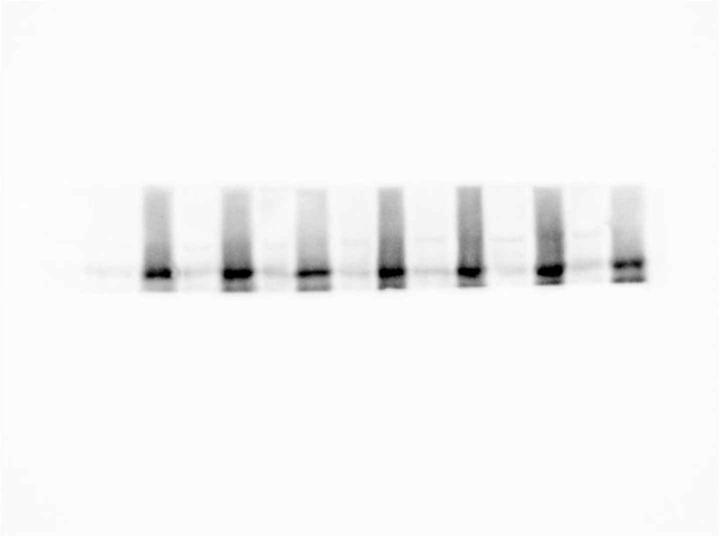

5I GAPDH

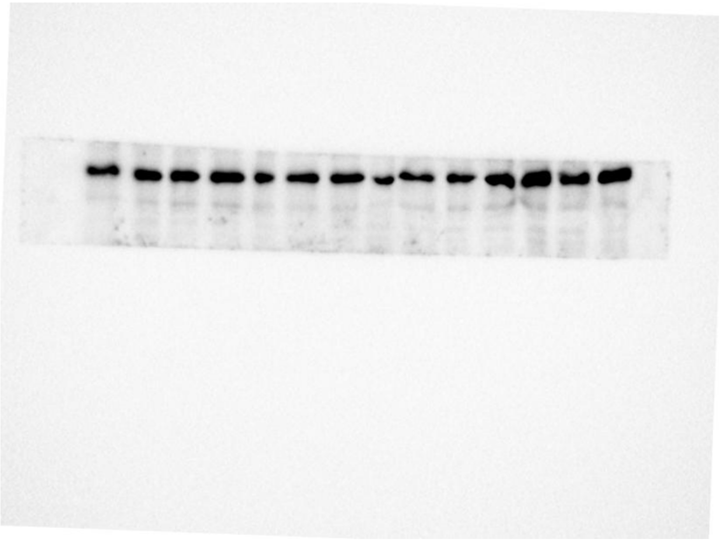

5K HA

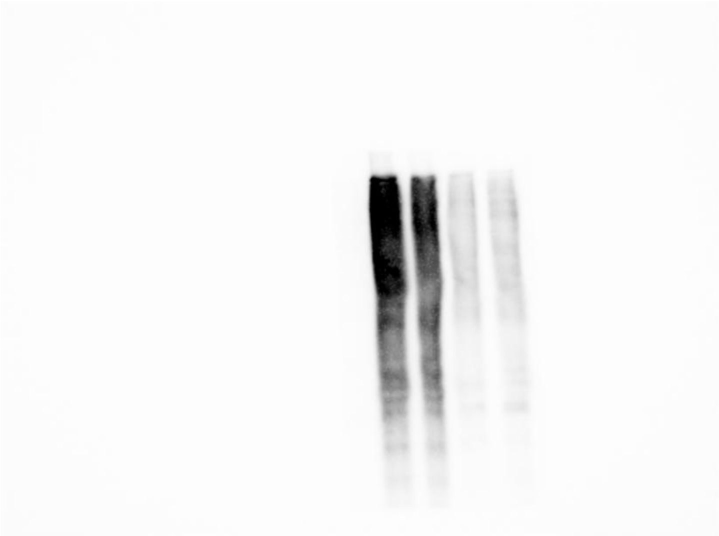

5K Myc

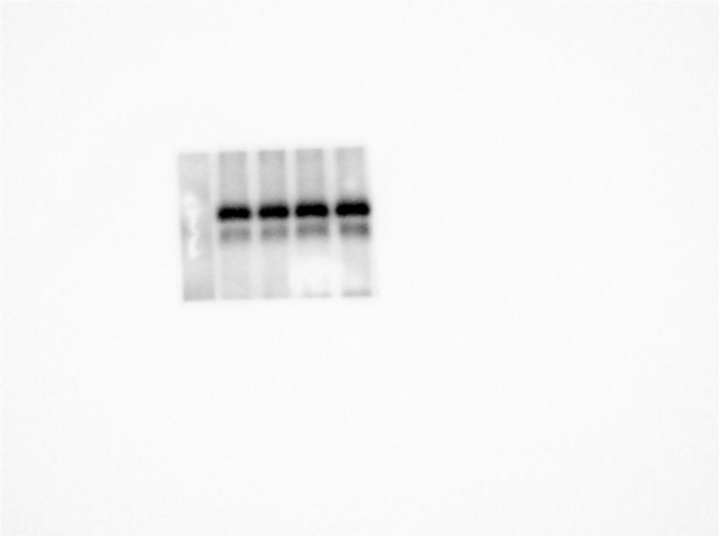

5K Flag

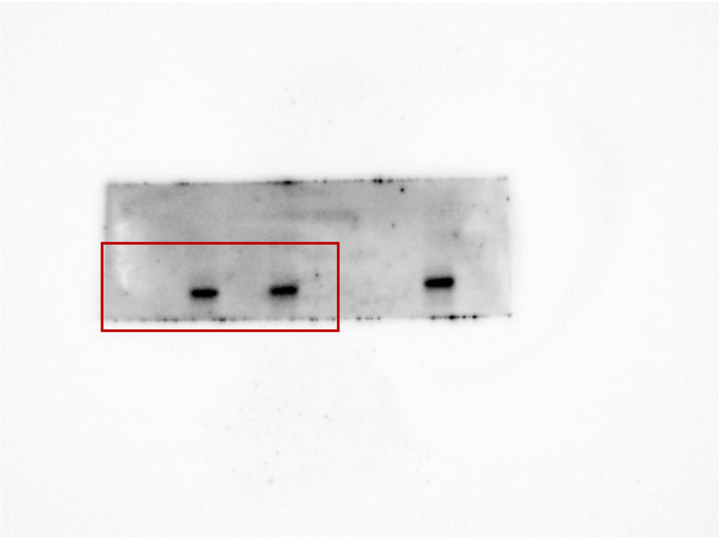

5K GAPDH

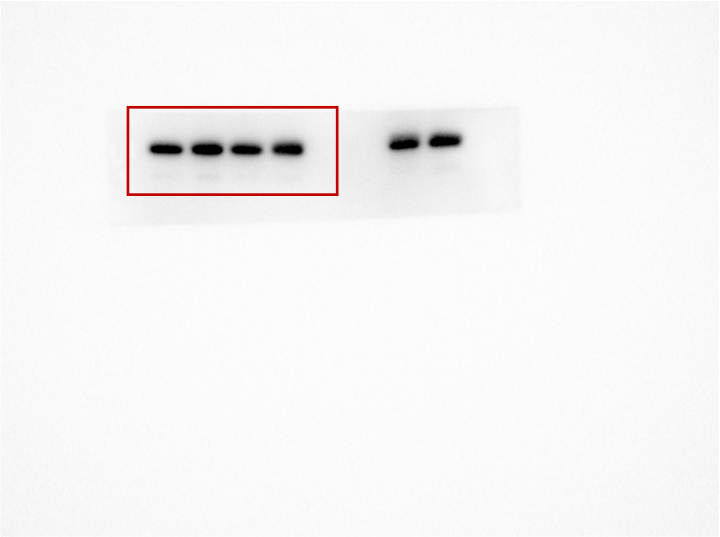

Figure 6

6A A498 ZNF24

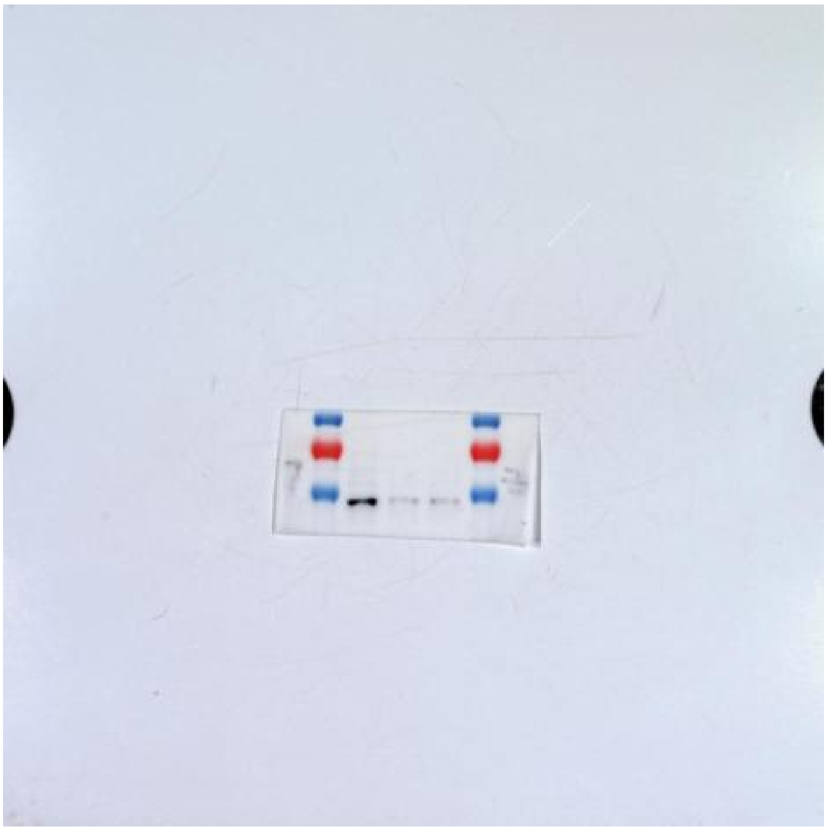

6A A498 YOD1

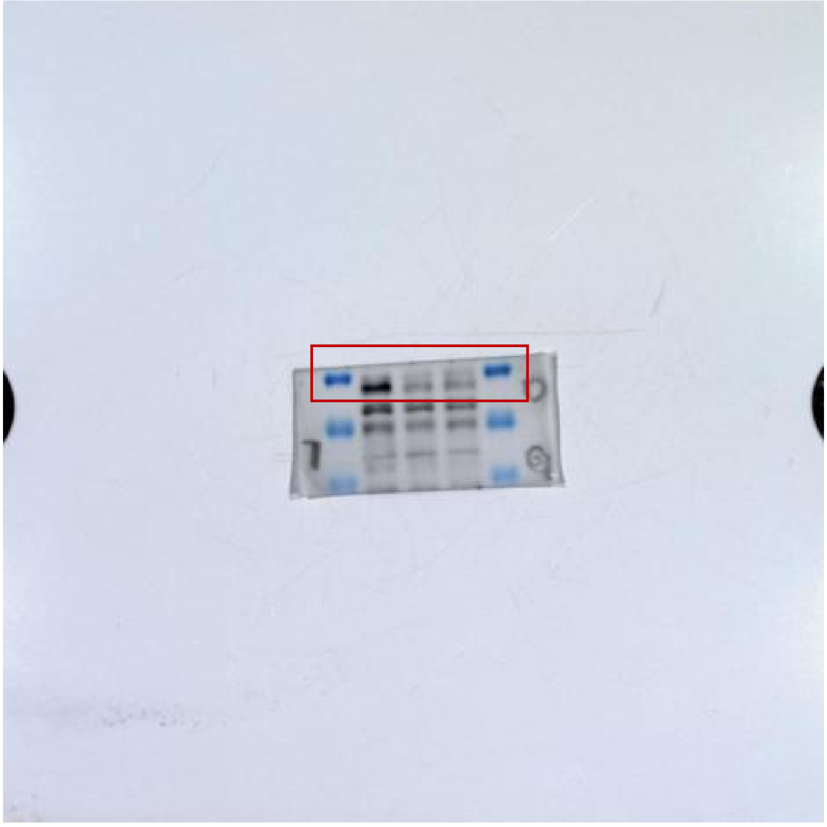

6A A498 GAPDH

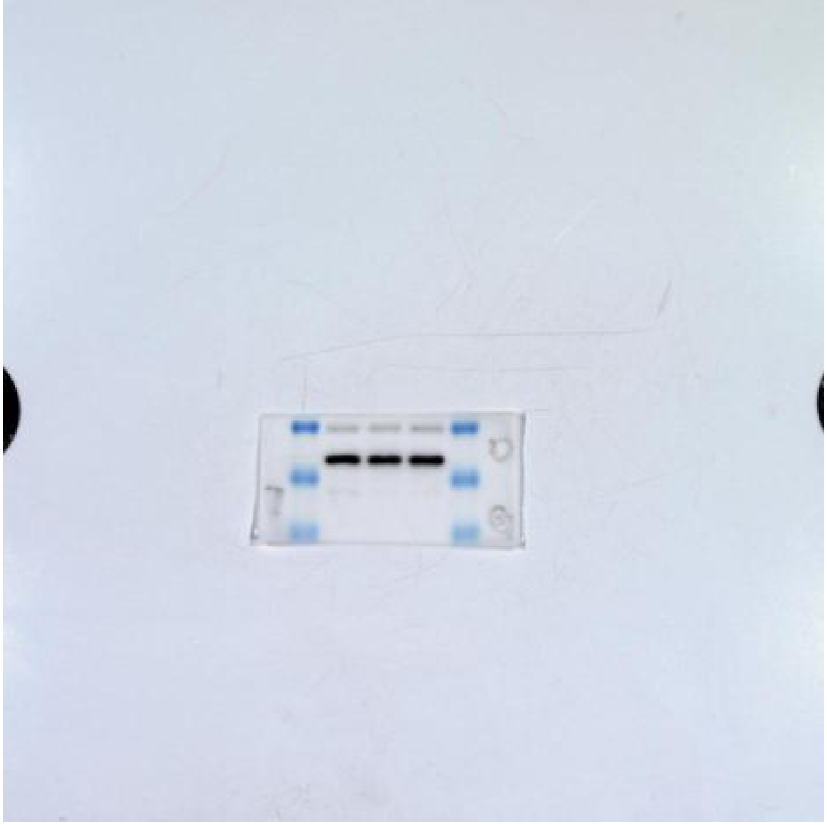

6A 786-O ZNF24

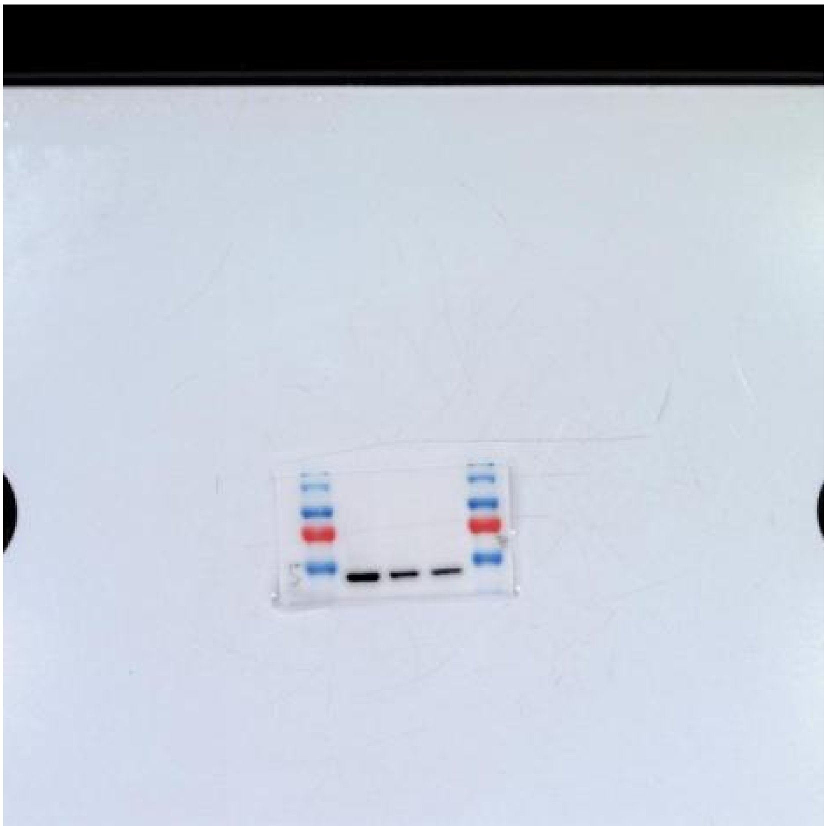

6A 786-O YOD1

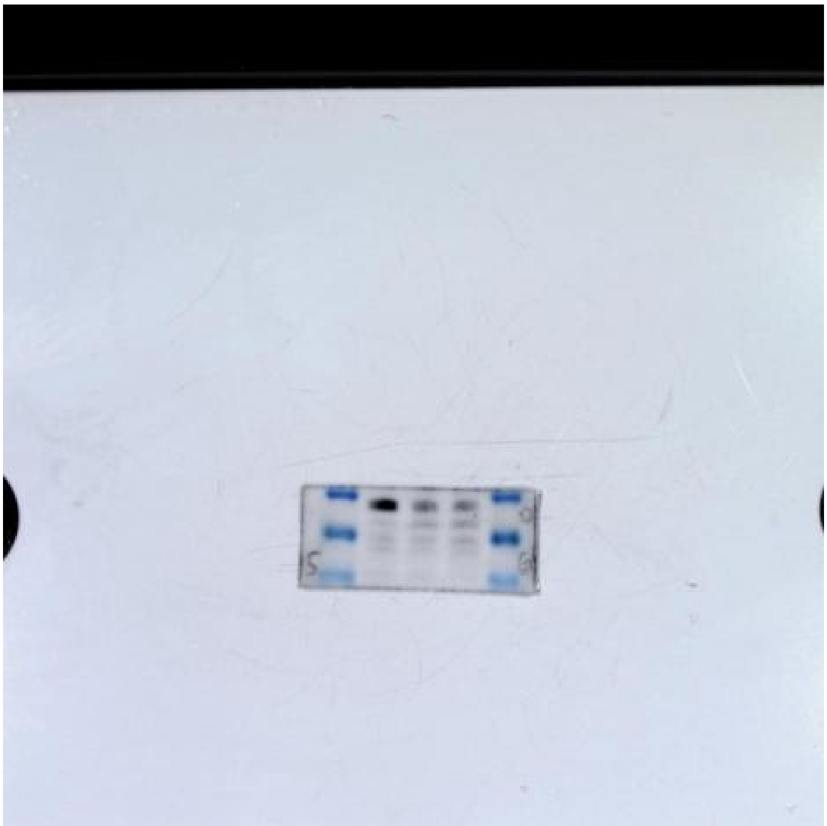

6A　786-O　GAPDH

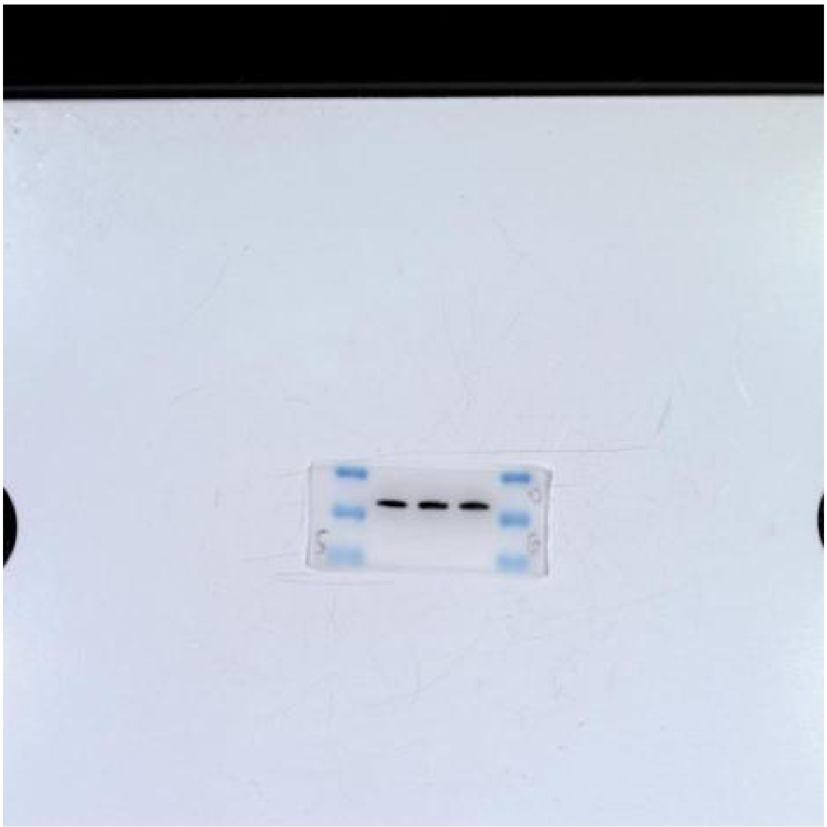

6B　A498　ZNF24

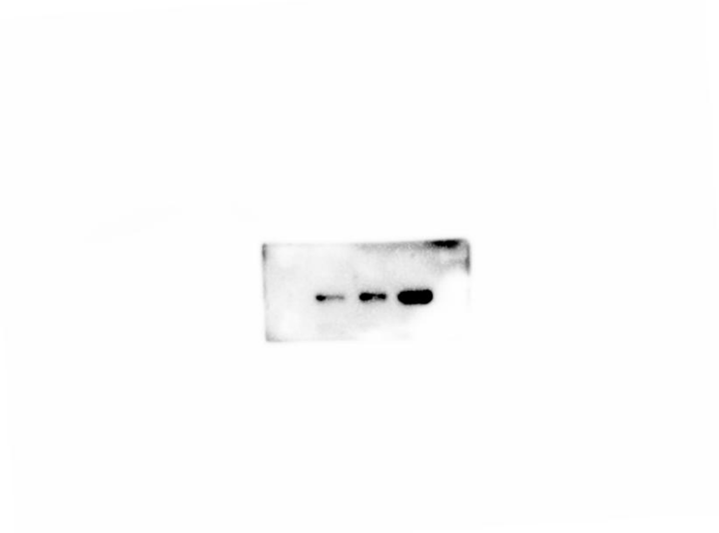

6B A498 Flag

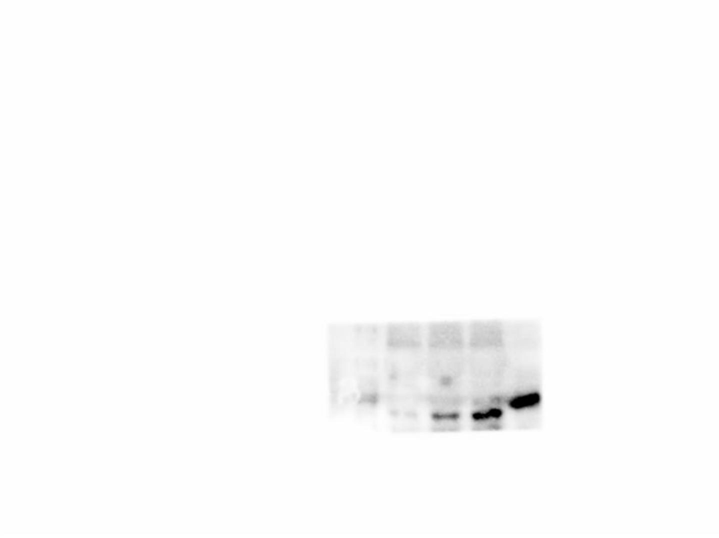

6B A498 GAPDH

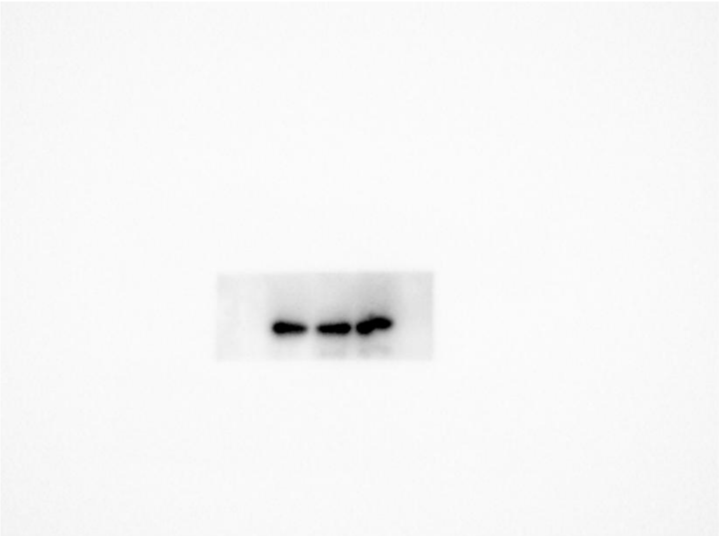

6B 786-O ZNF24

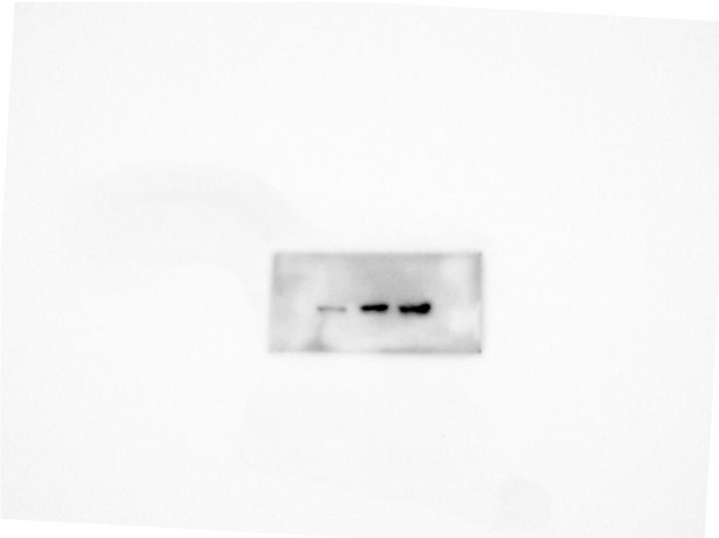

6B 786-O Flag

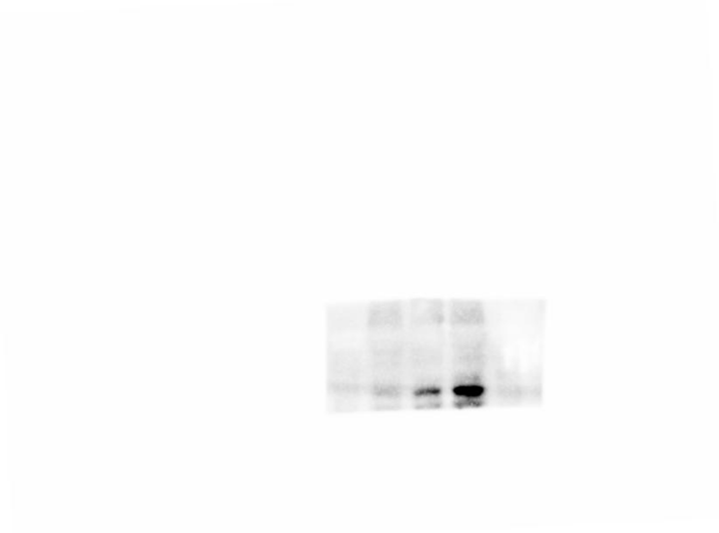

6B 786-O GAPDH

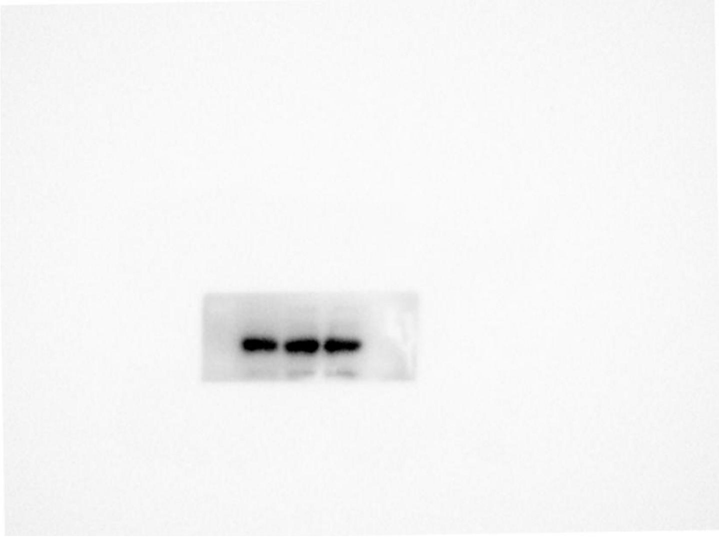

6C A498 ZNF24

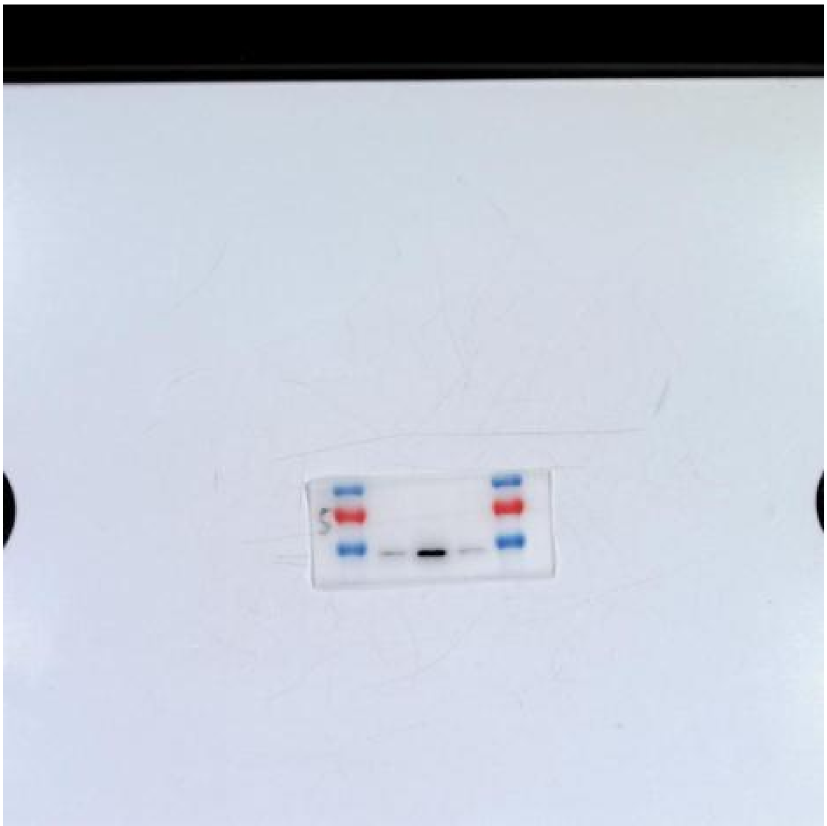
6C A498 FLAG

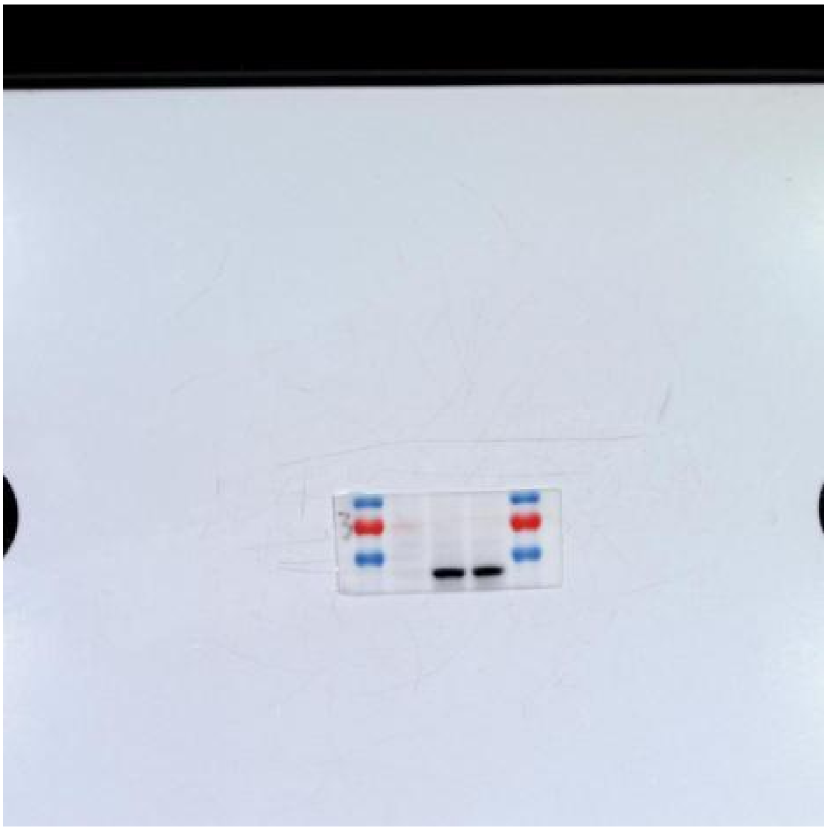

6C A498 GAPDH

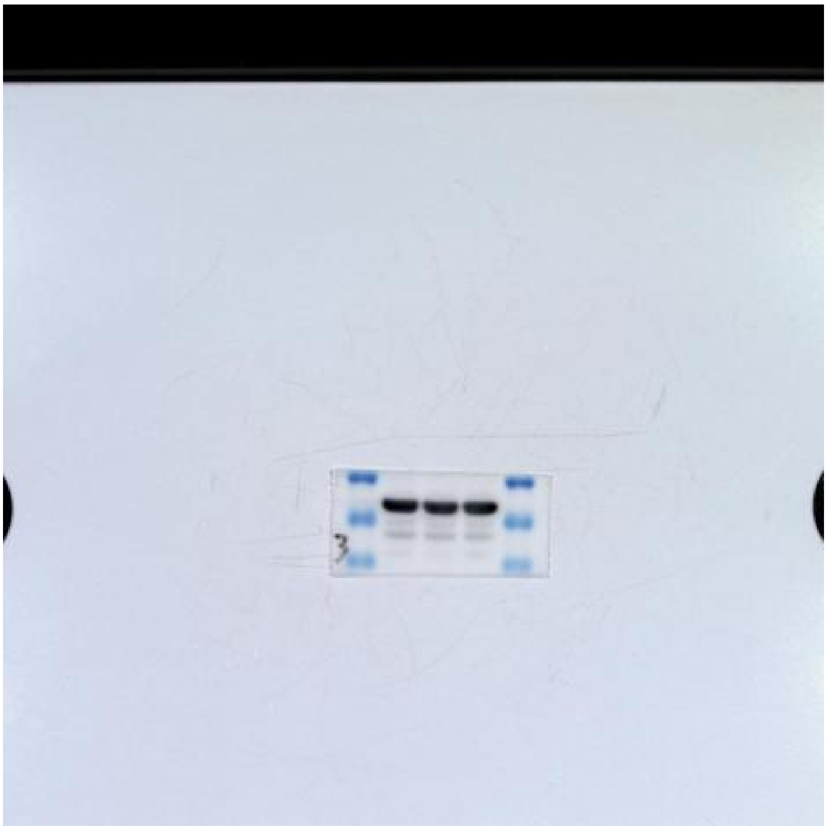

6C 786-O ZNF24

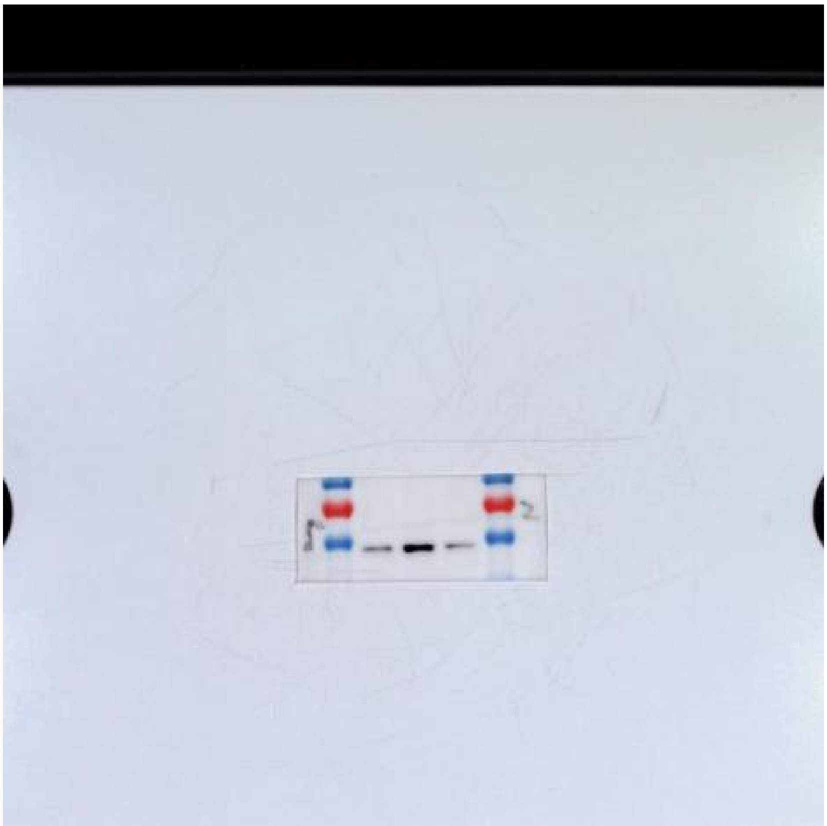

6C 786-O FLAG

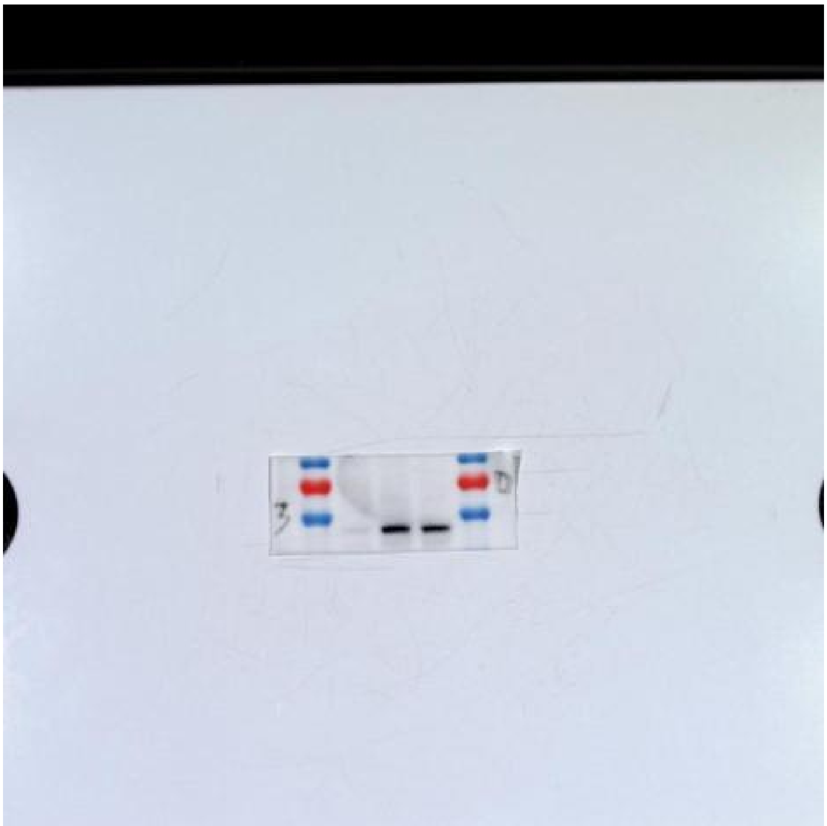

6C 786-O GAPDH

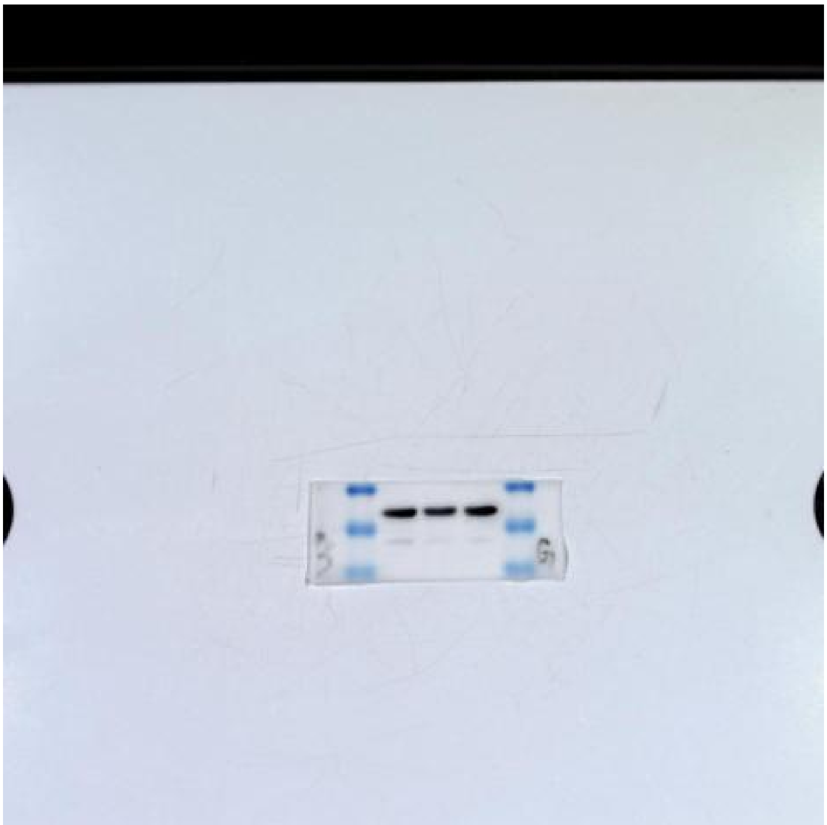

6E A498 ZNF24

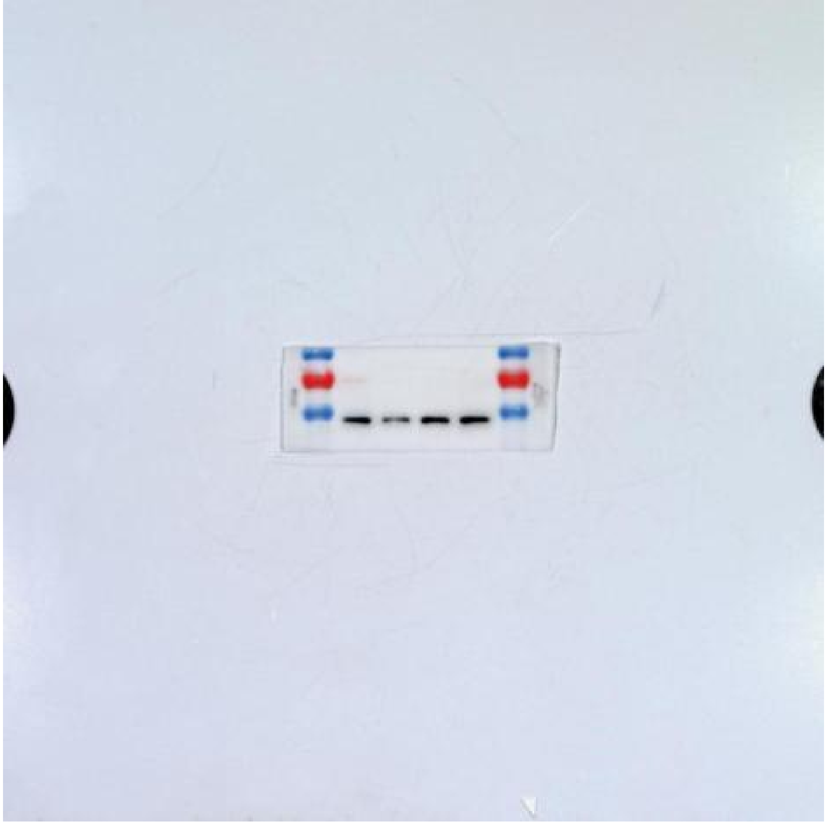

6E A498 YOD1

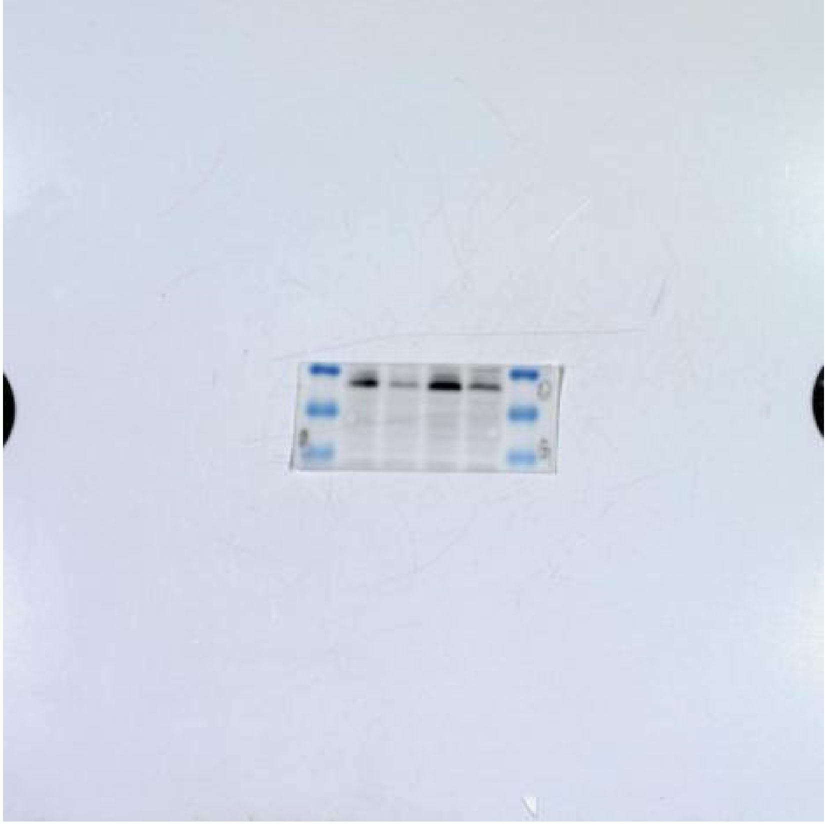

6E A498 GAPDH

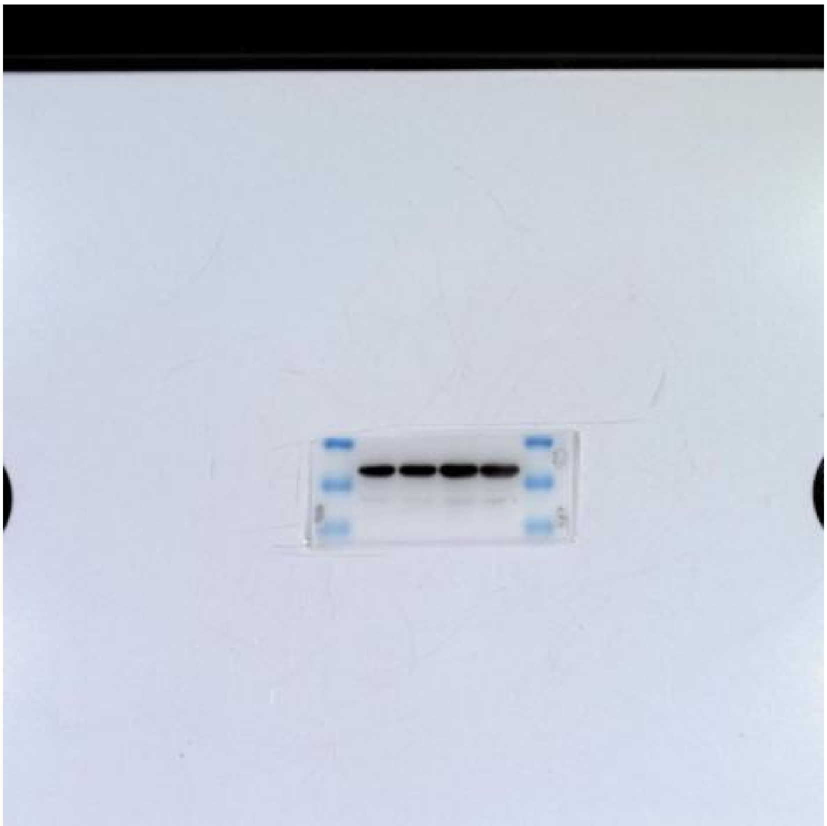

6E　786-O　ZNF24

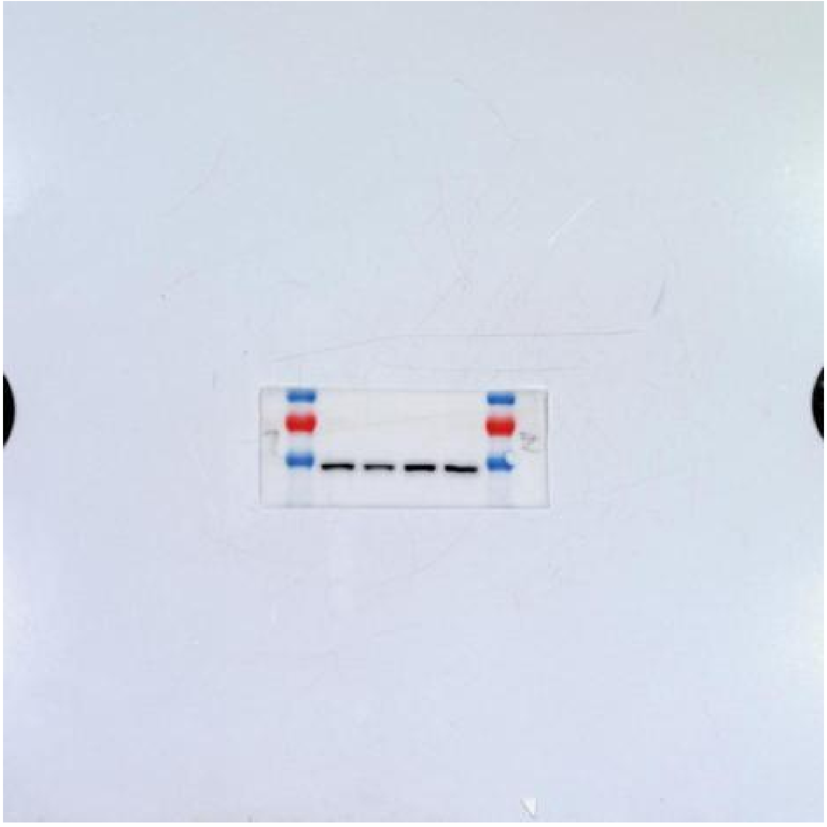

6E　786-O　YOD1

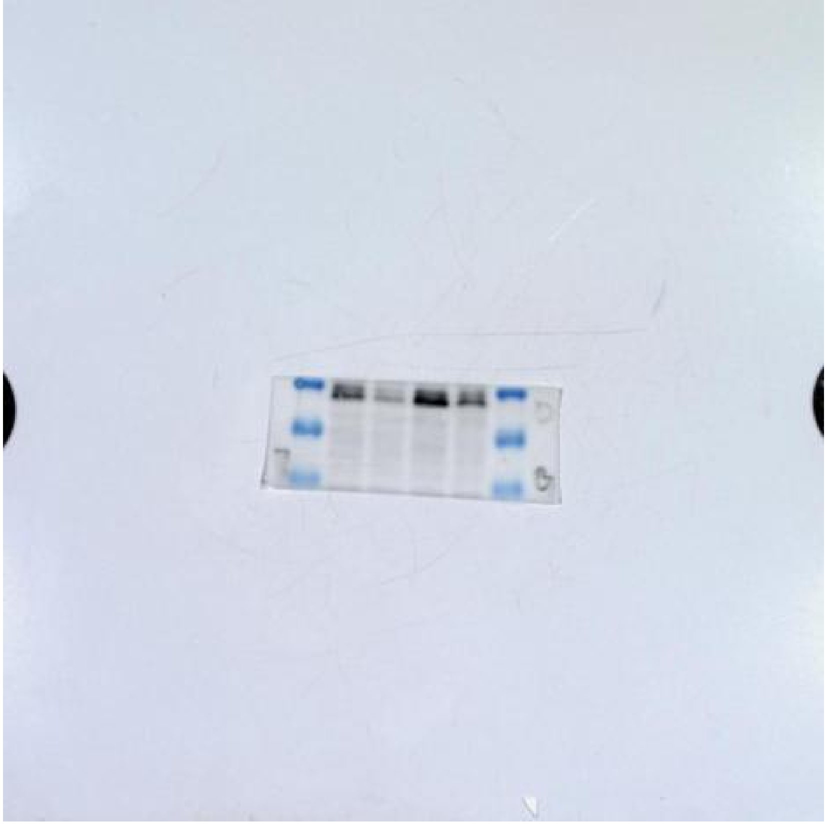

6E 786-O GAPDH

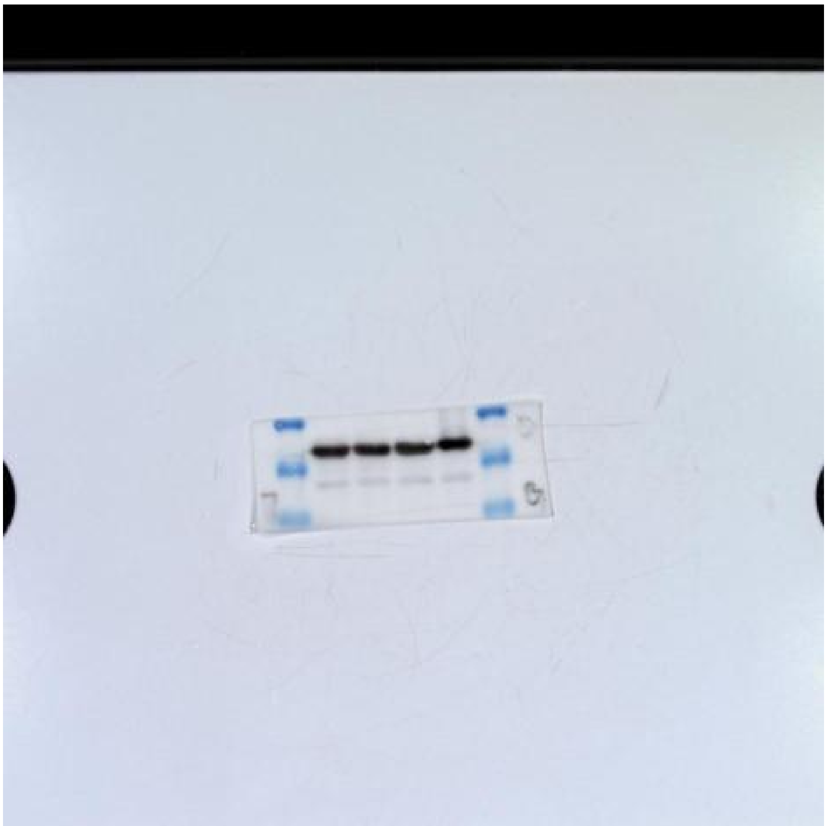

6F A498 ZNF24

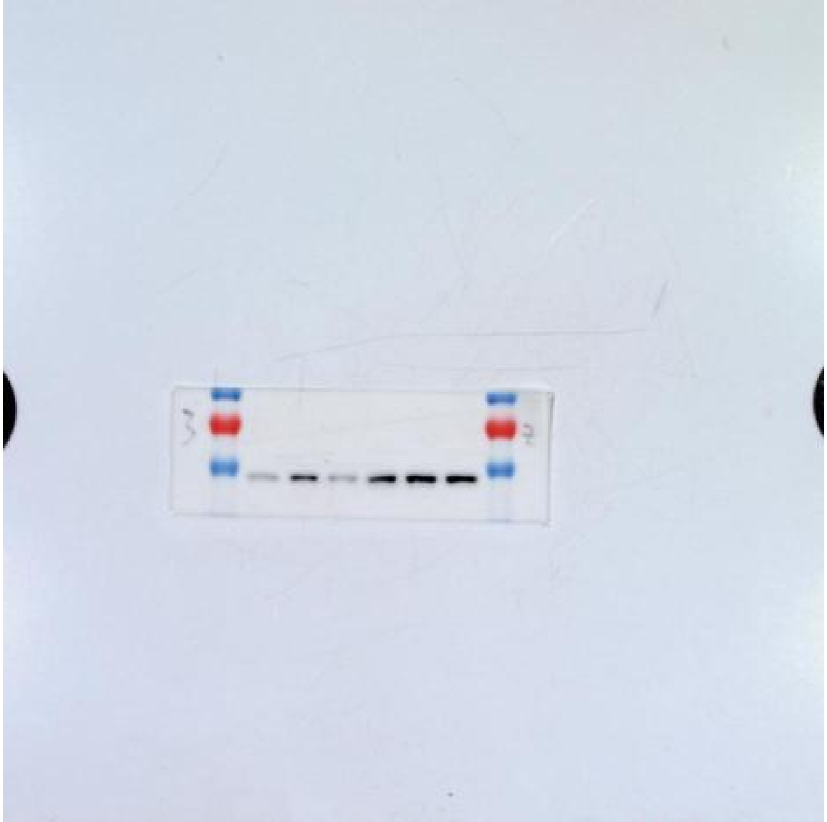

6F A498 FLAG

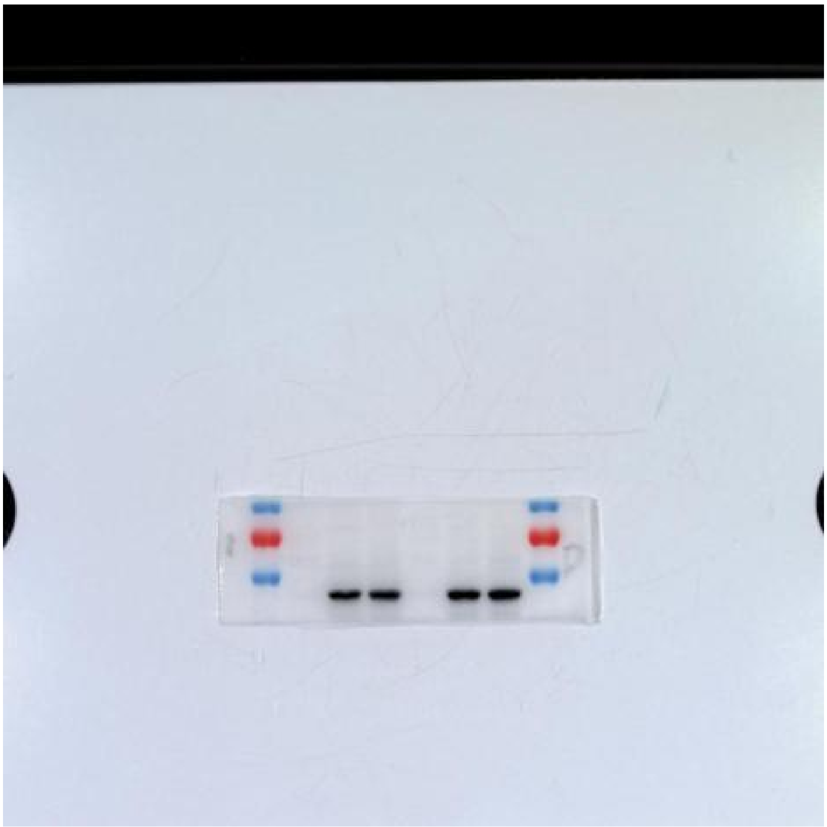

6F A498 GAPDH

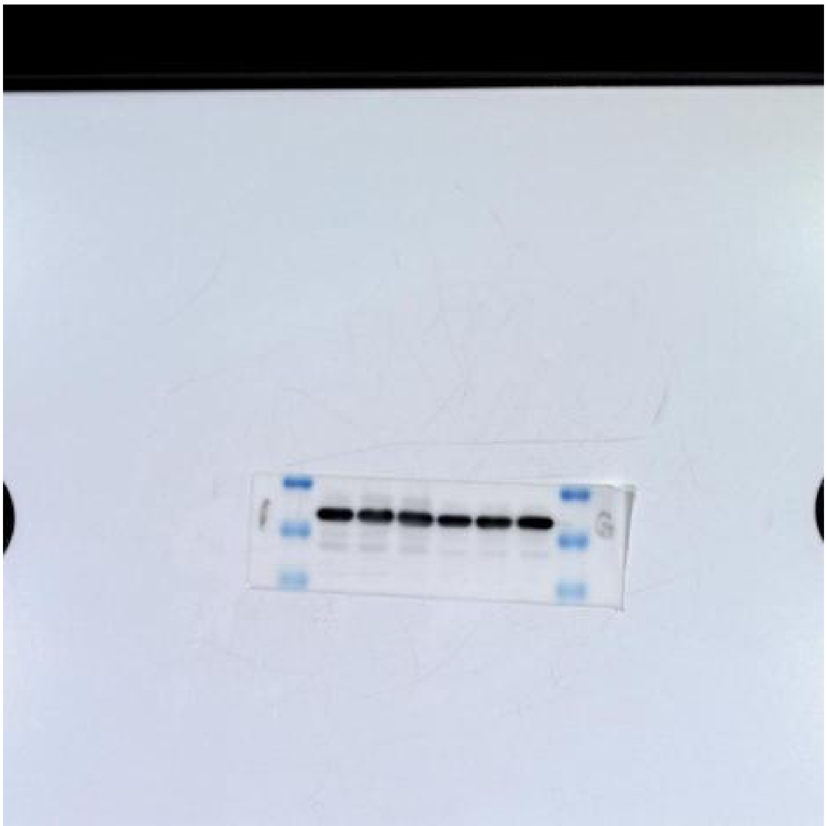

6F 786-O ZNF24

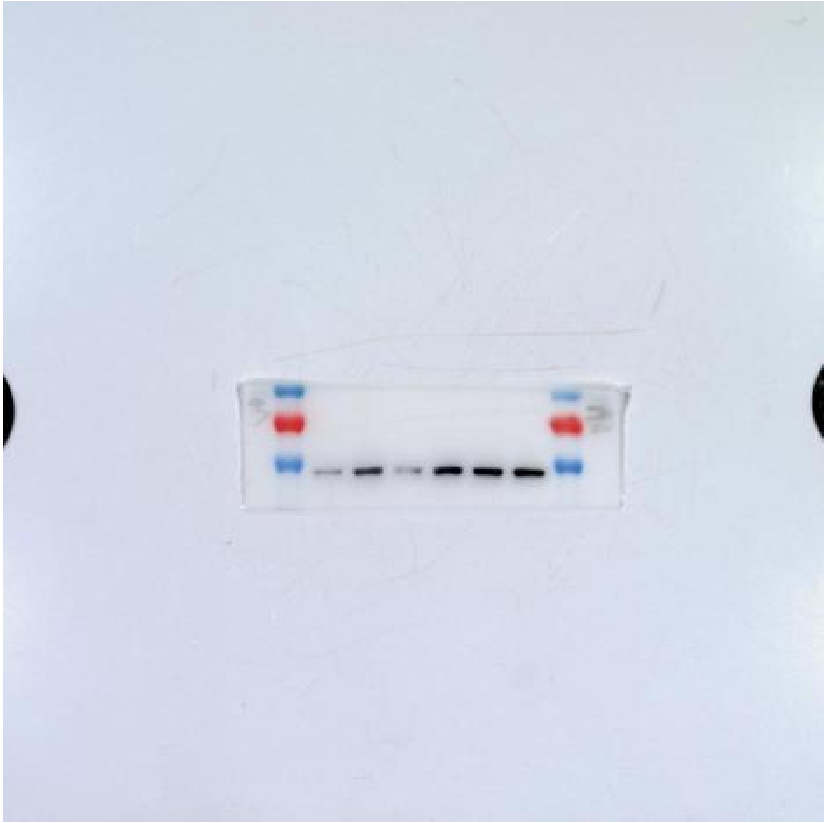

6F 786-O FLAG

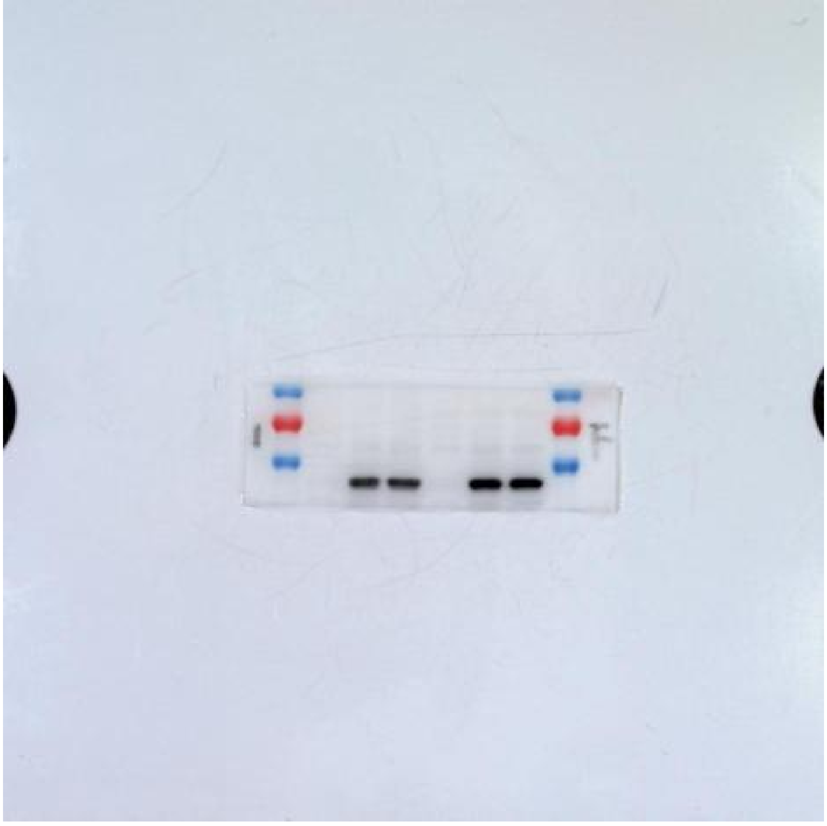

6F 786-O GAPDH

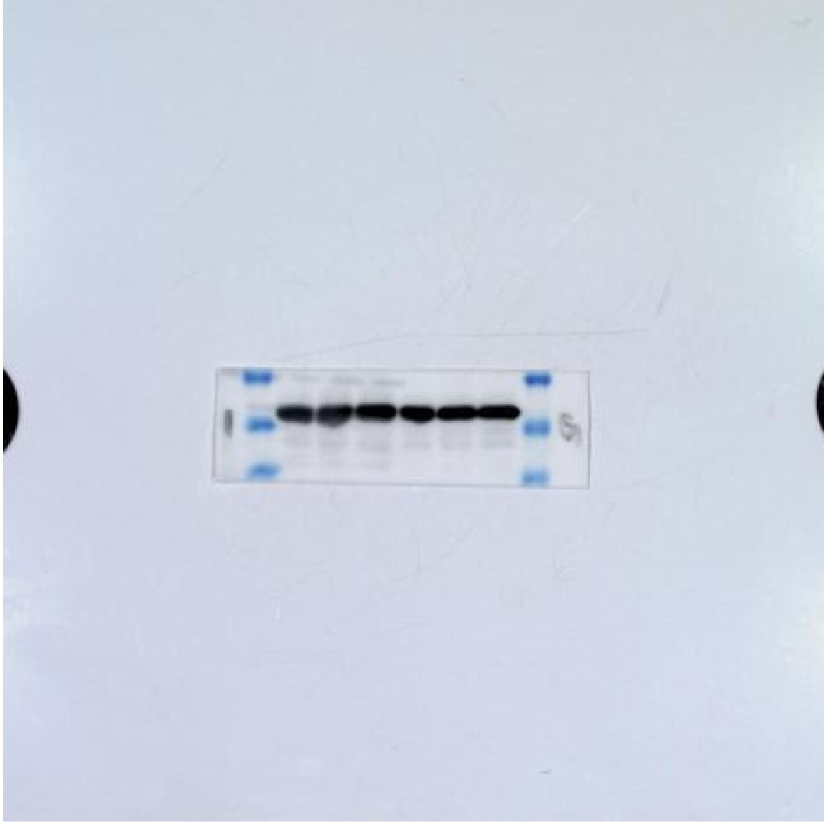

6G ZNF24

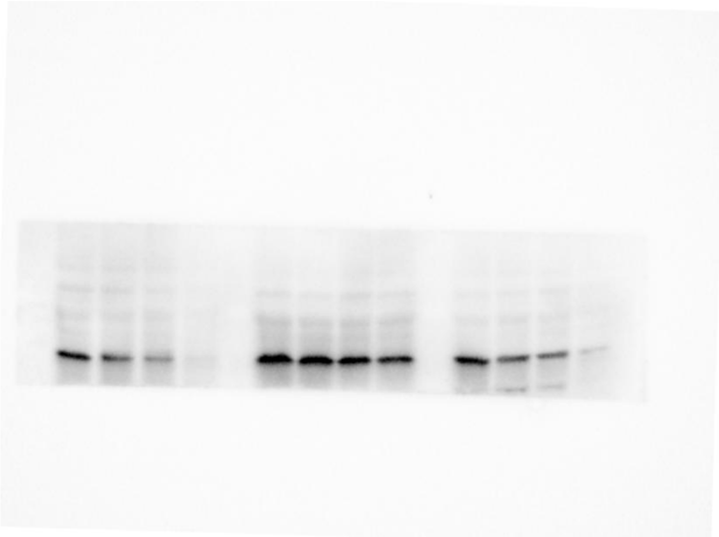

6G Flag

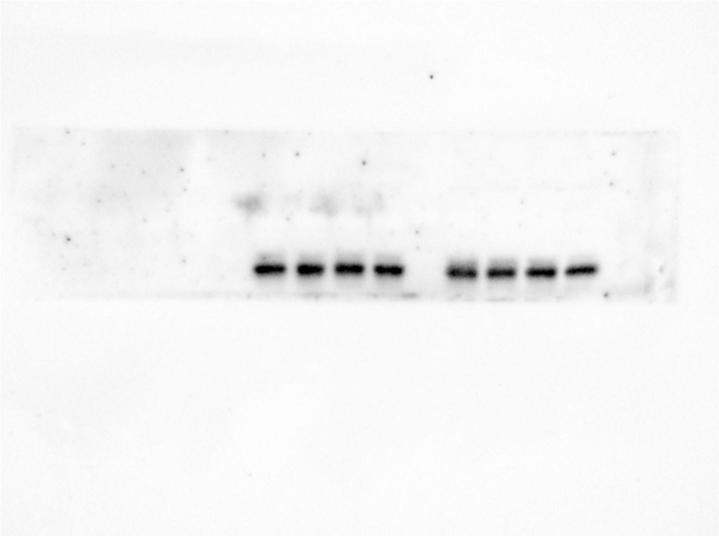

6G GAPDH

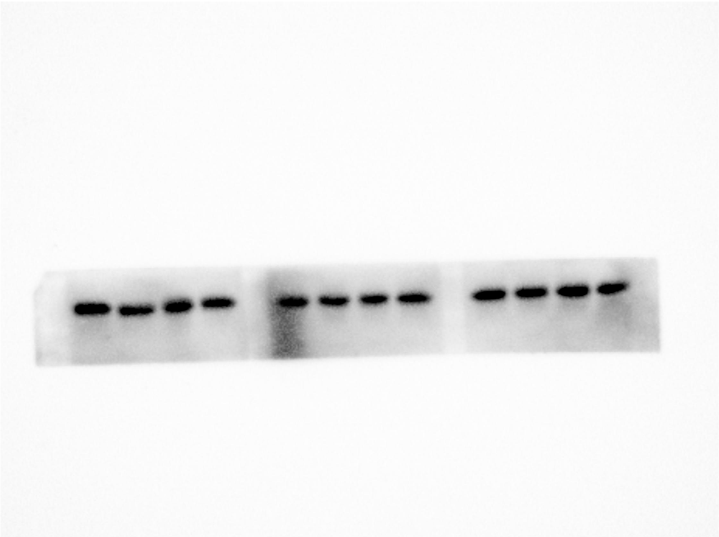

6H ZNF24

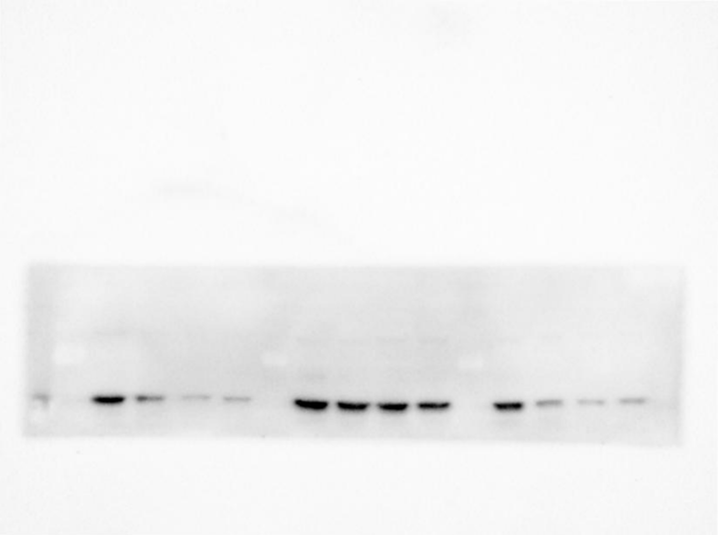

6H Flag

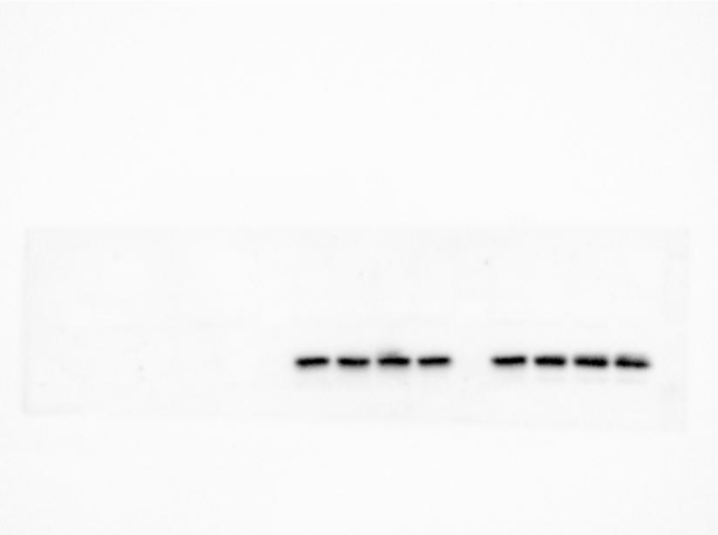

6H GAPDH

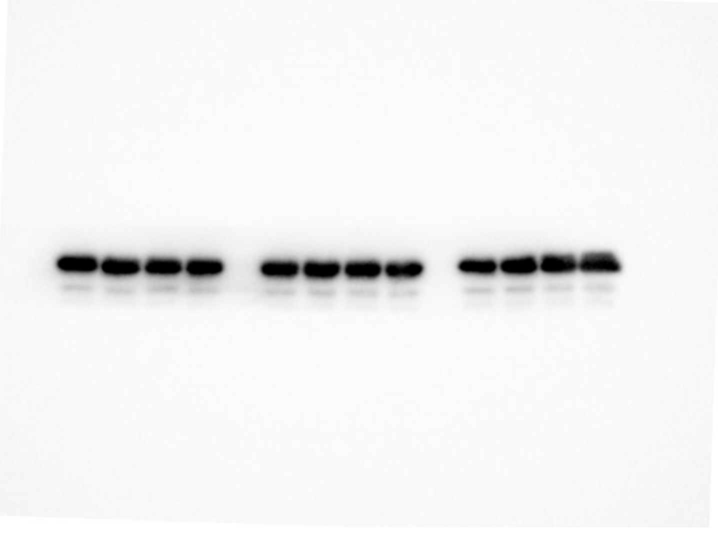

6I ZNF24

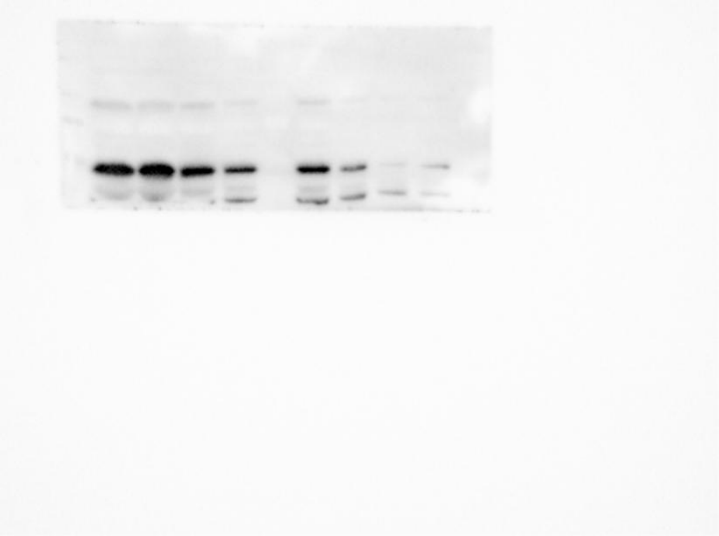

6I YOD1

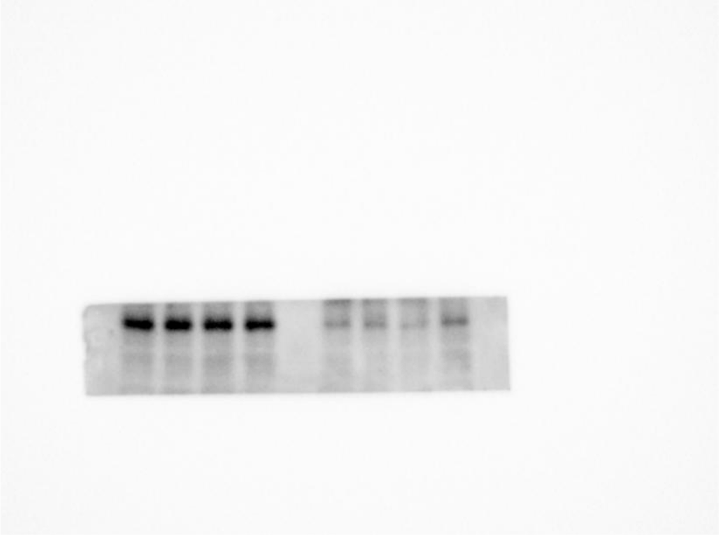

6I GAPDH

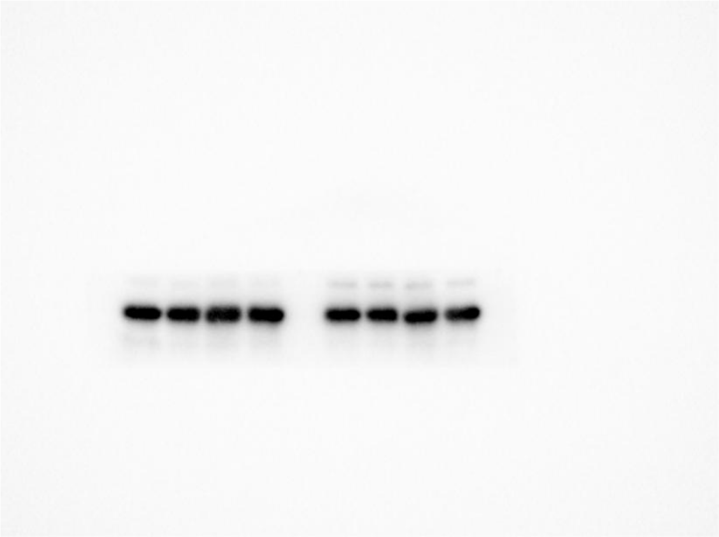

6J ZNF24

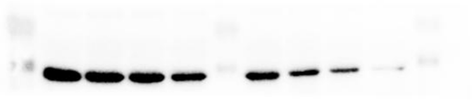

6J YOD1

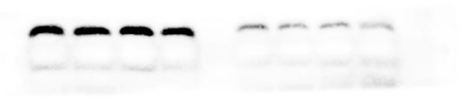

6J GAPDH

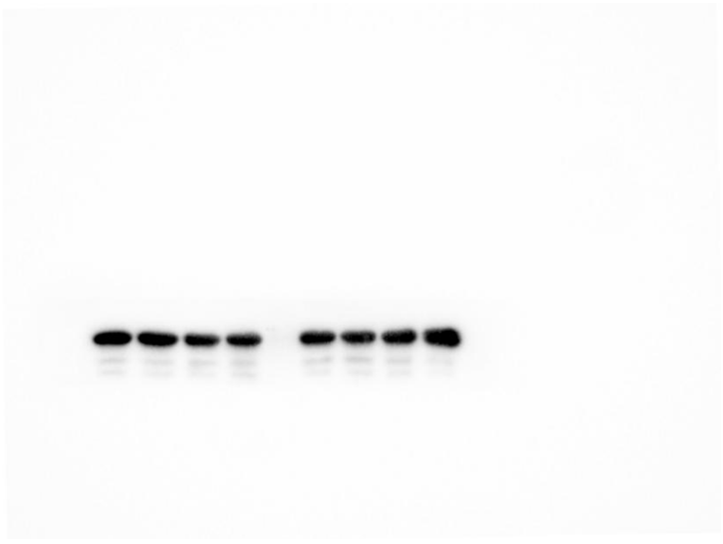

Figure 7

7E A498 ZNF24

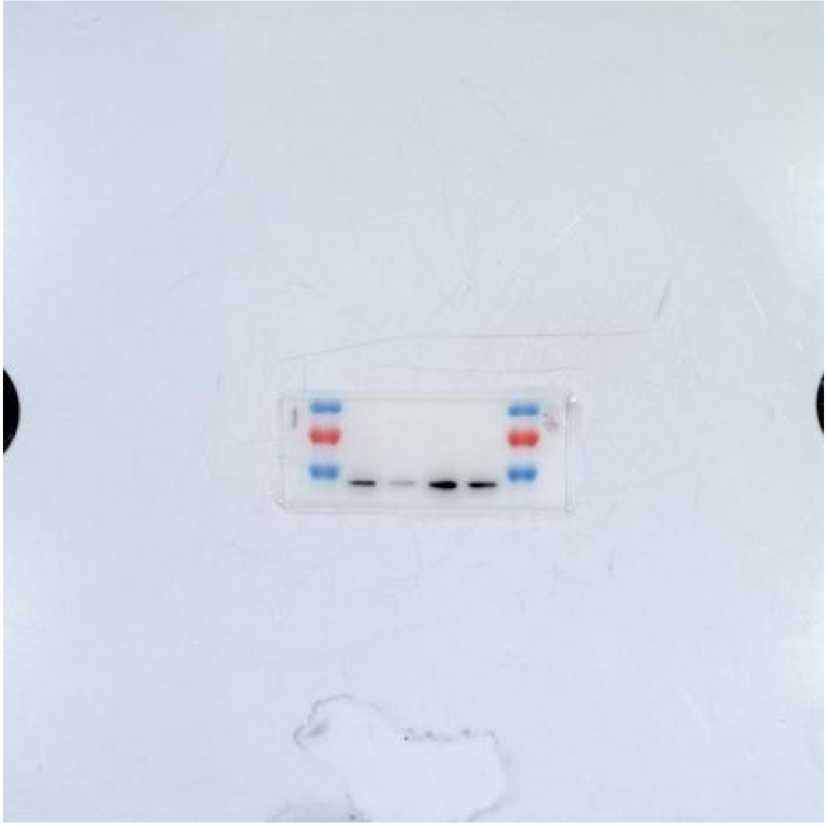

7E A498 YOD1

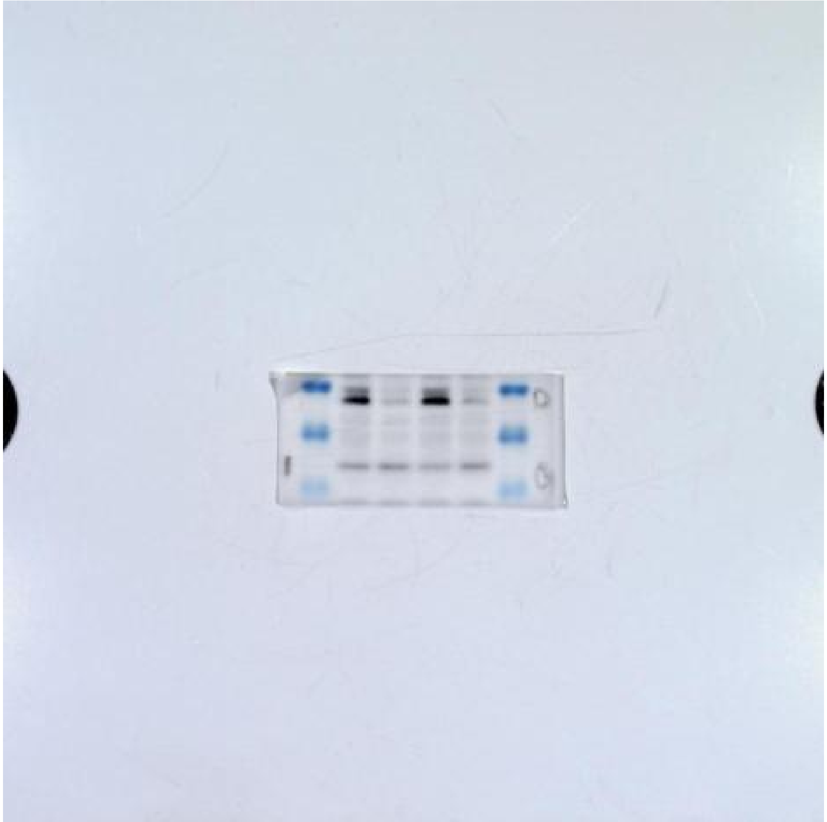

7E A498 VEGFA

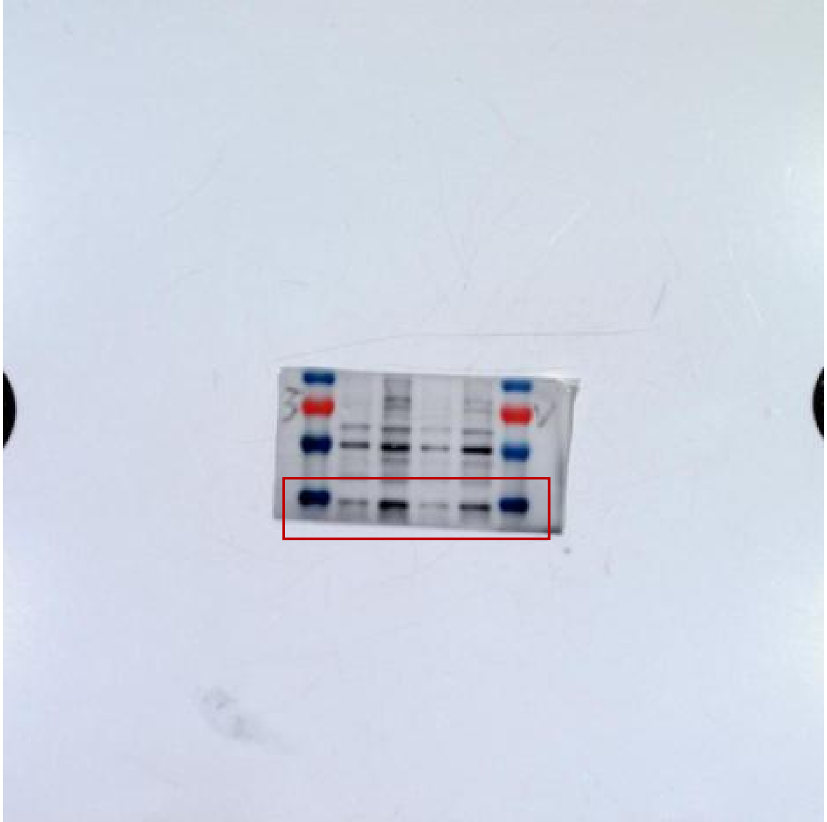

7E　A498　GAPDH

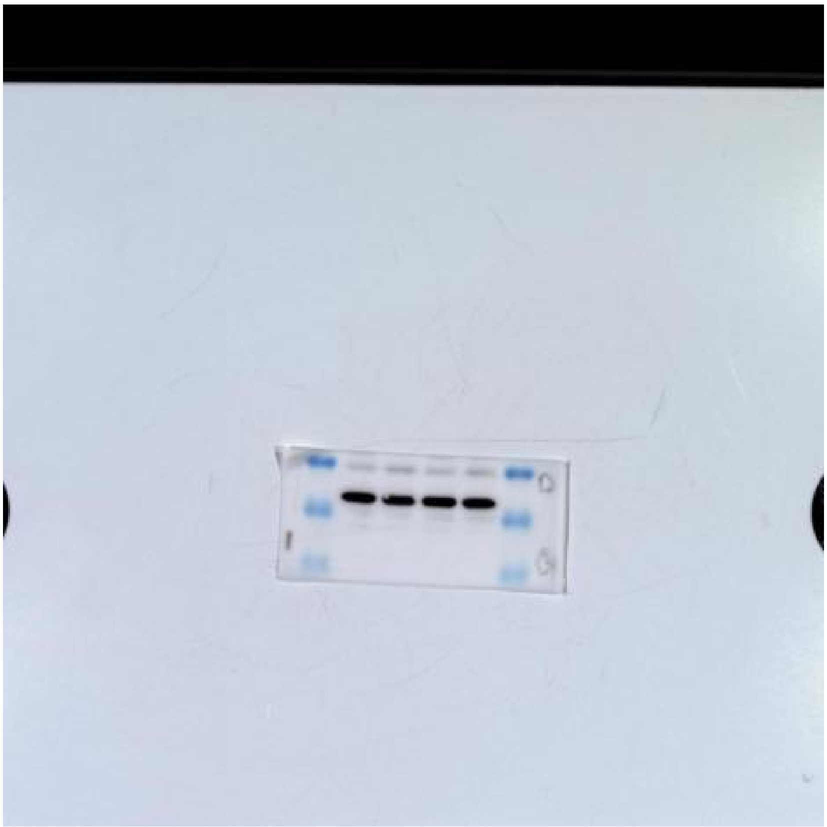

7E　786-O　ZNF24

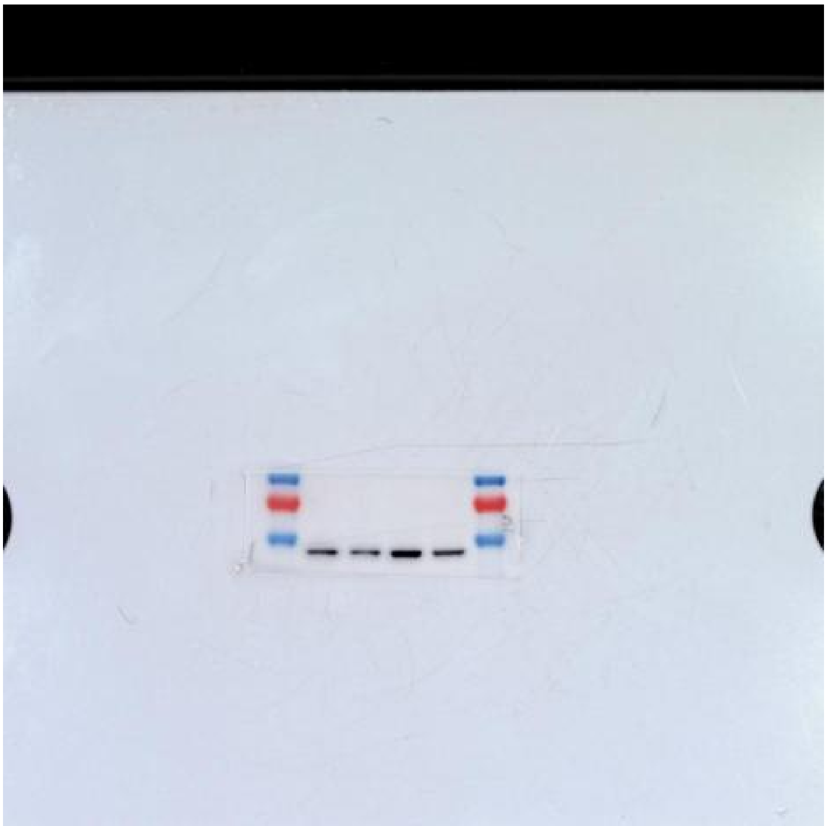

7E 786-O YOD1

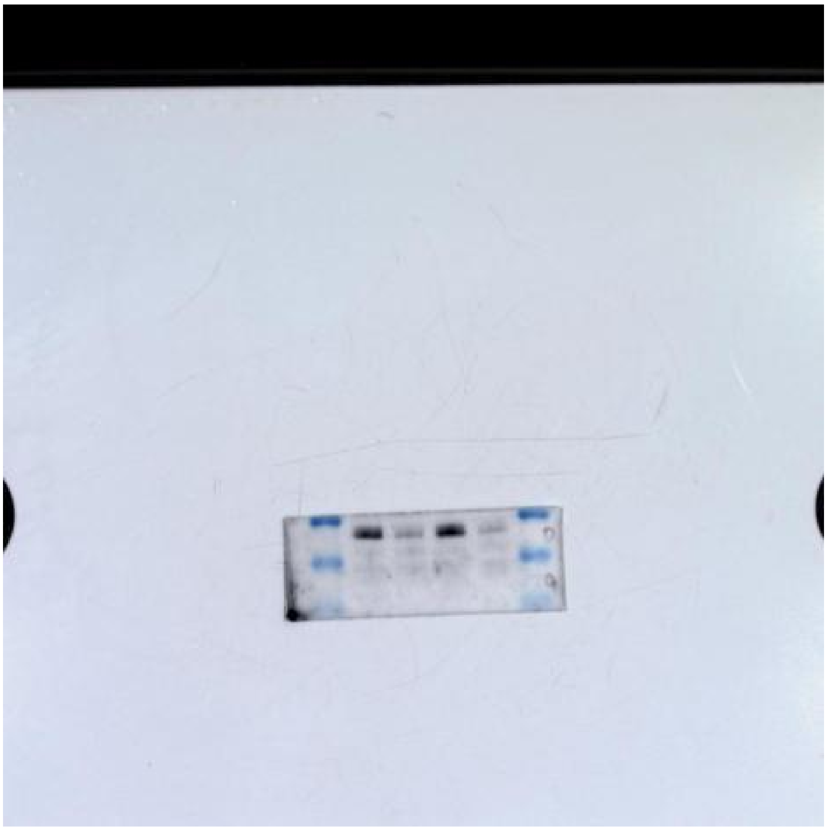

7E 786-O VEGFA

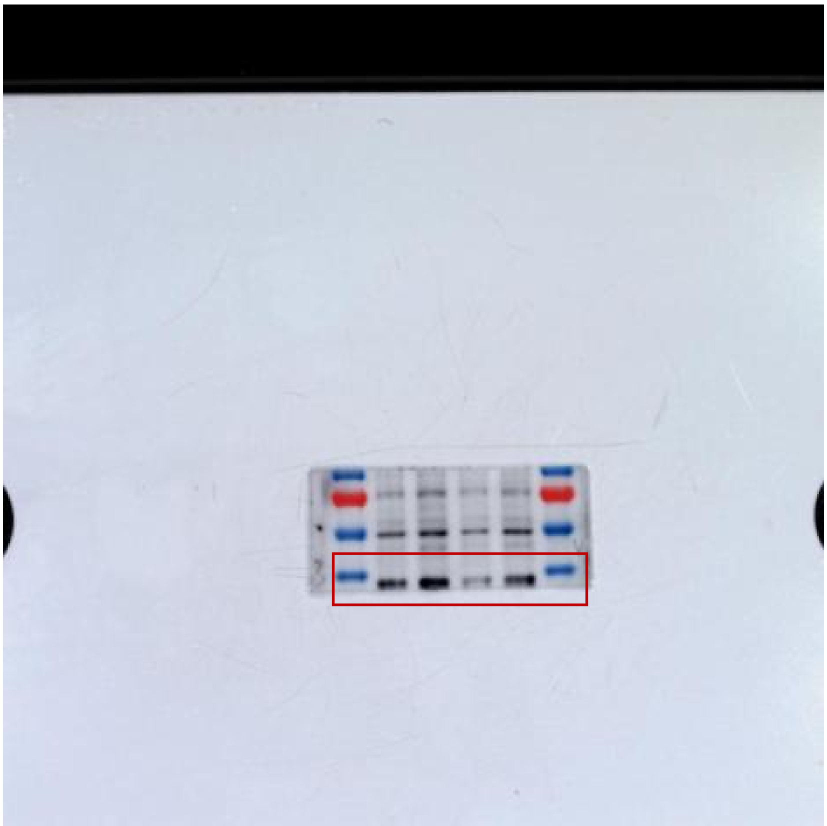

7E 786-O GAPDH

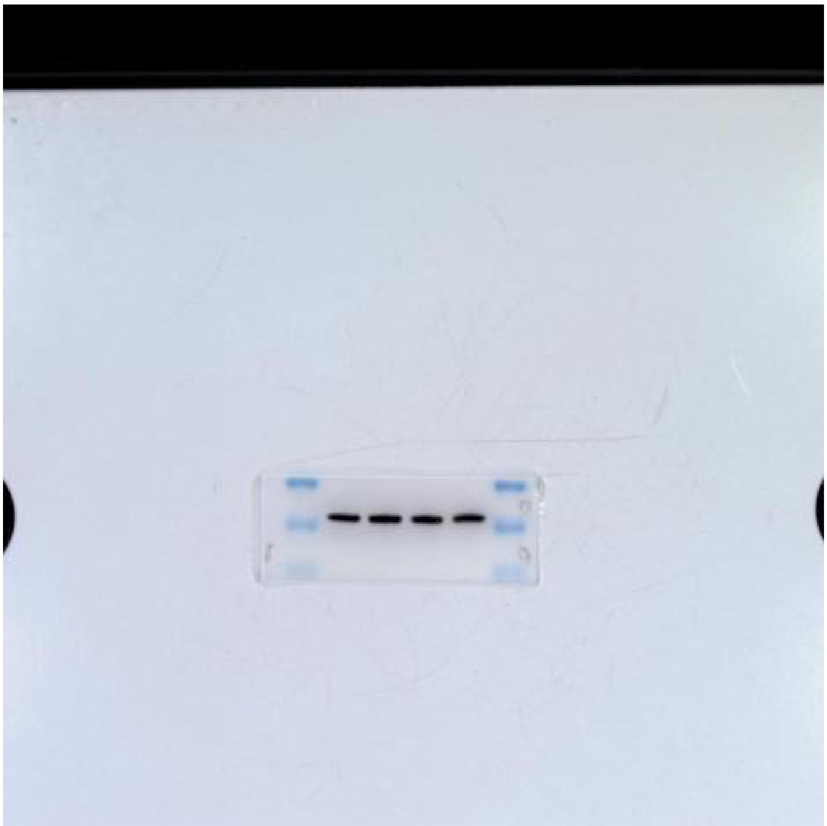

Figure 8

8A ZNF24

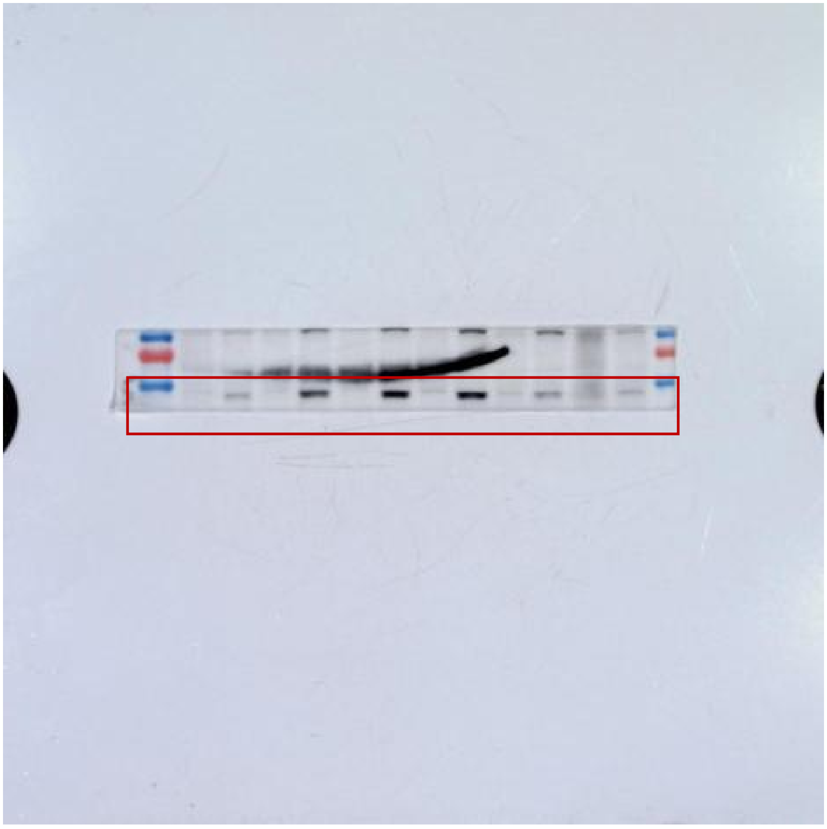

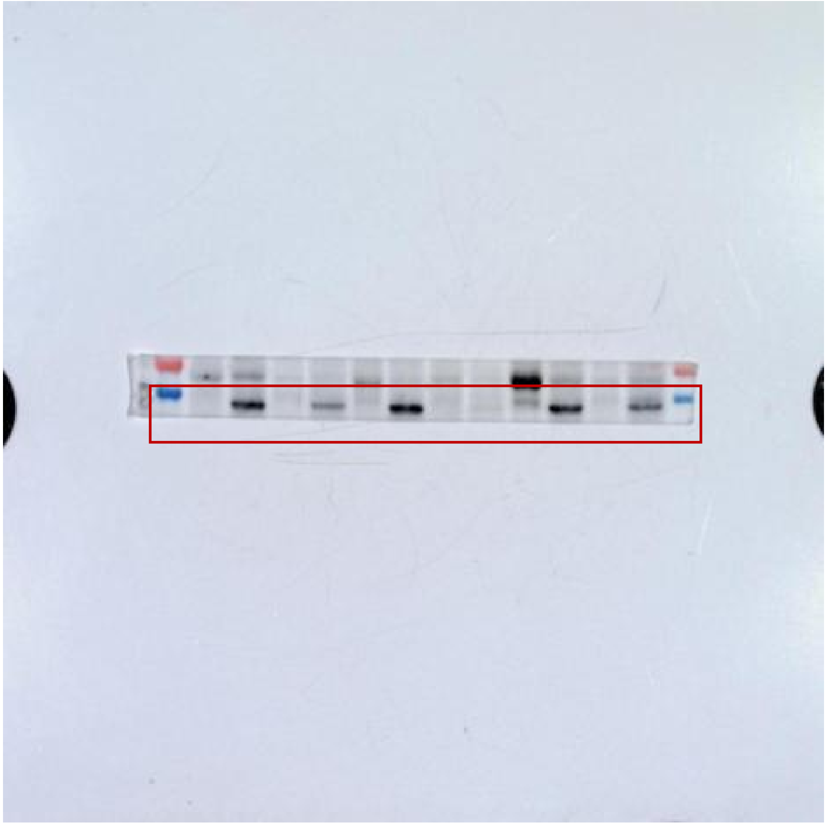

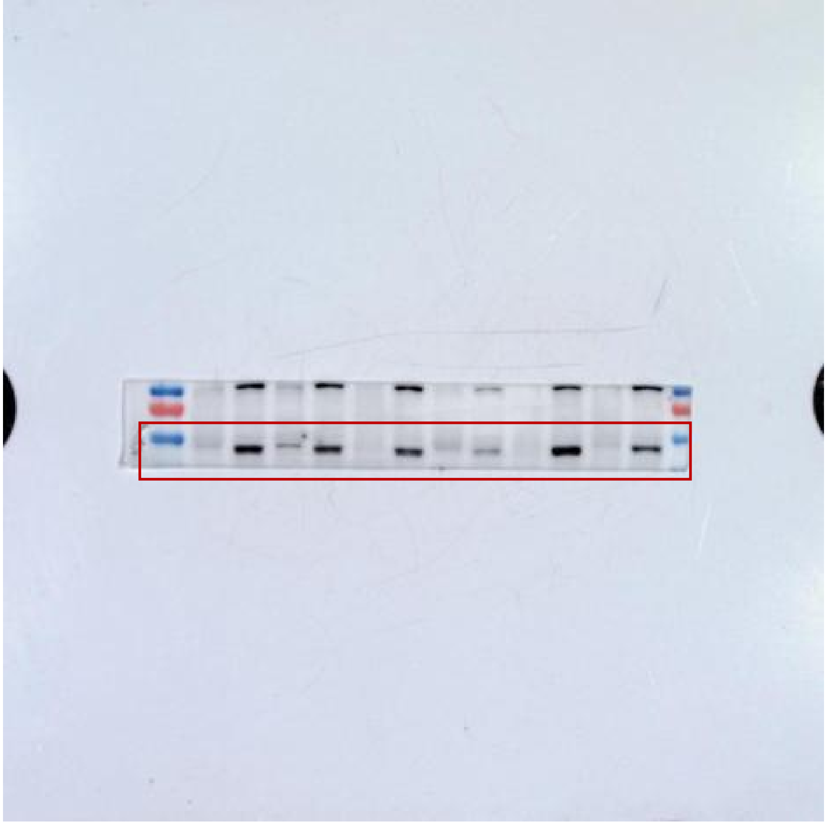

8A YOD1

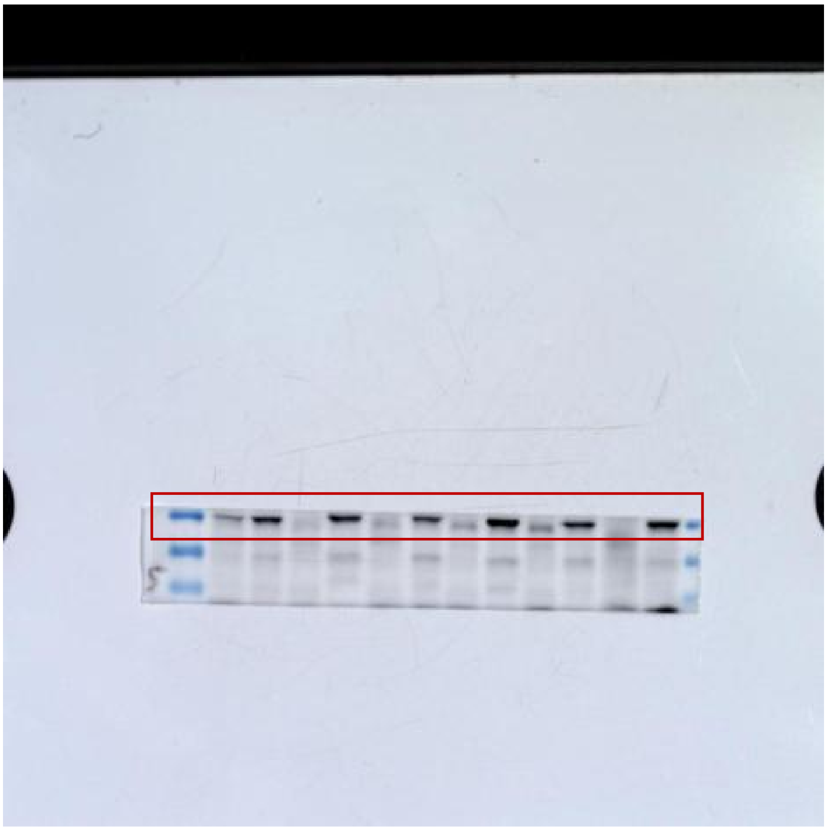

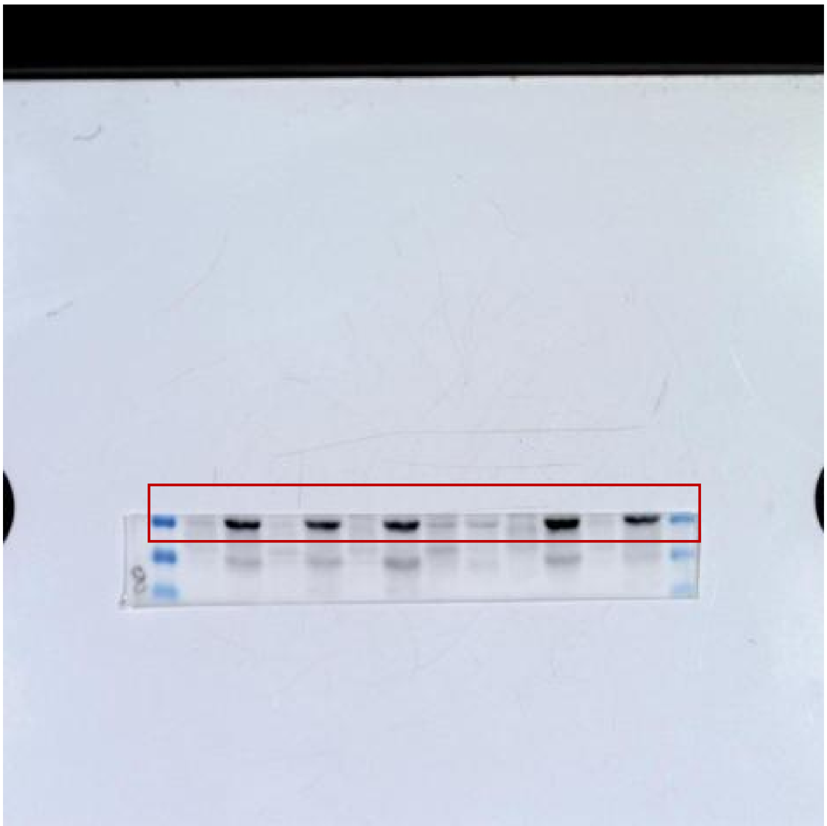

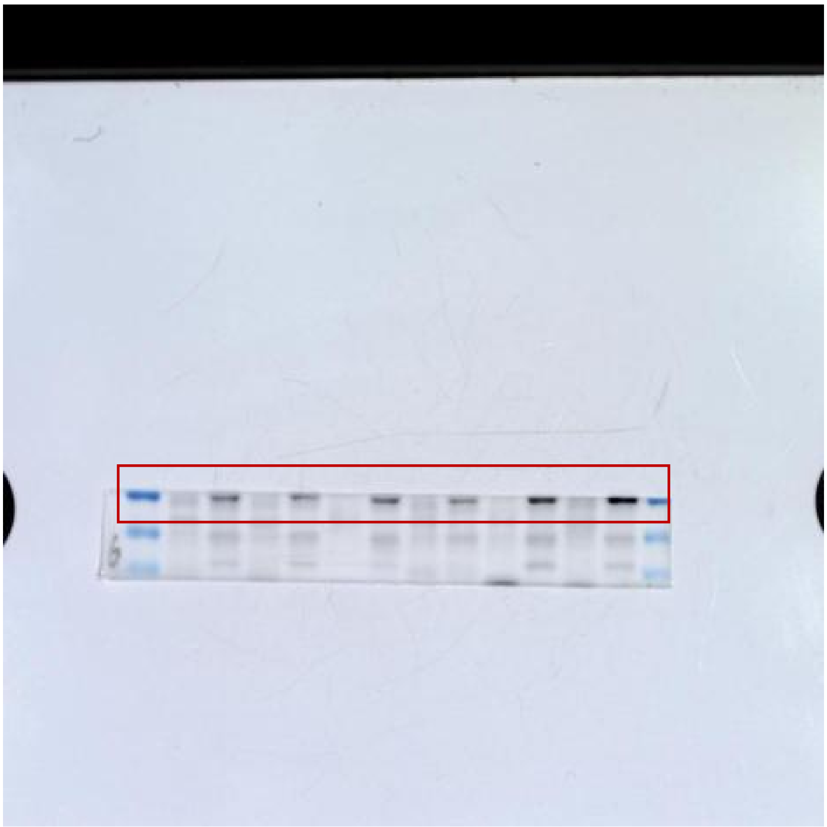

8A GAPDH

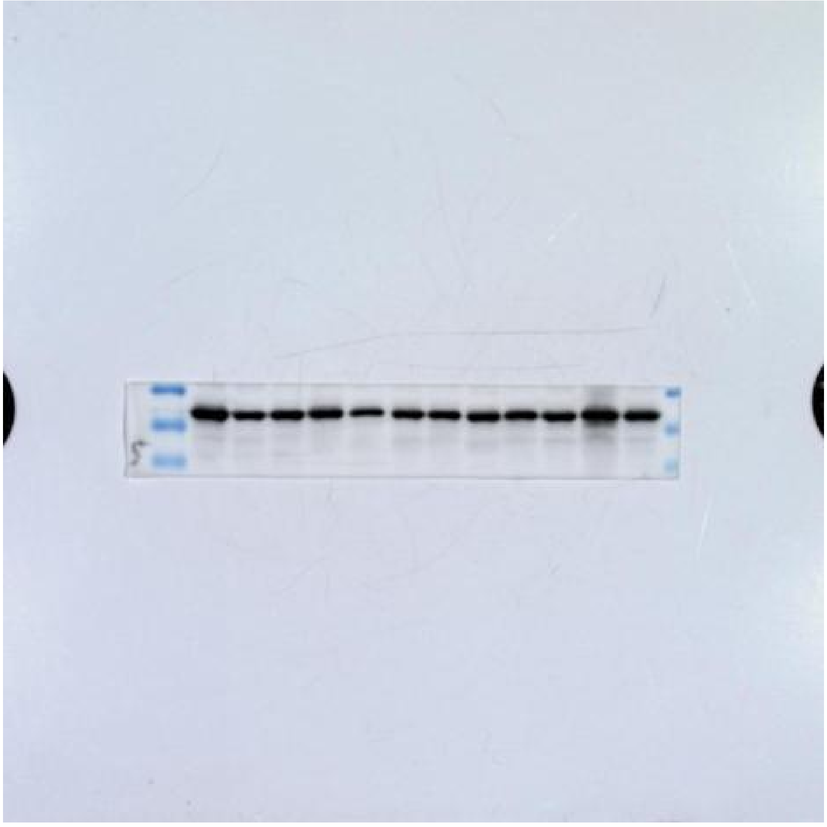

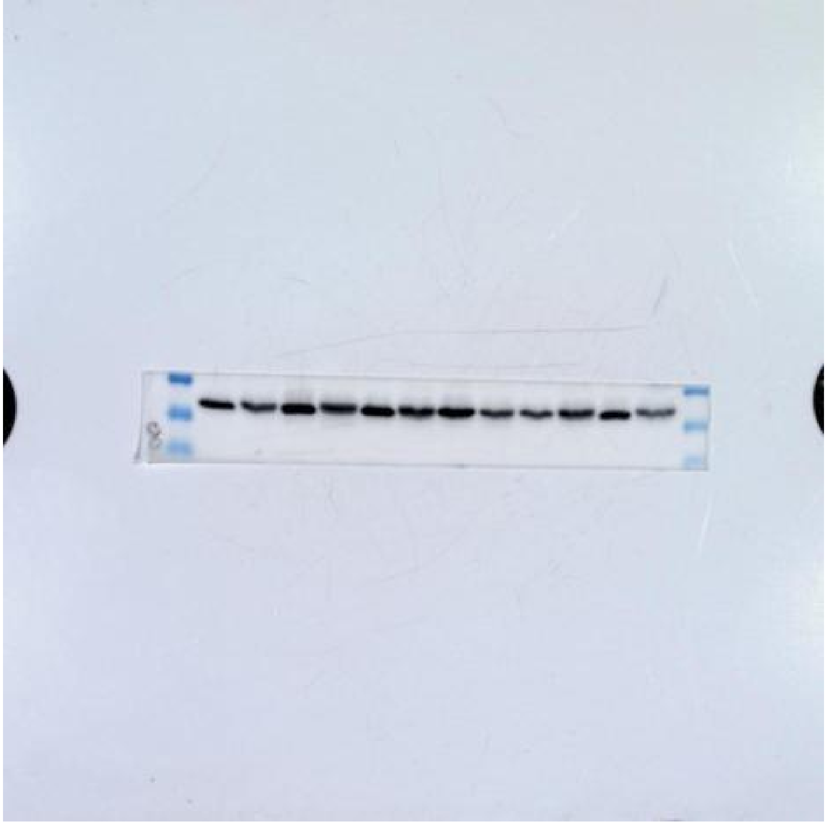

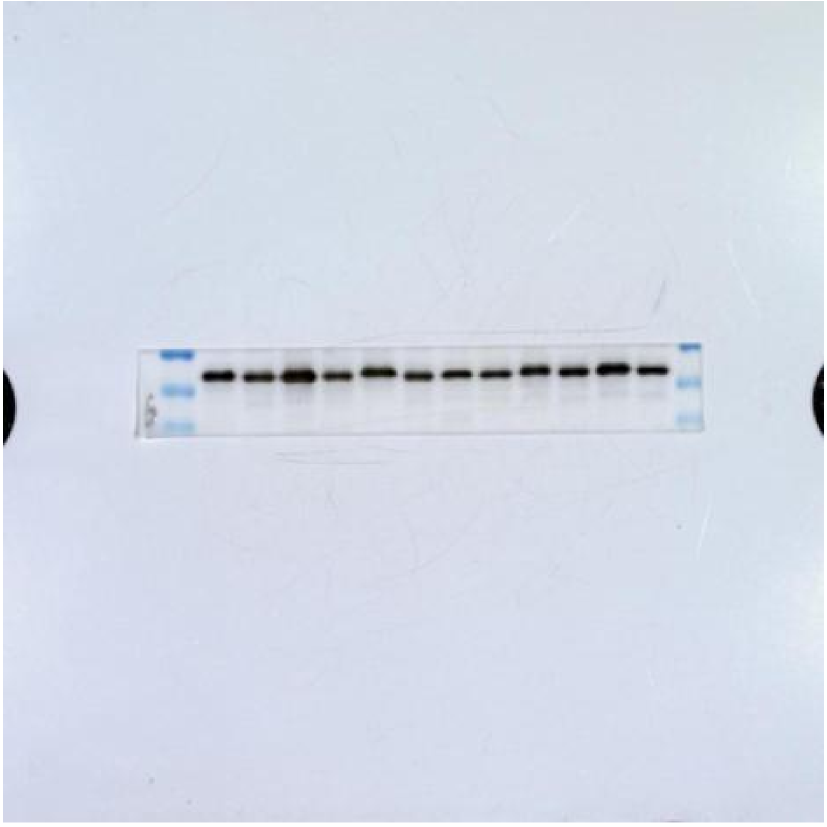

Supplementary Figure 2

S2A YOD1

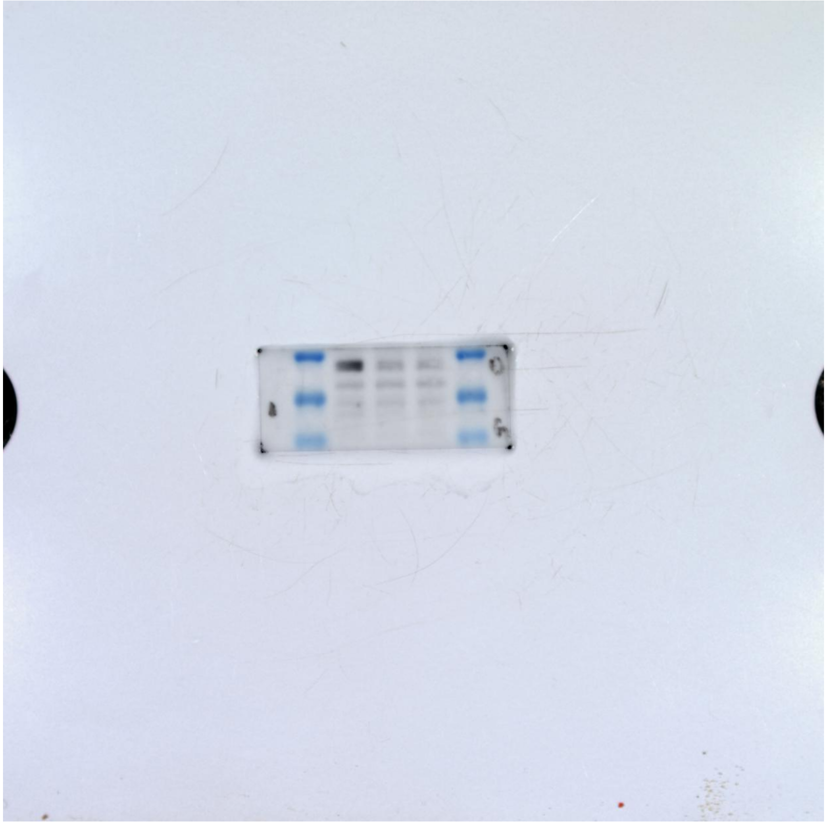

S2A GAPDH

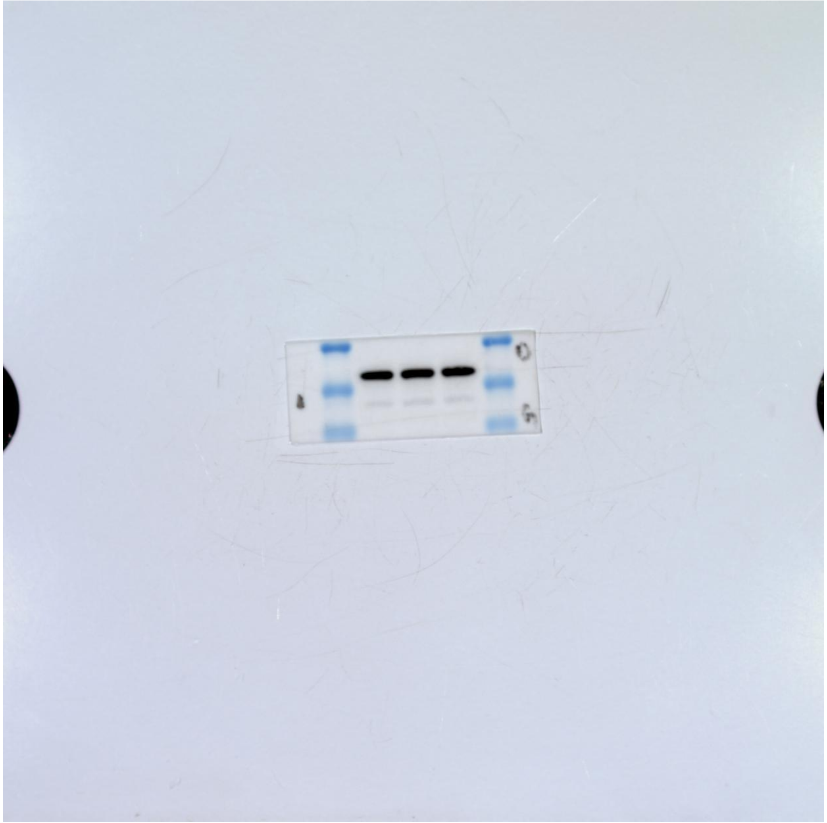

S2B Trim33 A498

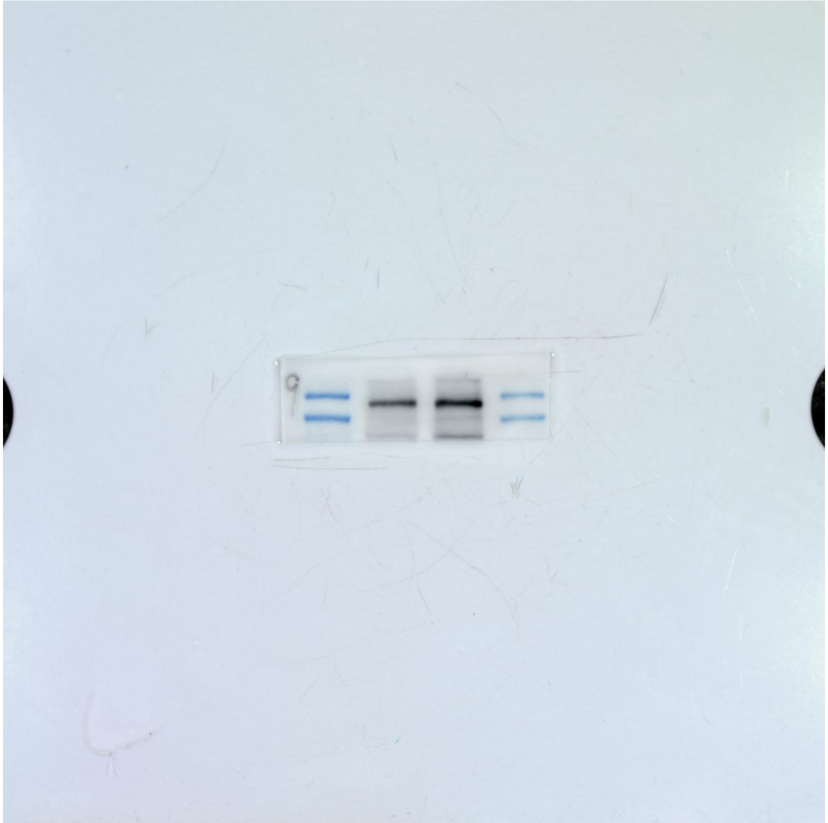

S2B Trim33 786-O

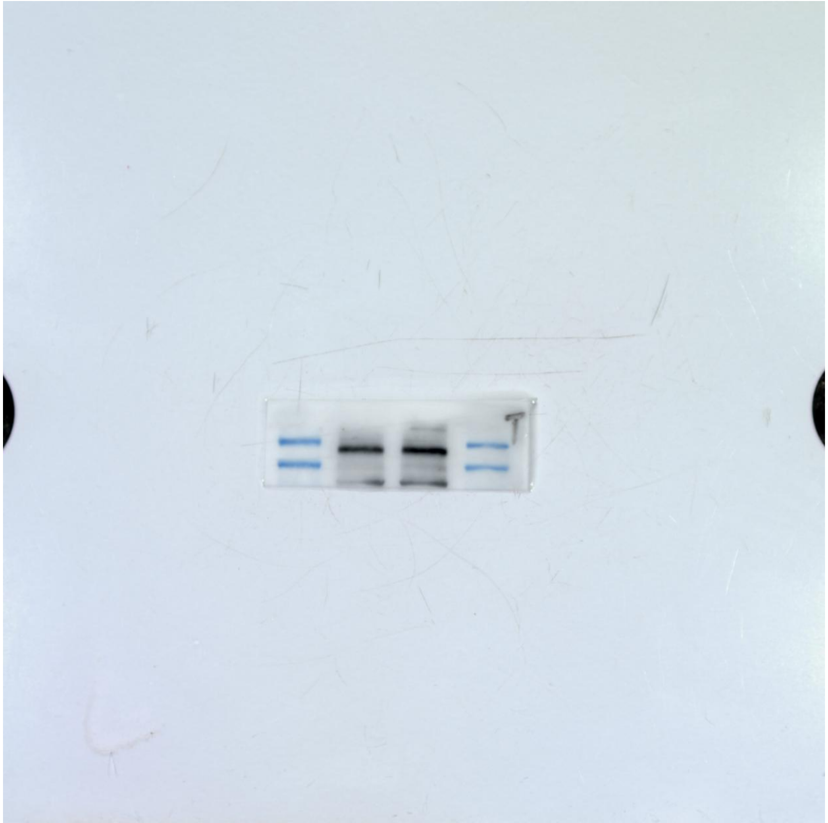

S2B p-β-catenin A498

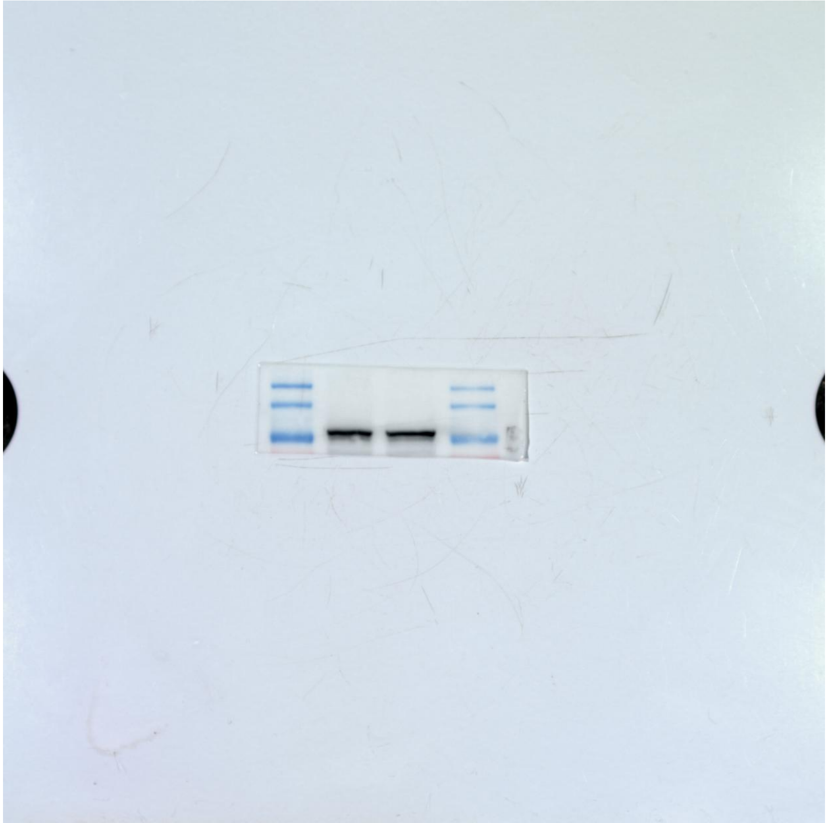

S2B p-β-catenin 786-O

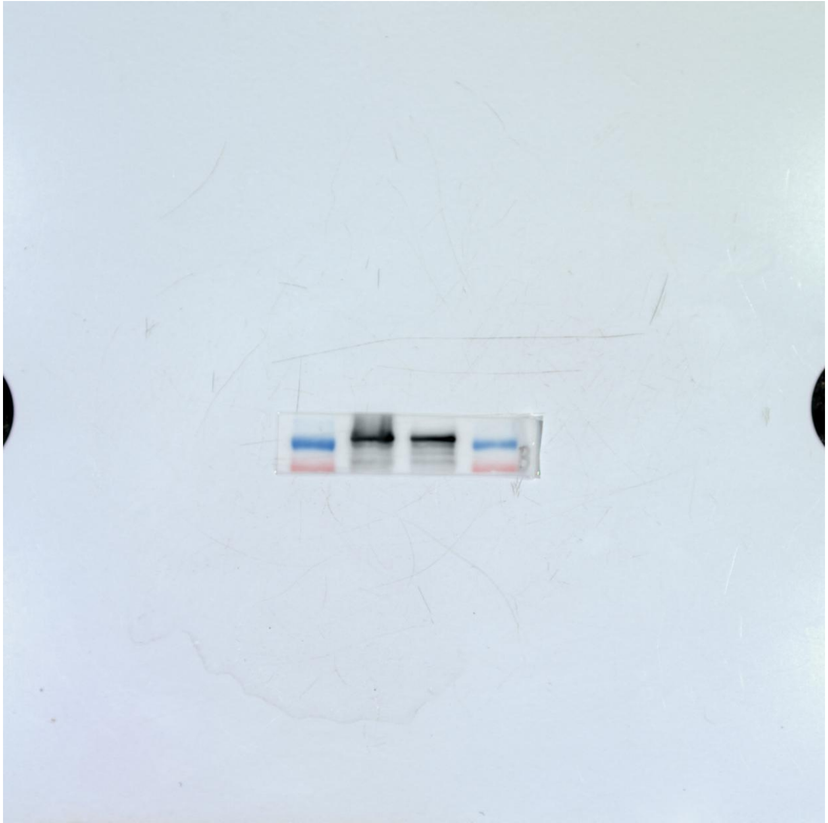

S2B β-catenin A498

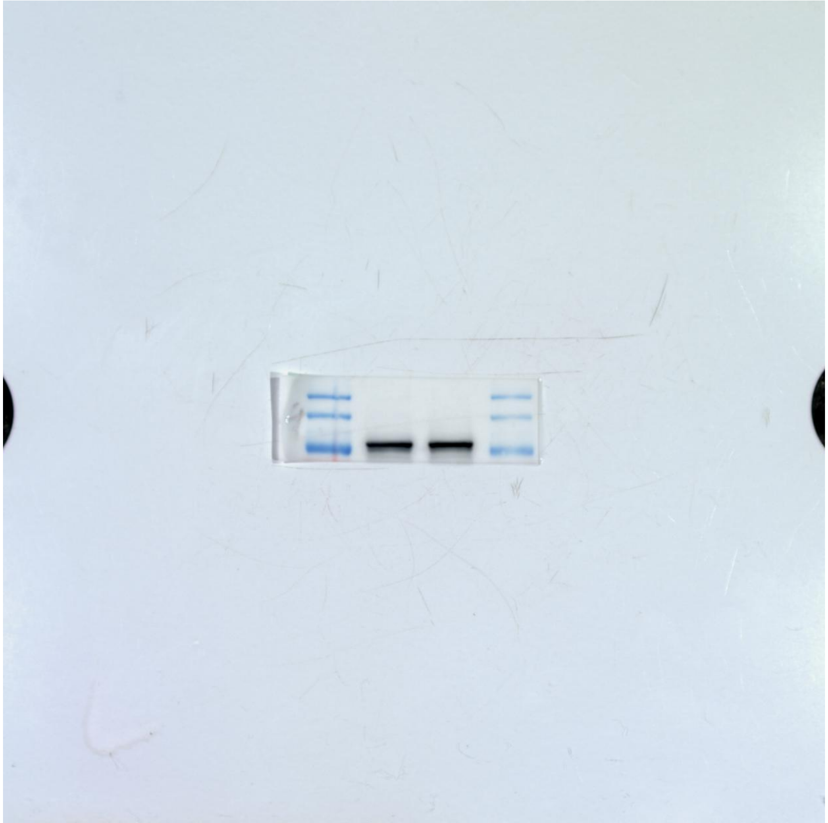

S2B β-catenin 786-O

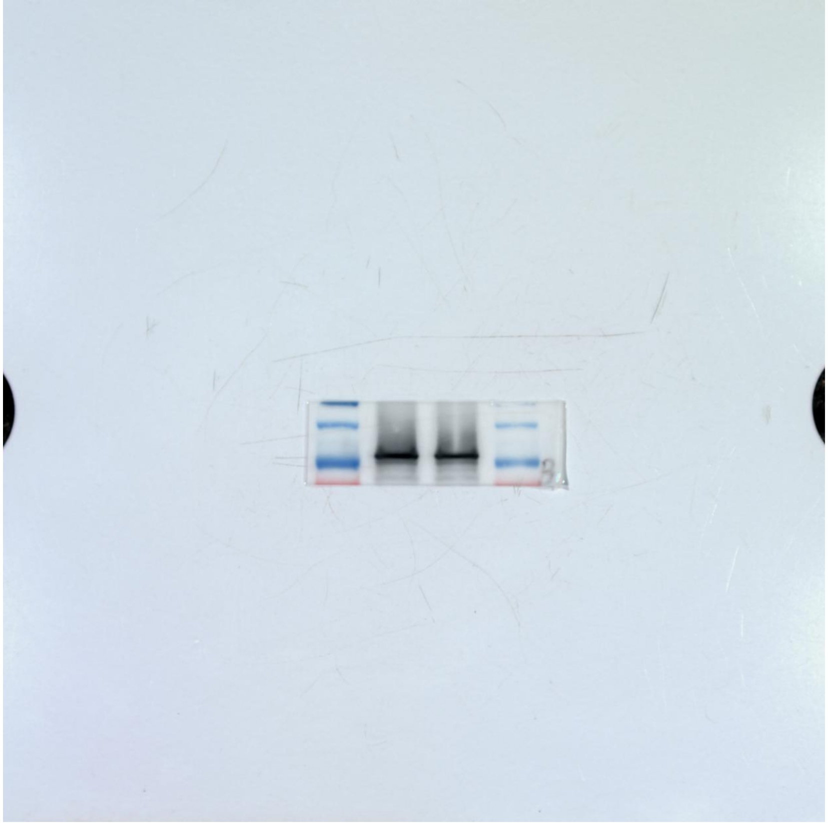

S2B YAP A498

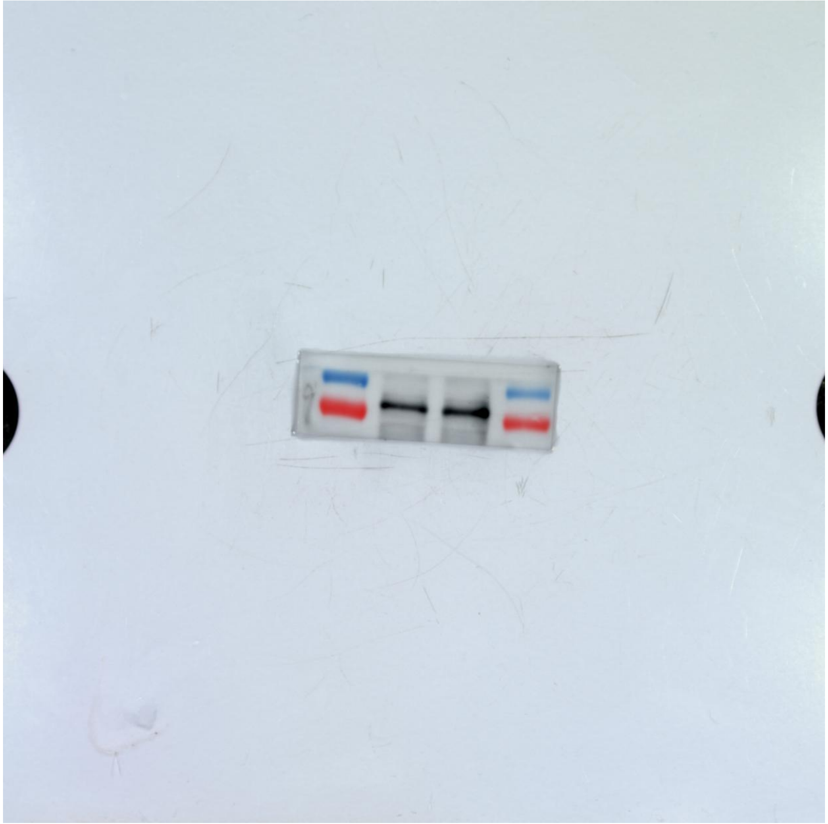

S2B YAP 786-O

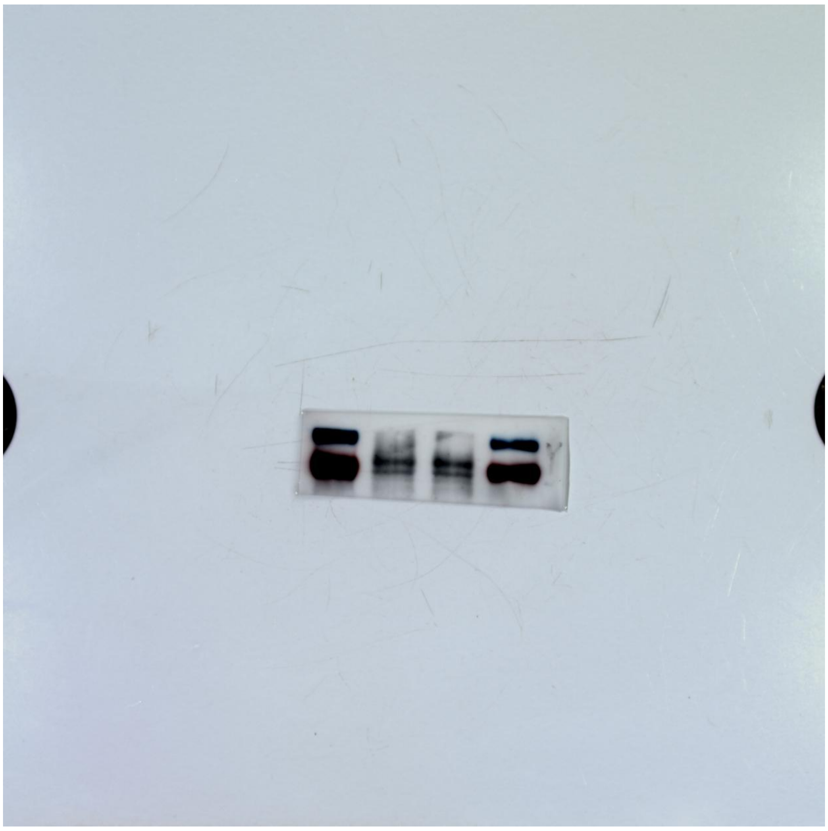

S2B p-P65 A498

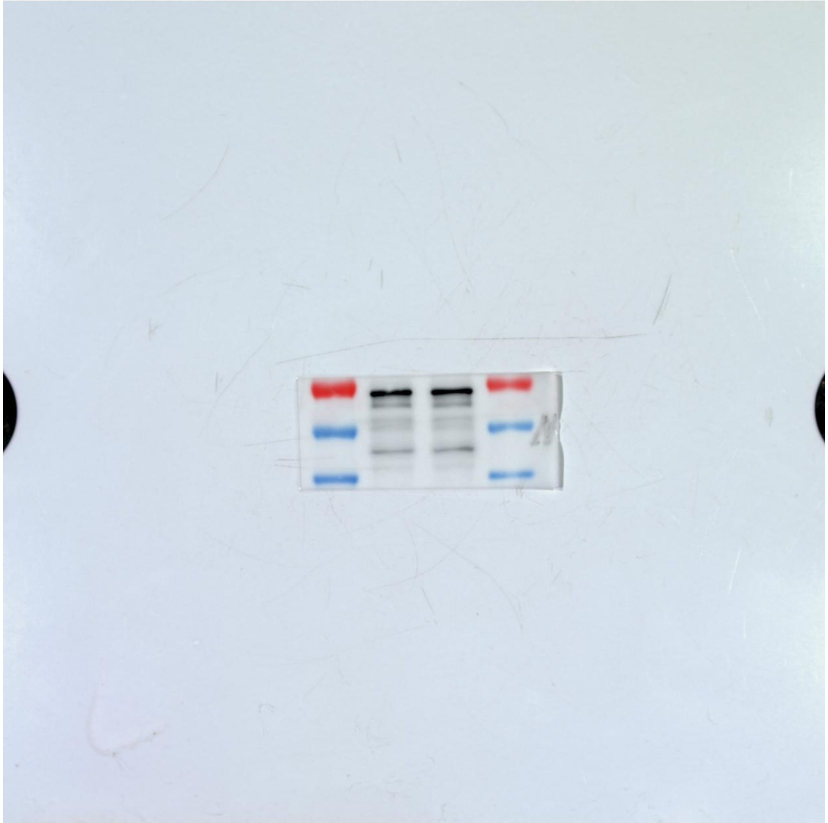

S2B p-P65 786-O

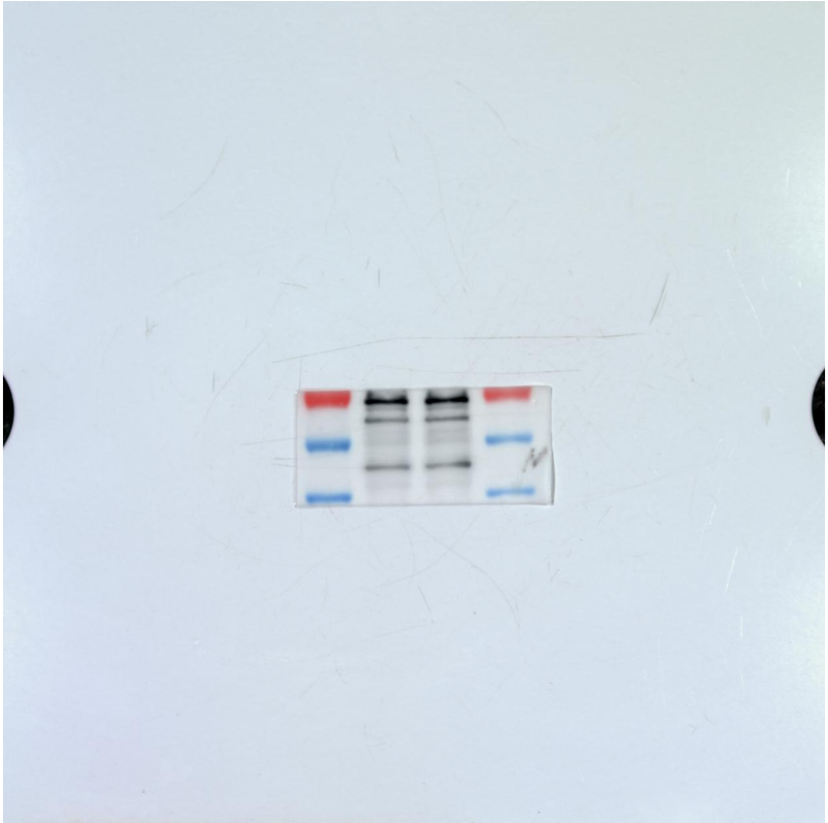

S2B P65 A498

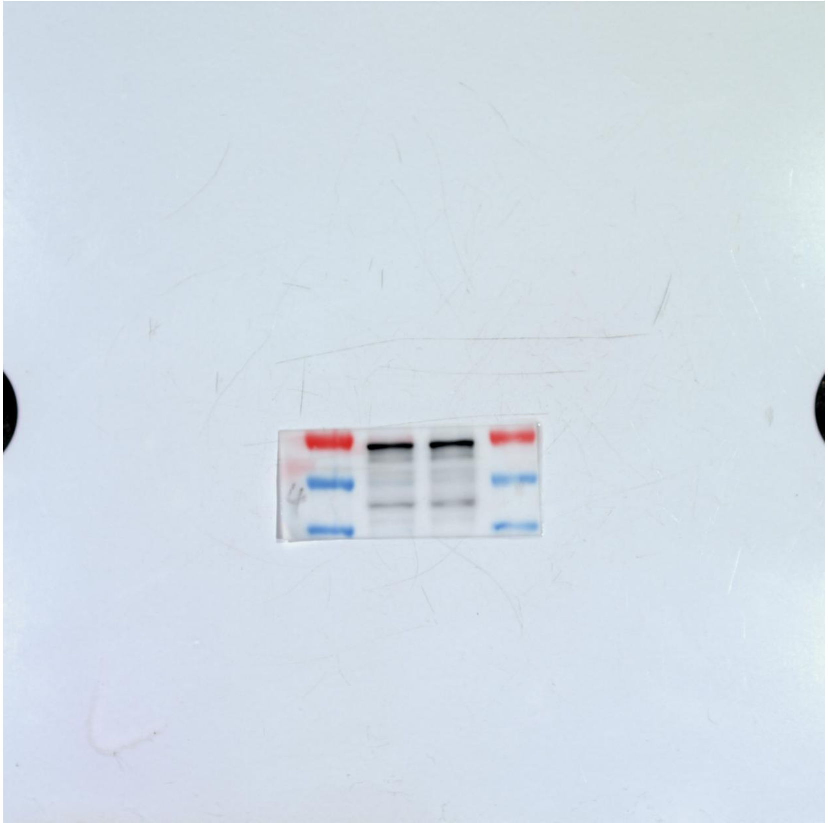

S2B P65 786-O

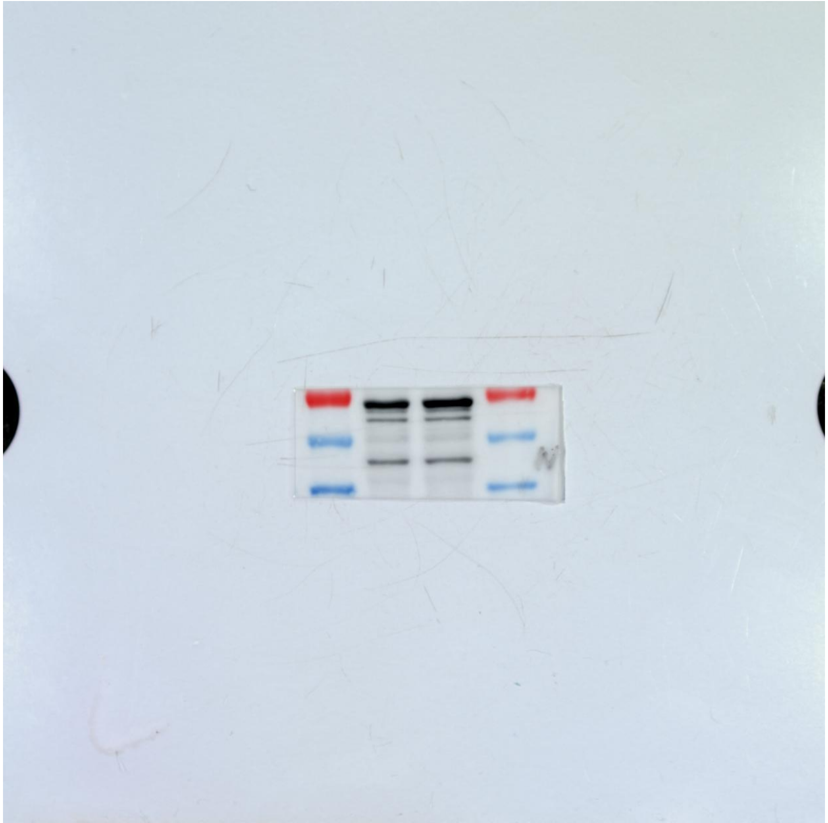

S2B P53 A498

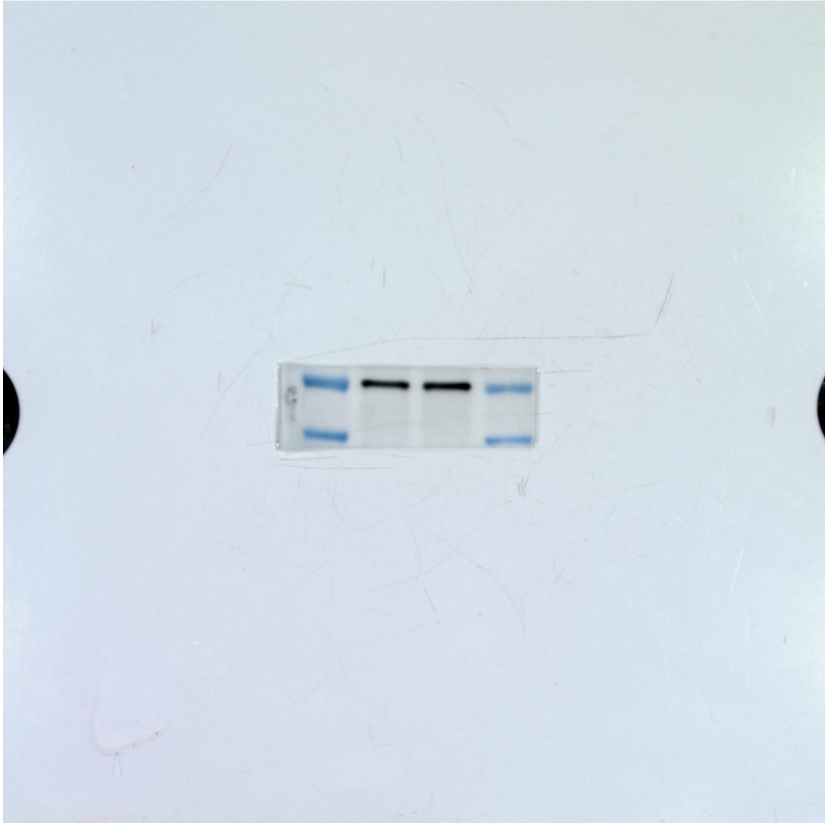

S2B P53 786-O

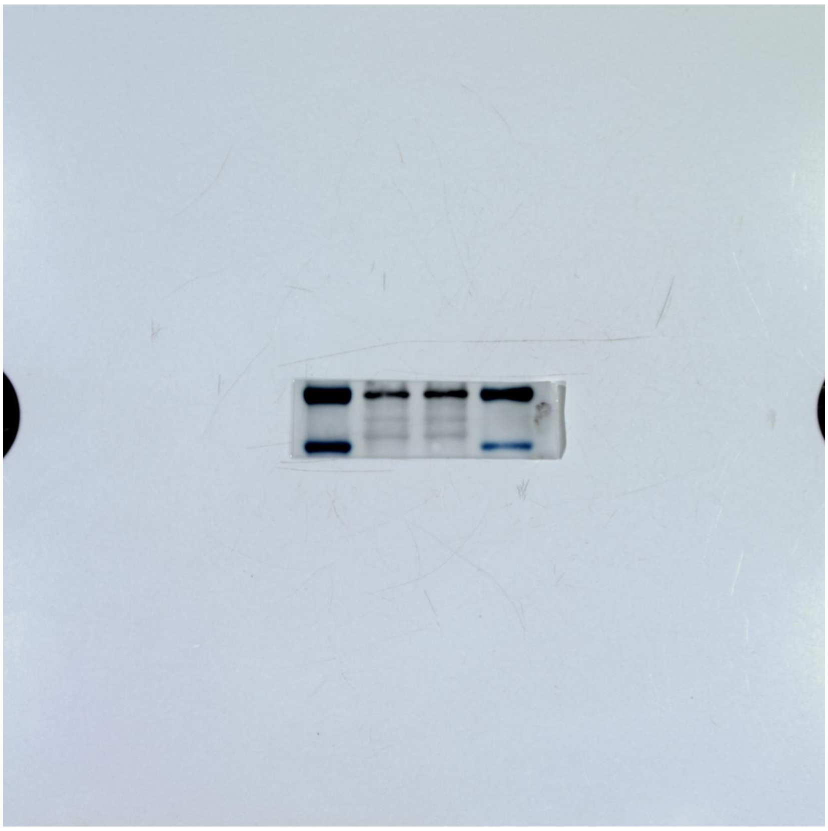

S2B Flag A498

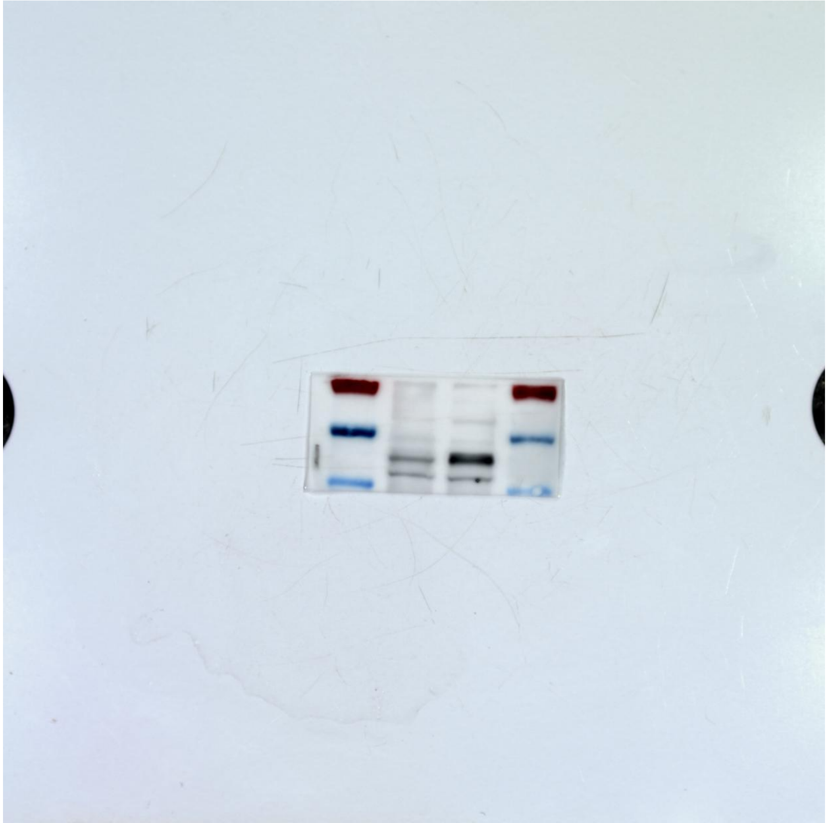

S2B Flag 786-O

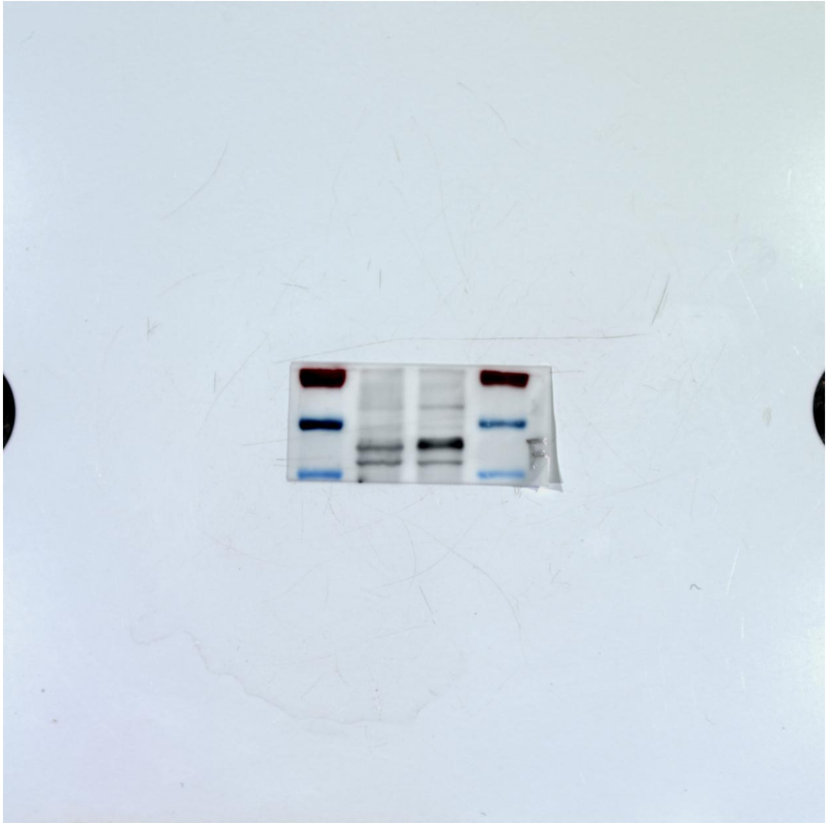

S2B TGF-β3 A498

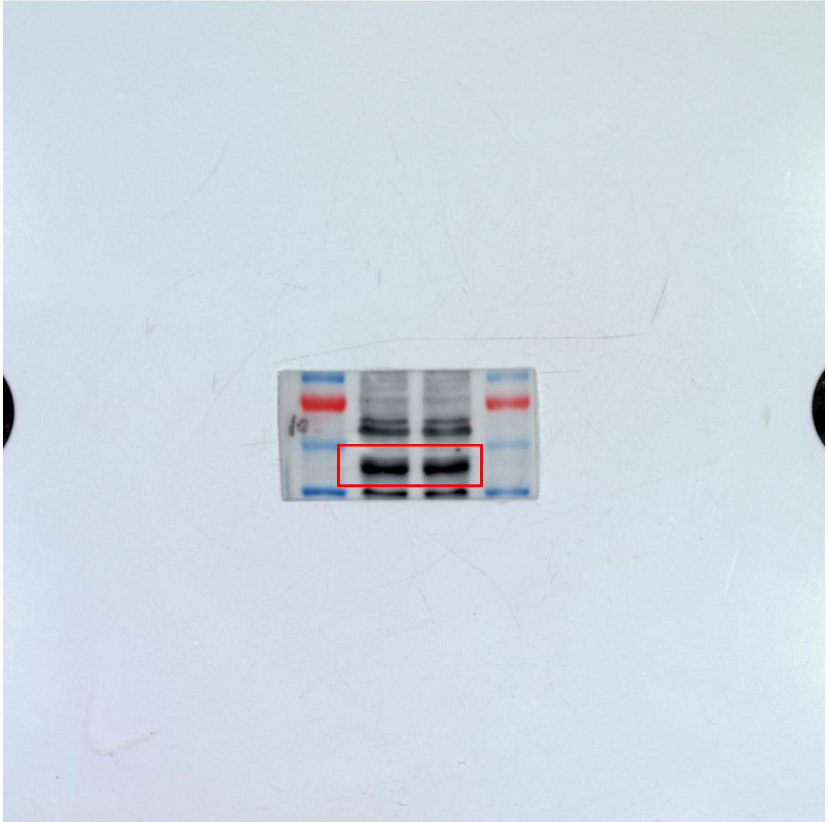

S2B TGF-β3 786-O

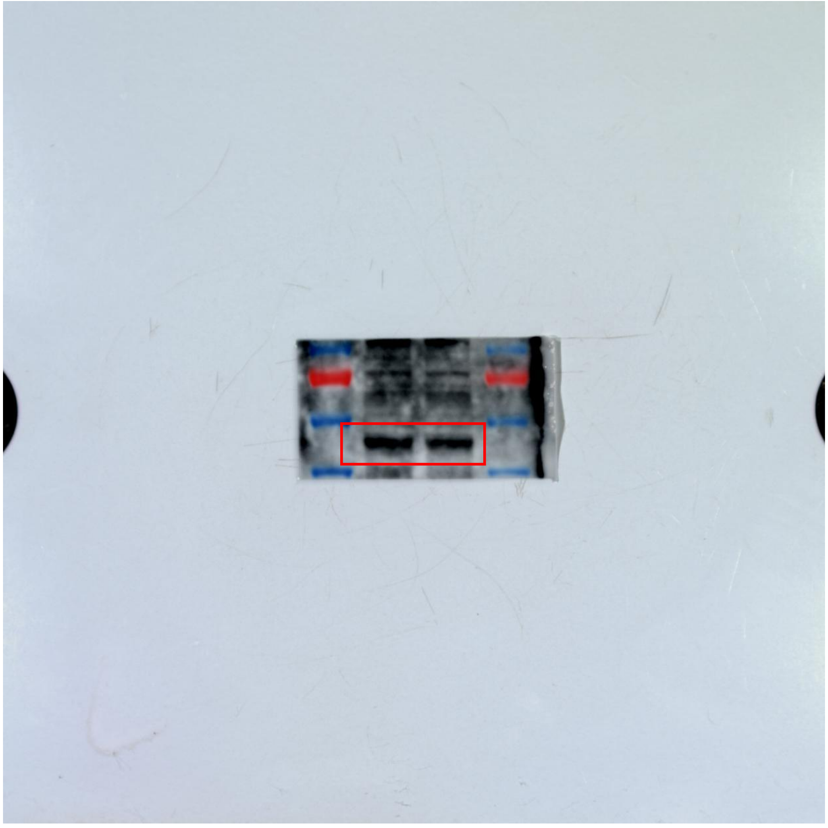

S2B CDK1 A498 left

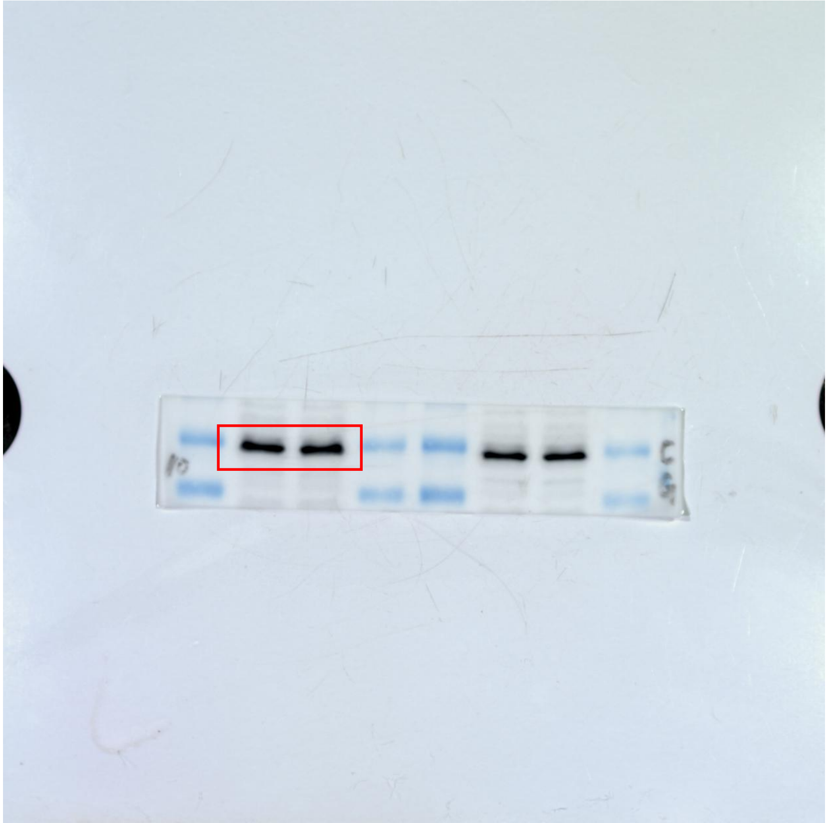

S2B CDK1 786-O right

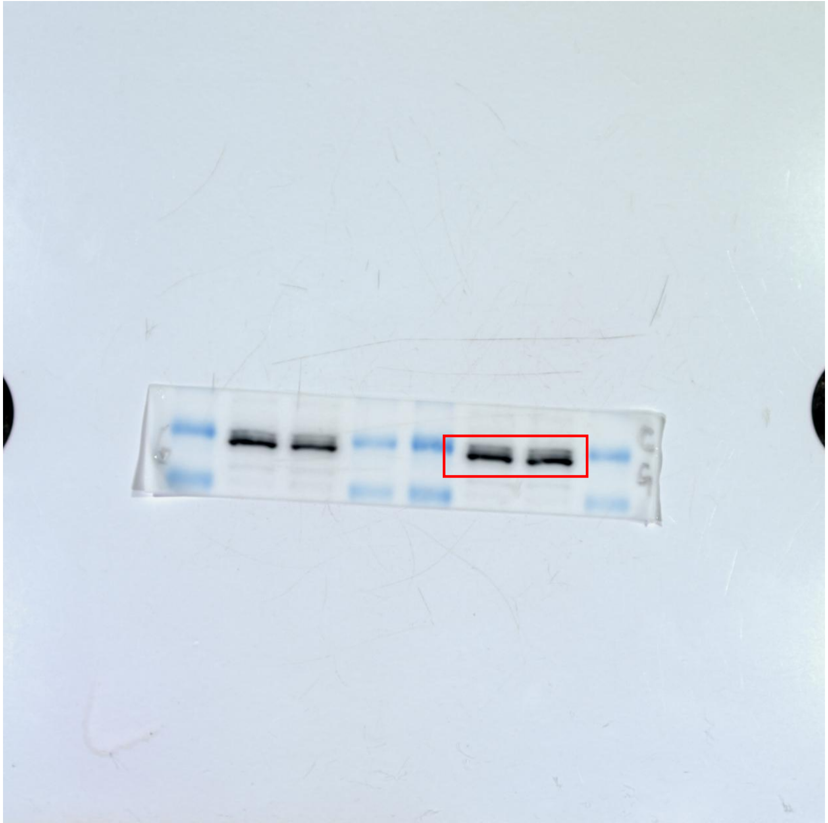

S2B GAPDH A498

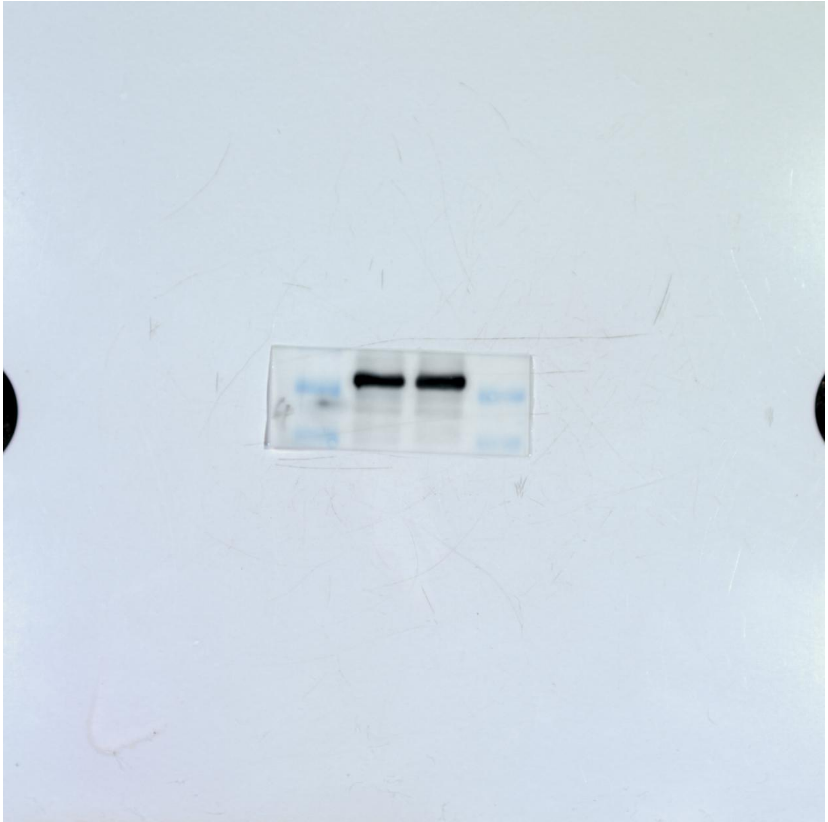

S2B GAPDH 786-O

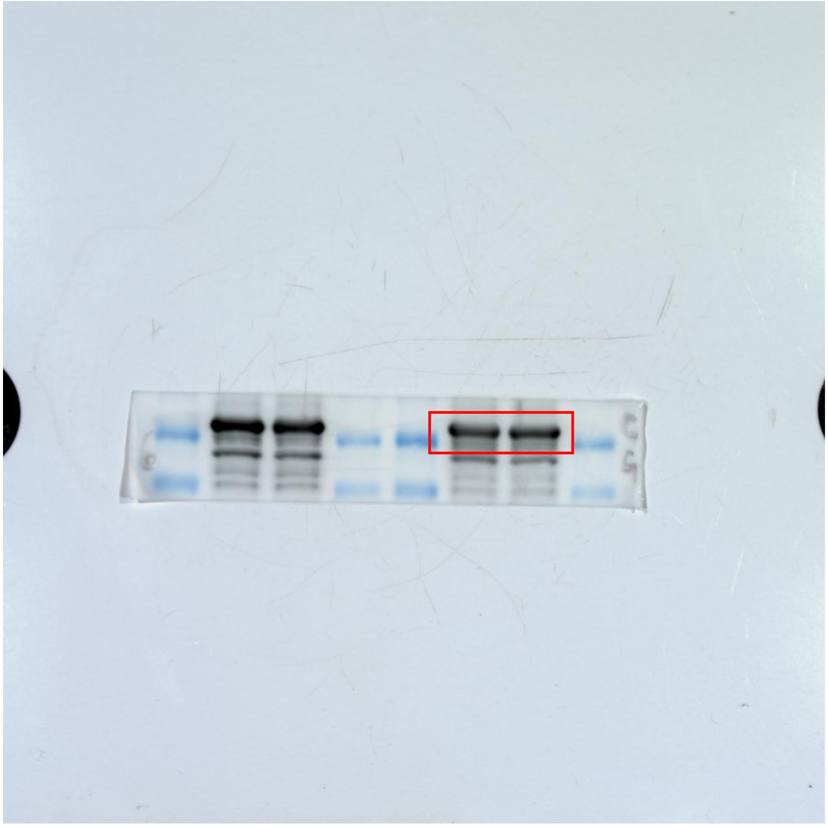

Supplementary Figure 4

S4A ZNF24 A498

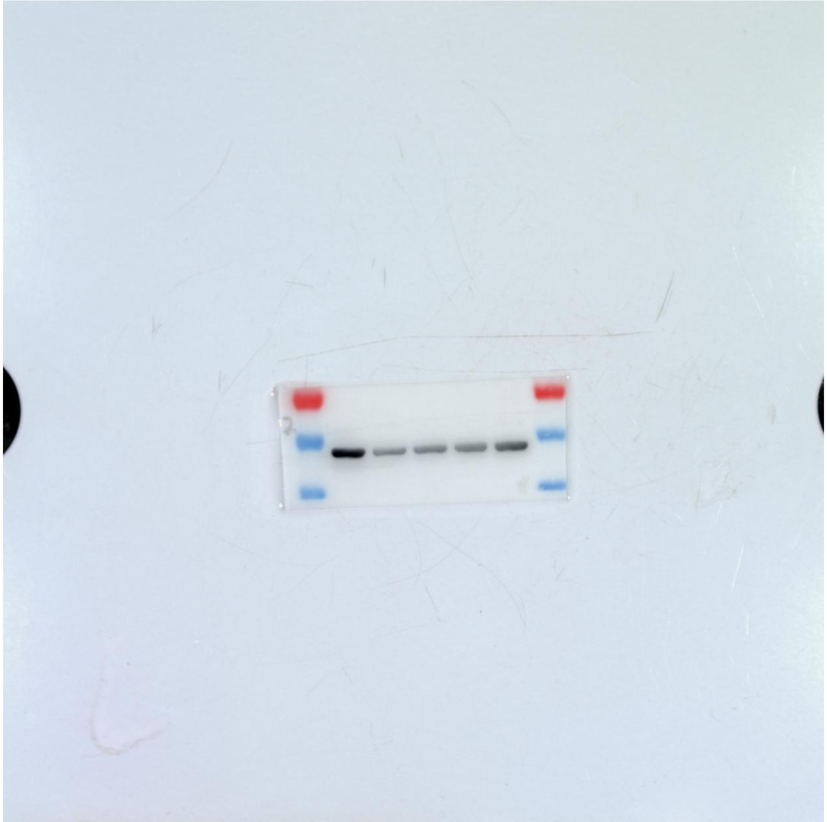

S4A ZNF24 786-O

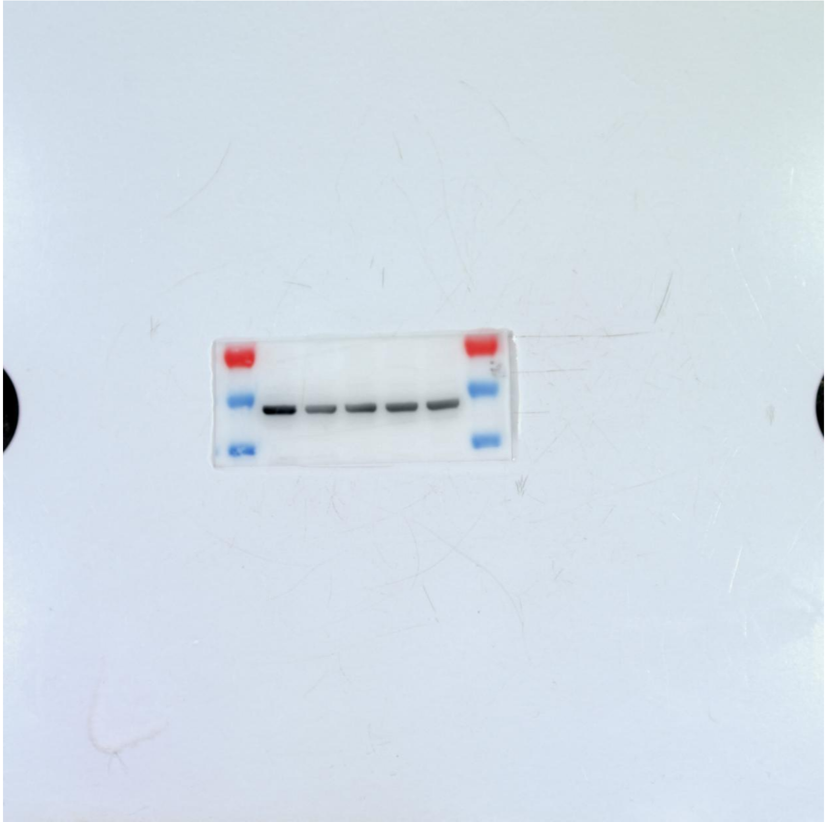

S4A GAPDH A498

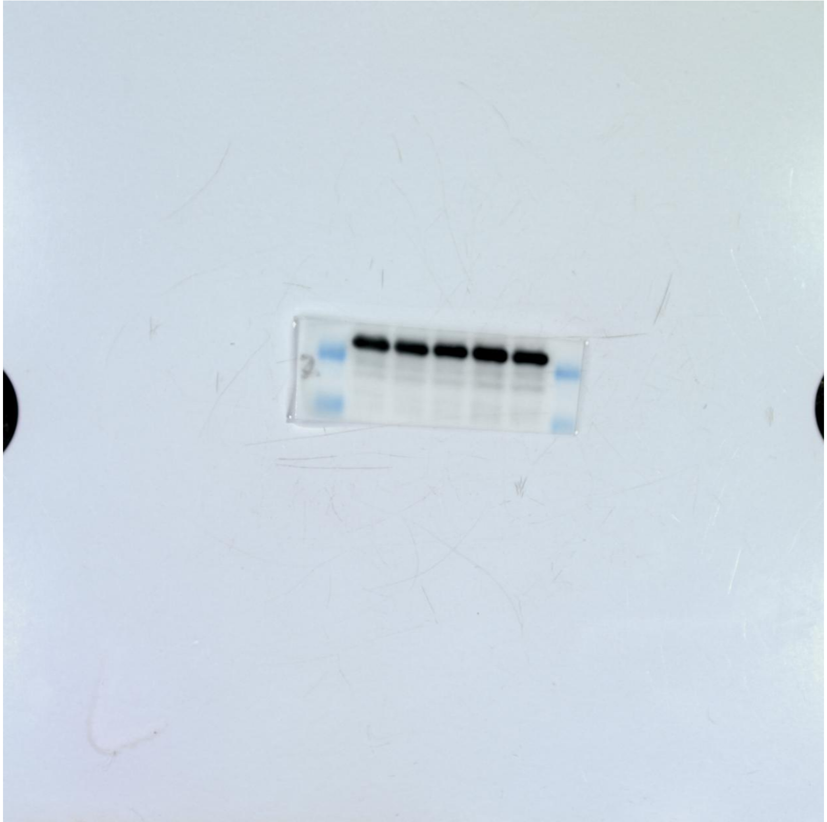

S4A GAPDH 786-O

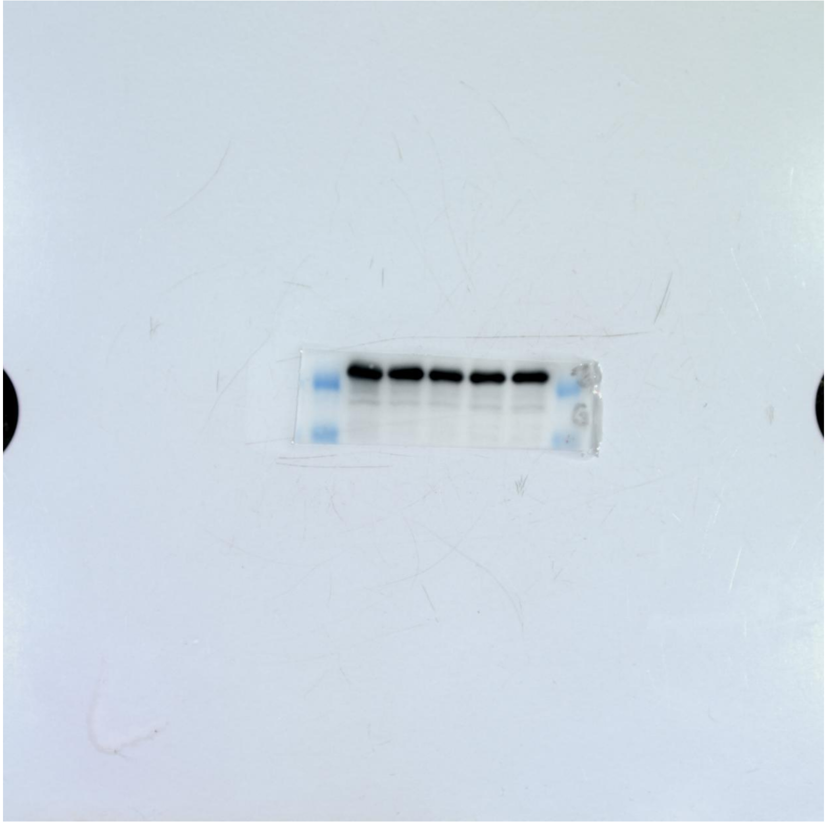

Supplementary Figure 5

S5A VEGFA A498

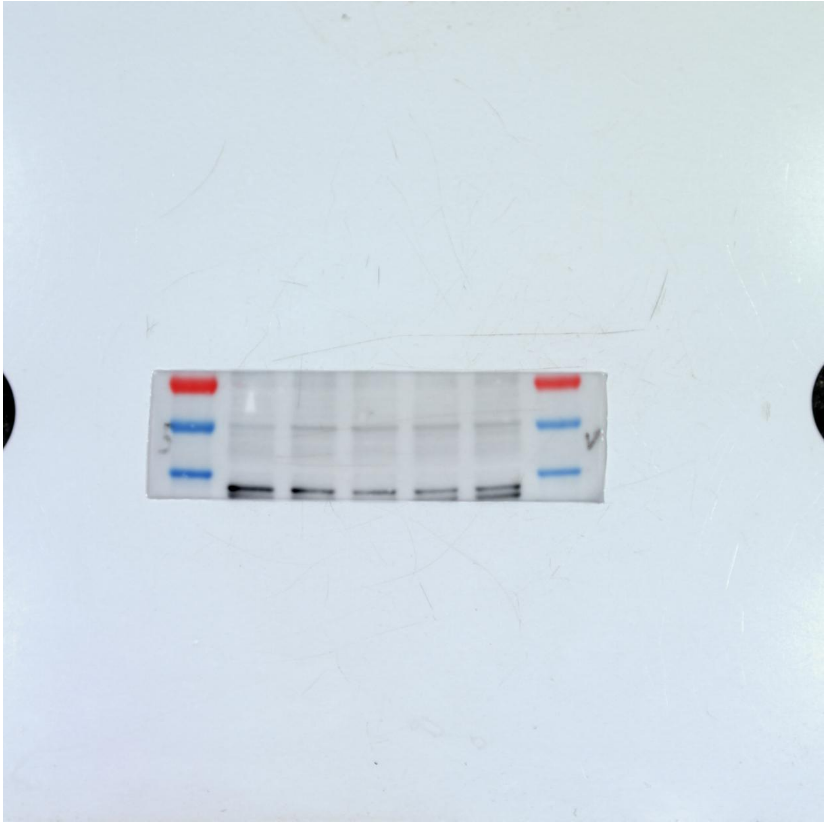

S5A VEGFA 786-O

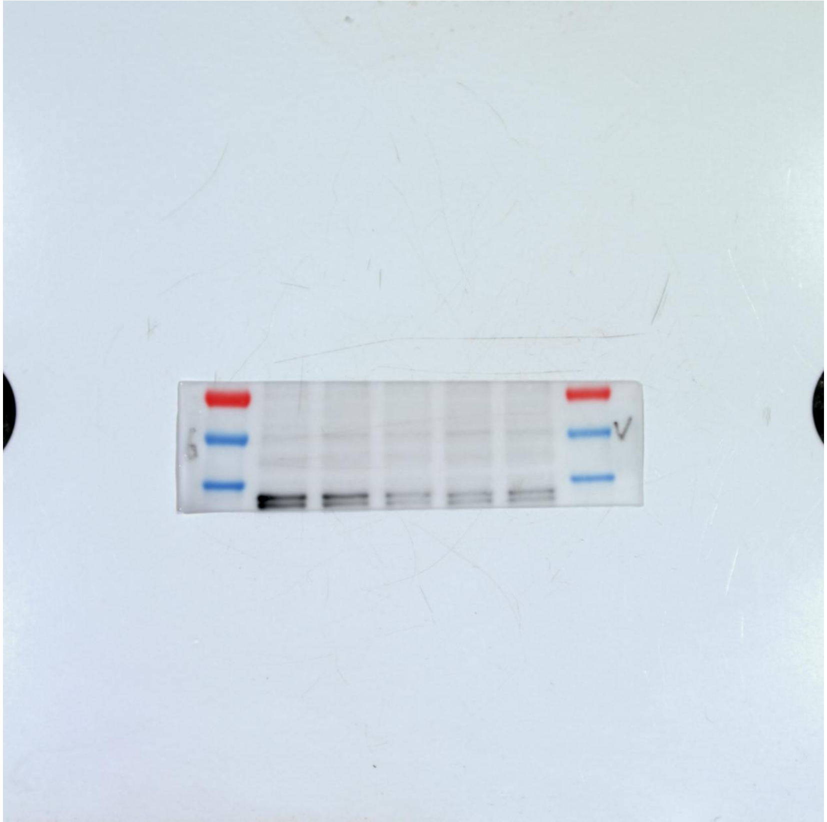

S5A GAPDH A498

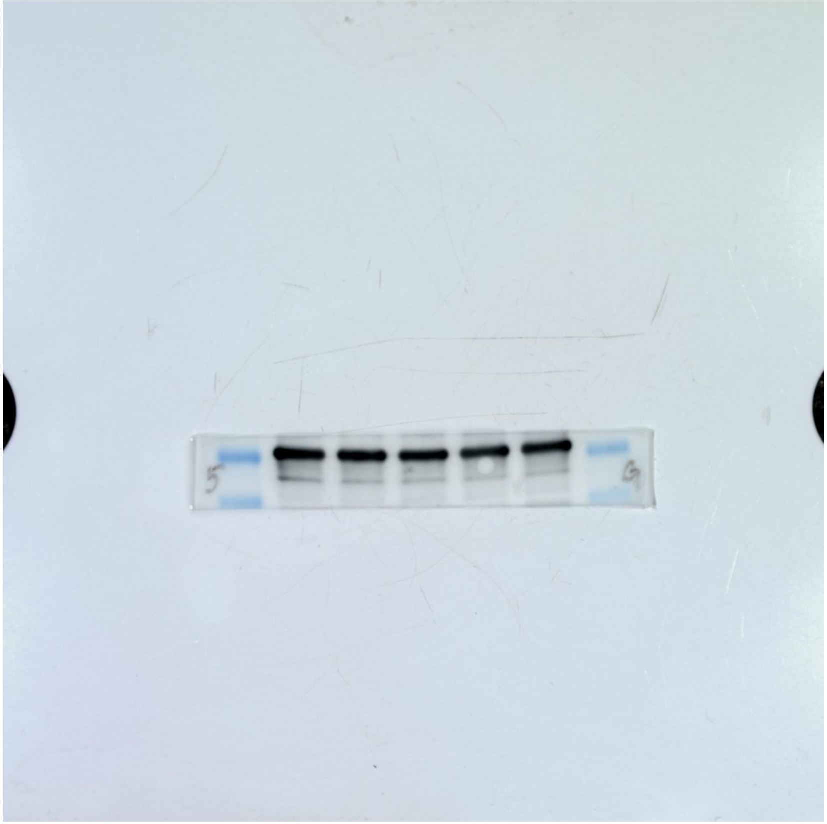

S5A GAPDH 786-O

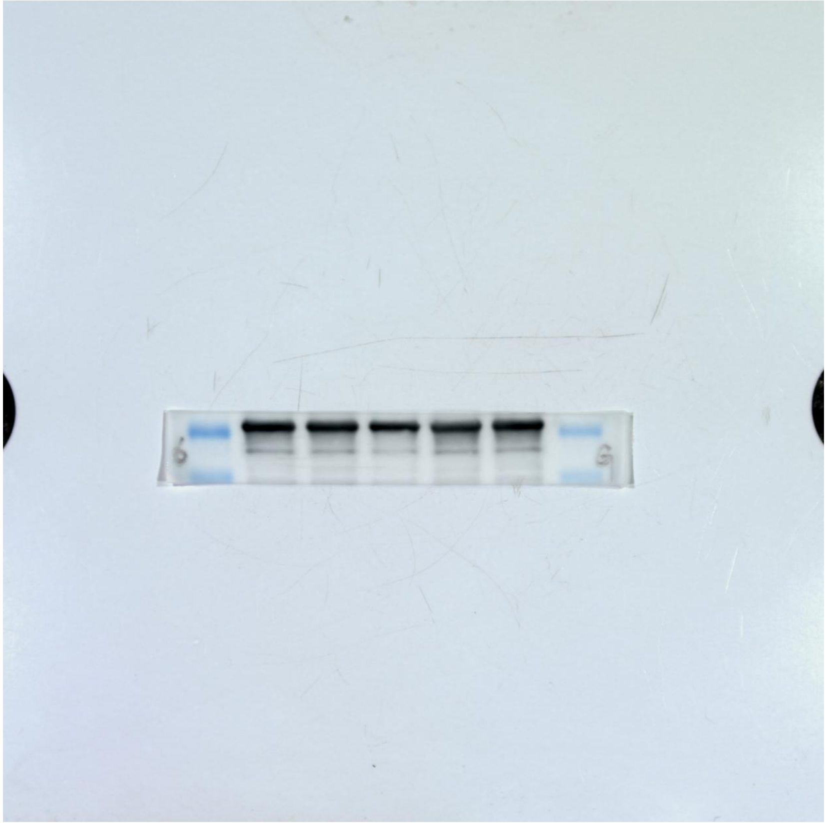

Supplement: Supplementary file 2 — Original Data File [file 41419_2025_7673_MOESM2_ESM.pdf]
